# Synthesis and Thermal Study of Hexacoordinated Aluminum(III) Triazenides for Use in Atomic Layer Deposition

**DOI:** 10.1021/acs.inorgchem.0c03496

**Published:** 2021-03-12

**Authors:** Rouzbeh Samii, David Zanders, Sydney C. Buttera, Vadim Kessler, Lars Ojamäe, Henrik Pedersen, Nathan J. O’Brien

**Affiliations:** † Department of Physics, Chemistry and Biology, Linköping University, SE-581 83 Linköping, Sweden; ‡ Faculty of Chemistry and Biochemistry, Ruhr University Bochum, Universitätsstraße 150, 44801 Bochum, Germany; § Department of Chemistry, Carleton University, 1125 Colonel By Drive, Ottawa, Ontario K1S5B6, Canada; ∥ Department of Molecular Sciences, Swedish University of Agricultural Sciences, P.O. Box 7015, 75007 Uppsala, Sweden

## Abstract

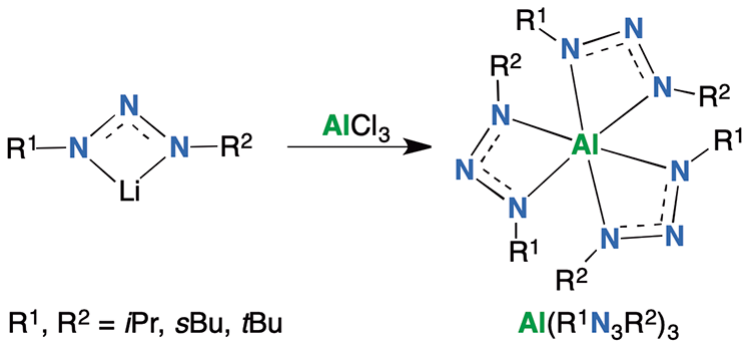

Amidinate and guanidinate ligands have been used extensively to produce volatile and thermally stable precursors for atomic layer deposition. The triazenide ligand is relatively unexplored as an alternative ligand system. Herein, we present six new Al(III) complexes bearing three sets of a 1,3-dialkyltriazenide ligand. These complexes volatilize quantitatively in a single step with onset volatilization temperatures of ∼150 °C and 1 Torr vapor pressures of ∼134 °C. Differential scanning calorimetry revealed that these Al(III) complexes exhibited exothermic events that overlapped with the temperatures of their mass loss events in thermogravimetric analysis. Using quantum chemical density functional theory computations, we found a decomposition pathway that transforms the relatively large hexacoordinated Al(III) precursor into a smaller dicoordinated complex. The pathway relies on previously unexplored interligand proton migrations. These new Al(III) triazenides provide a series of alternative precursors with unique thermal properties that could be highly advantageous for vapor deposition processes of Al containing materials.

## Abstract 2

Six aluminum(III) 1,3-dialkyltriazenides were synthesized and their thermal properties explored. The compounds were purified by recrystallization and could be easily sublimed. Thermogravimetric analysis of compounds showed that all, except one, gave a single step evaporation with onset volatilization temperatures of ∼150 °C. DFT calculations revealed a unique decomposition pathway that leads to a small and more reactive Al species that would be highly advantageous for the surface reactions of atomic layer deposition.

## 1 Introduction

Aluminum nitride (AlN) is a semiconductor material widely used in current day electronic devices.^1^ This is due to its desirable chemical, optical, and electronic properties, such as high thermal stability, a wide direct band gap, and piezoelectricity.^2^ As electronic devices rapidly miniaturize with increasingly complex surface structures, atomic layer deposition (ALD) becomes a vital technique for depositing uniform thin films of high-performance materials for future microelectronics.^3^ In ALD, the metal and nonmetal precursors are introduced into the reaction chamber separately, which allows the film mechanism to be governed by two independent and self-limiting half reactions. These are complex surface reactions that can incorporate impurities into the film if the metal precursor does not possess suitable physical and chemical properties. A desirable ALD metal precursor must be thermally stable until reaching the film surface.^4^ Here, it should undergo a clean and fast reaction to form a single stable monolayer without trapping unwanted byproducts.^3^ This monolayer should then react with the second precursor (e.g., NH_3_ or H_2_O) in the same way. To maximize the growth rate of a thin film, a precursor must be sufficiently volatile and have ligands of low steric bulk for fast surface saturation and maximum density of the deposited precursor. Due to its high volatility and reactivity, trimethylaluminum (AlMe_3_) has been used to deposit AlN by ALD.^5−13^ These films contain high levels of carbon impurities due to the strong Al–C bonds, making it difficult to remove all of the methyl ligands of the deposited precursor at low temperatures.^5,6^ Replacing the Al–C of AlMe_3_ with more reactive Al–N bonds has led to homoleptic tricoordinated amide precursors (Al(NMe_2_)_3_)^14^ and (Al(NEt_2_)_3_),^15^ which have been used to deposit AlN by ALD.^16−19^ Although these precursors are highly volatile and reactive, the low thermal stability of the deposited surface species renders films with carbon impurities.^20^

Amidinate and guanidinate bidentate ligands have been employed to improve thermal stability of group 13 metal precursors. Although these ligands improve thermal stability in comparison to monodentate ligated precursors, their drawback is compounds that lack volatility or surface reactivity, or both, the latter due to crowding of the metal center.^21−27^ In particular, homoleptic hexacoordinated M–N bonded Al(III) amidinate (Al(amd)_3_) and guanidinate (Al(guan)_3_) compounds possess increased thermal stability compared to tricoordinated Al(III) amides,^26,27^ but have not been used in an ALD process due to insufficient volatility. A ligand closely related to the amidinate and guanidinate is the triazenide, differing by the nitrogen atom in the endocyclic position of the ligand backbone. Homoleptic hexacoordinated Al(III) triazenide complexes have previously been reported;^29,30^ however, they are not volatile due to their 1,3-diphenyltriazenide ligands. Recently, we reported the first examples of highly volatile homoleptic 1,3-dialkyltriazenide complexes, tris(1,3-diisopropyltriazenide)In(III) (In(triaz)_3_)^31^ and Ga(III) (Ga(triaz)_3_),^32^ and their use as ALD precursors. These new triazenide precursors underwent gas-phase decomposition at higher temperatures inside the ALD reactor, giving a smaller and more reactive M(III) species. This *in situ* thermolysis was highly advantageous for film growth, giving higher growth rates and films with near stoichiometric M/N ratios without unwanted carbon impurities. To further explore the unique properties of the 1,3-dialkyltriazenide ligand, we envisaged its ability to stabilize the Al(III) center to develop a new series of precursors that can be used for future ALD processes. Herein, we describe the synthesis, structure, and thermal properties of six homoleptic Al(III) 1,3-dialkyltriazenide complexes. These compounds were easily synthesized in good yields and are the first example of volatile hexacoordinated M–N bonded aluminum compounds. Furthermore, the compounds exhibit unique thermal properties, similar to Ga(triaz)_3_ and In(triaz)_3_. Using quantum-chemical density functional theory (DFT) calculations, we mapped out a previously unexplored decomposition pathway utilizing interligand interactions. The pathway is supported by electron impact mass spectrometry (EI-MS) data. The unique thermal properties of these compounds make them potentially advantageous as precursors for vapor deposition processes.

## 2 Results and Discussion

### 2.1 Synthesis and Characterization of Aluminum Complexes

**Scheme 1:**
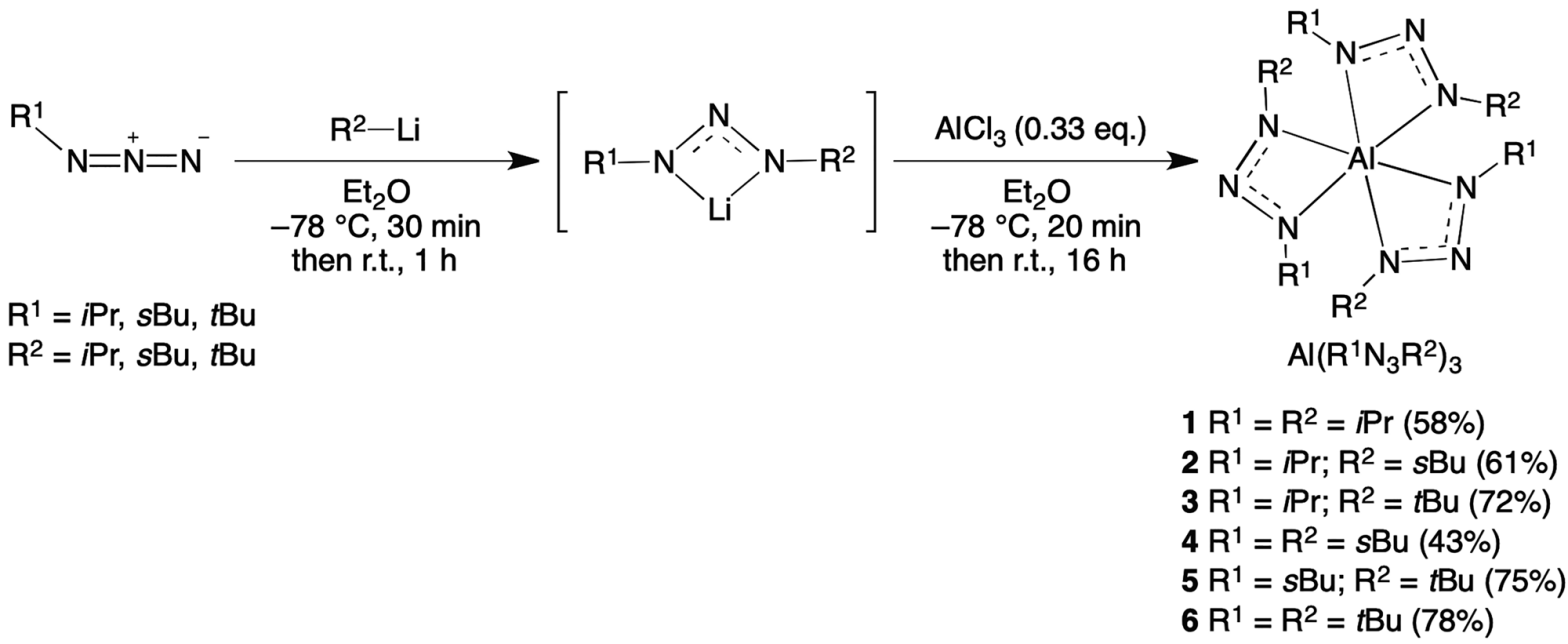
Synthesis of Tris(1,3-dialkyltriazenide)aluminum(III) Compounds **1**–**6**

Tris(1,3-dialkyltriazenide)aluminum(III) compounds **1**–**6** were prepared in good yields by reacting the (1,3-dialkyltriazenide)lithium(I) intermediate, generated from an alkylazide^33,34^ and alkyllithium, with AlCl_3_ (Scheme 1). All compounds were purified by recrystallization and were fully characterized by nuclear magnetic resonance (NMR) spectroscopy, elemental analysis (EA), sublimation temperature, and melting point. No decomposition was observed when stored under an inert atmosphere at room temperature for long periods of time. However, the compounds decomposed, without clear visual signs, when exposed to air and were no longer soluble in dry hexane. Presumably, the compounds formed nonsoluble mixed aluminum hydroxides and oxides.

Purification of the crudes of **1**–**6** by vacuum sublimation was unsuccessful due to impurities that cosublimed. We suspect that these impurities decomposed during sublimation to form white solids, which were insoluble in *n*-hexane, Et_2_O, THF, and toluene. Interestingly, the ^1^H NMR spectra of **1**–**6** showed no impurities after sublimation. However, satisfactory EA was not obtained from the sublimed and filtered solid. To obtain satisfactory EA, the compounds were therefore purified by recrystallization from Et_2_O/MeCN.

**Figure 1:**
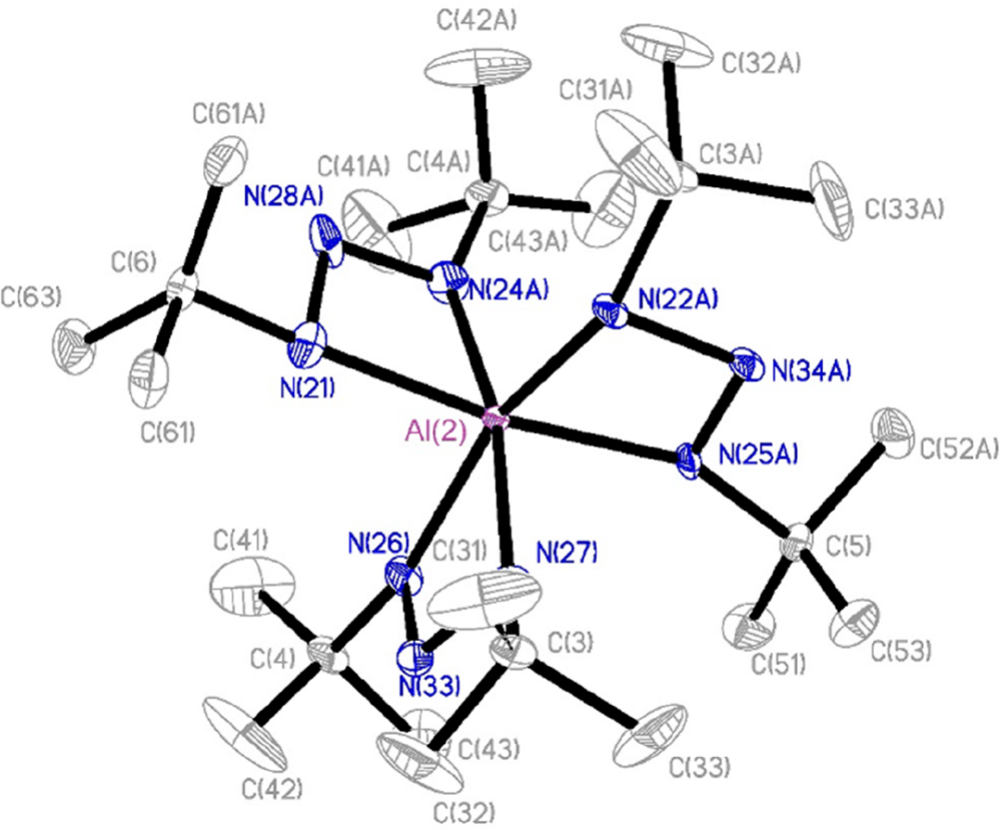
ORTEP drawing for one of two independent molecules in the unit cell of **6**. Thermal ellipsoids are displayed at the 50% probability level, and hydrogen atoms are omitted for clarity.

The crystal structure of **6** showed the aluminum in an octahedral coordination geometry bearing three sets of the 1,3-di-*tert*-butyltriazenide ligand (Figure 1). A large degree of disorder was observed in the diffraction data. The ligands are distorted over a multitude of positions (at least 8 individual sets of possible arrangements). The Al–N bond length for **6** (av 1.96(5) Å) are similar to that for tris(1,3-diphenyltriazenide)aluminum(III) (av 1.972(5) Å)^29^ but slightly shorter than those of Al(guan)_3_ (av 2.024 Å)^26^ and Al(amd)_3_ (av 2.0195 and 2.0261 Å).^35^ Thus, the triazenide ligand only has a small effect on the Al–N bond length in comparison to Al(guan)_3_ and Al(amd)_3_. Compound **1** has an analogous structure to that of **6**, but suffers from even more severe disorder and therefore no successful refinement could be completed (see the Supporting Information).

**Figure 2:**
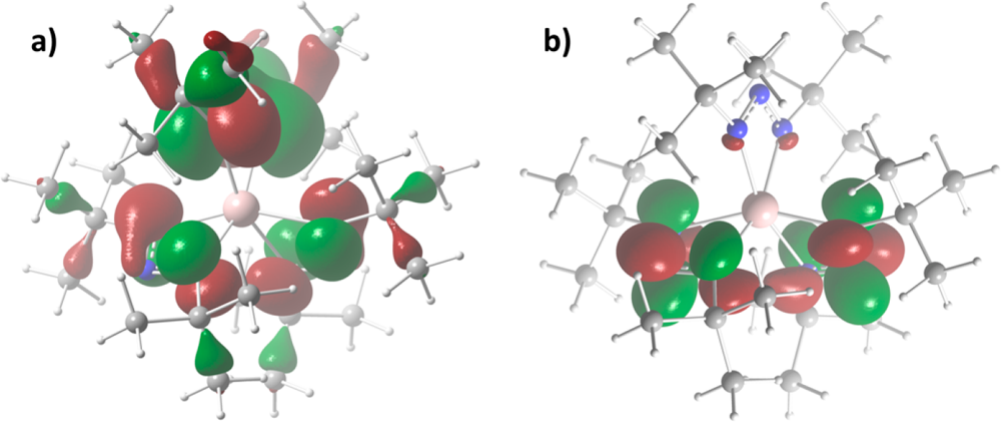
(a) HOMO (−5.74 eV) and (b) LUMO (−0.78 eV) for **6** from DFT calculations.

The DFT calculated geometry of **6** is consistent with its crystal structure. The highest occupied molecular orbital (HOMO) and lowest unoccupied molecular orbital (LUMO) are both localized on the ligands (Figure 2). While the HOMO is spread over all three ligands, the LUMO only covers the N_3_ backbone of two ligands. For the HOMO, one node is centered on the endocyclic nitrogen, while the LUMO has nodes between the endocyclic and exocyclic nitrogens. The natural charges of the exocyclic nitrogens (−0.48) and Al metal center (+1.66) indicate a highly polarized Al–N bond character. The primary carbons (−0.60) show negative charges, while the endocyclic nitrogens (+0.044) and tertiary carbons (+0.11) have slightly positive charges. Comparing the natural bond orbital charge of the Al center of **1** (1.61) and its formamidinate analogue (1.86) shows the greater electron donating ability of the triazenide ligand over the formamidinate (see the Supporting Information).

The ^27^Al NMR spectra of compounds **1**–**6** each gave a broad peak at δ_Al_ = 25.1–27.5 ppm (see the Supporting Information). These chemical shifts are consistent with previously reported hexacoordinated Al(III) triazenide complexes (δ_Al_ = 25–28 ppm).^29,30^ Dynamic effects were observed by ^1^H NMR for the unsymmetrical ligated compounds **2**, **3**, and **5** at 25 °C (see the Supporting Information). These effects are most likely caused by isomerization hindered by the bulky ligands surrounding the small Al(III) center.^36,37^ Therefore, the complexes isomerize slowly, resulting in significant lifetimes for the signals in relation to the difference in resonance frequencies.^38^ Heating resolves the lifetime broadening by increasing the rate of isomerization. Mild line broadening was observed for compound **2** at 25 °C and was resolved at 35 °C. Line splitting was observed for all but the C*H* signals for compounds **3** and **5**. These more severe effects are caused by the presence of the bulky *tert*-butyl groups, which further inhibit isomerization. The line splitting was resolved at 45 °C with only line broadening remaining. Heating to 50 °C resolved the line broadening for compound **3**. However, mild line broadening was still observed for **5**.

### 2.2 Thermal Analysis of Aluminum Complexes

**Figure 3:**
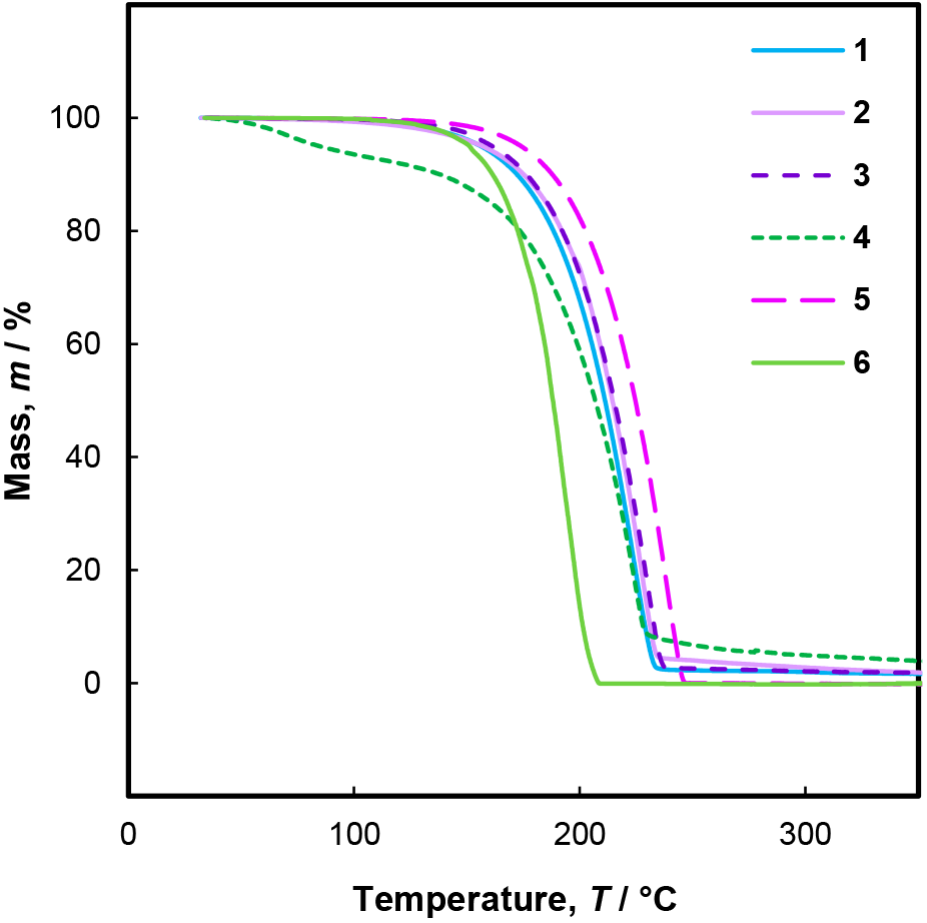
Thermogravimetric analysis of **1**–**6**.

Compounds **1**, **3**, **5**, and **6** volatilize quantitatively with exponential mass loss in thermogravimetric analysis (TGA) (Figure 3). Compound **2** has 4% residual mass by TGA, undergoing slight decomposition, as observed by an inflection in the derivative at approximately 200 °C (see the Supporting Information). TGA of **4** showed two distinct events of mass loss, giving 5–7% residual mass. We speculate that **4** decomposes into volatile fragments during the mass loss events. However, we were unable to obtain a satisfactory elemental analysis for **4**; therefore, the residual mass may be due to impurities. Overall, TGA shows that compounds **1**–**6** are sufficiently volatile for use in ALD. In fact, compounds **1**–**6** are far more volatile than Al(amd)_3_ and Al(guan)_3_,^26,27^ which can be explained by more electron density residing on the triazenide ligand. This leads to weaker intermolecular interactions in the crystal structure of the compound, which is conditional for faster volatilization (see the Supporting Information for a comparison of charges between **1** and its amidinate analogue).

Differential scanning calorimetry (DSC) was employed to study exothermic events of **1**–**6**. All compounds have exothermic events, most likely due to decomposition, overlapping with their onset of volatilization displayed in TGA (see the Supporting Information). The TGA and DSC results for **1**–**6** are summarized in Table 1. Compounds **1** and **5** have exothermic events starting at 150 and 160 °C, respectively. Both compounds give peaks with irregular shape, indicating overlapping exothermic events occurring. Two distinct exotherms are observed for **2**, **3**, and **6**: the first event initiates at 130, 160, and 230 °C, respectively, and the second between 234 and 300 °C. Compound **4** has a small exotherm starting at ∼100 °C, followed by a larger event at ∼200 °C. The unsymmetrical compounds **2**, **3**, and **5** undergo exothermic events at similar temperatures to **1**. That is, greater ligand bulk does not increase thermal stability of these compounds. However, the exothermic event of **6** is at significantly higher temperatures compared to **1**. Interestingly, **1** and **6** have similar calculated 1 Torr vapor pressure temperatures (Table 1).

**Table 1:** Summarized TGA and DSC Results for **1**–**6**

	1st DSC exotherm (°C)	onset of volatilization (°C)	1 Torr vapor pressure (°C)	residual mass (%)	sublimation temp^a^ (°C)
**1**	150–230	155	134	2	90
**2**	130–190	153	138	4	90
**3**	160–300	161	137	2	105
**4**	105–160	N/A	N/A	7	90
**5**	160–240	175	172	0	120
**6**	230–280	151	134	0	125

a Vacuum sublimation was undertaken at 0.5 mbar.

To study the long-term thermal stability of compounds **1**–**6**, their solids were each flamed sealed in an NMR tube and heated to 5 °C above their 1 Torr vapor pressure temperature (Table 1) for 7 days. After heat exposure, compounds **1**, **2**, and **5** fully dissolved in C_6_D_6_ while **3** and **6** had a small amount of insoluble solid. None of the compounds showed signs of decomposition by ^1^H NMR.

**Figure 4:**
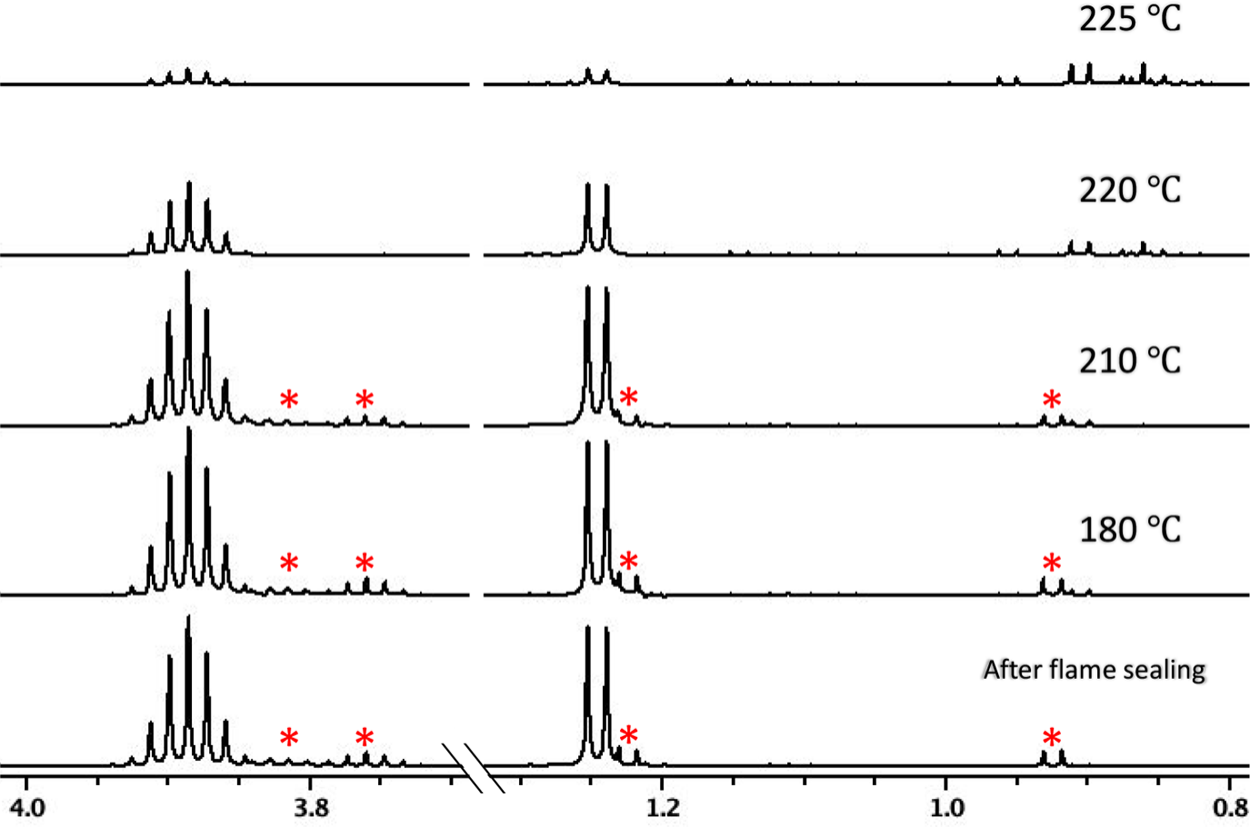
The ^1^H NMR (500 MHz, C_6_D_6_) spectra from a decomposition study of **1** between 0.8–1.3 and 3.7–4.0 ppm separated by an axis break. For visibility, the *y*-axis is scaled up ∼18 times on the left of the axis break compared to the right side. Prior to flame sealing, the compound showed no traces of impurities by ^1^H NMR analysis. The peaks marked with an asterisk appeared after flame sealing the tube. Compound **1** was heated in C_6_D_6_, and all spectra were acquired at 50 °C to suppress line broadening. The decomposition of **1** accelerates after 210 °C, which is shown by the diminished quartet and doublet peaks.

A solution of **1** in C_6_D_6_ was heated in a flame-sealed NMR tube to various temperatures for 1 h, followed by acquiring ^1^H NMR spectra (Figure 4). After flame sealing but before heating the sample, the ^1^H NMR spectrum showed newly formed, low-intensity impurity signals (red asterisk, Figure 4). Only small changes to **1** occurred when heating up to 180 °C, and the solution remained colorless. Above 210 °C, the solution turned yellow, and ^1^H NMR signals of the doublet and septet, at 1.25 and 3.88 ppm, respectively, decreased in intensity. A white precipitate formed at 225 °C, and the overall ^1^H NMR signal of **1** had decreased significantly. Traces of various decomposition products appear in the ranges 3.0–2.7, 2.4–2.5, and 2.0–0.7 ppm (see the Supporting Information).

### 2.3 Gas-Phase Decomposition by DFT Computations

**Figure 5:**
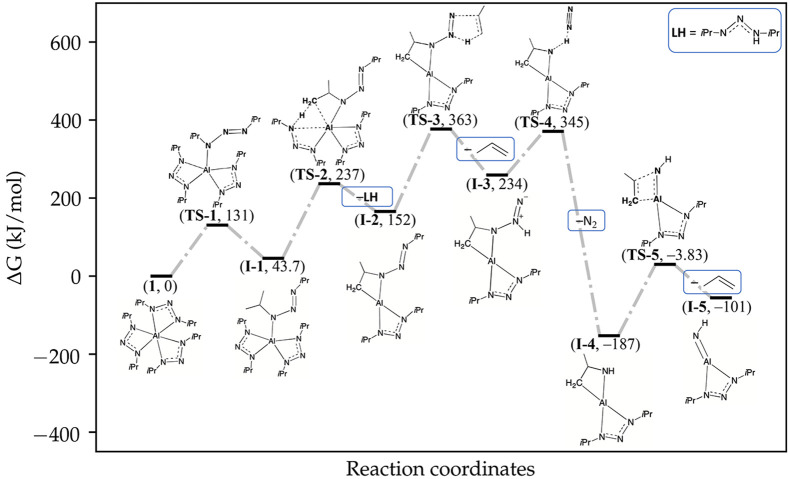
Free energy profile (at 250 °C and 10 hPa) for the first half of the decomposition pathway. Here, **1** loses a triazene ligand (after **TS-2**), and one ligand decomposes into an imido ligand (**TS-5**). **TS-3** has the largest free energy (211 kJmol^–1^) for the displayed part of the decomposition pathway. The overall largest free energy barrier is found at **TS-8** (214 kJmol^–1^): the analogous step to **TS-3** but for the last ligand. At 250 °C and 10 hPa, the adduct structures **I-2A** separate spontaneously (i.e., the process is barrierless and has a negative free energy difference) and is therefore not included.

**Figure 6:**
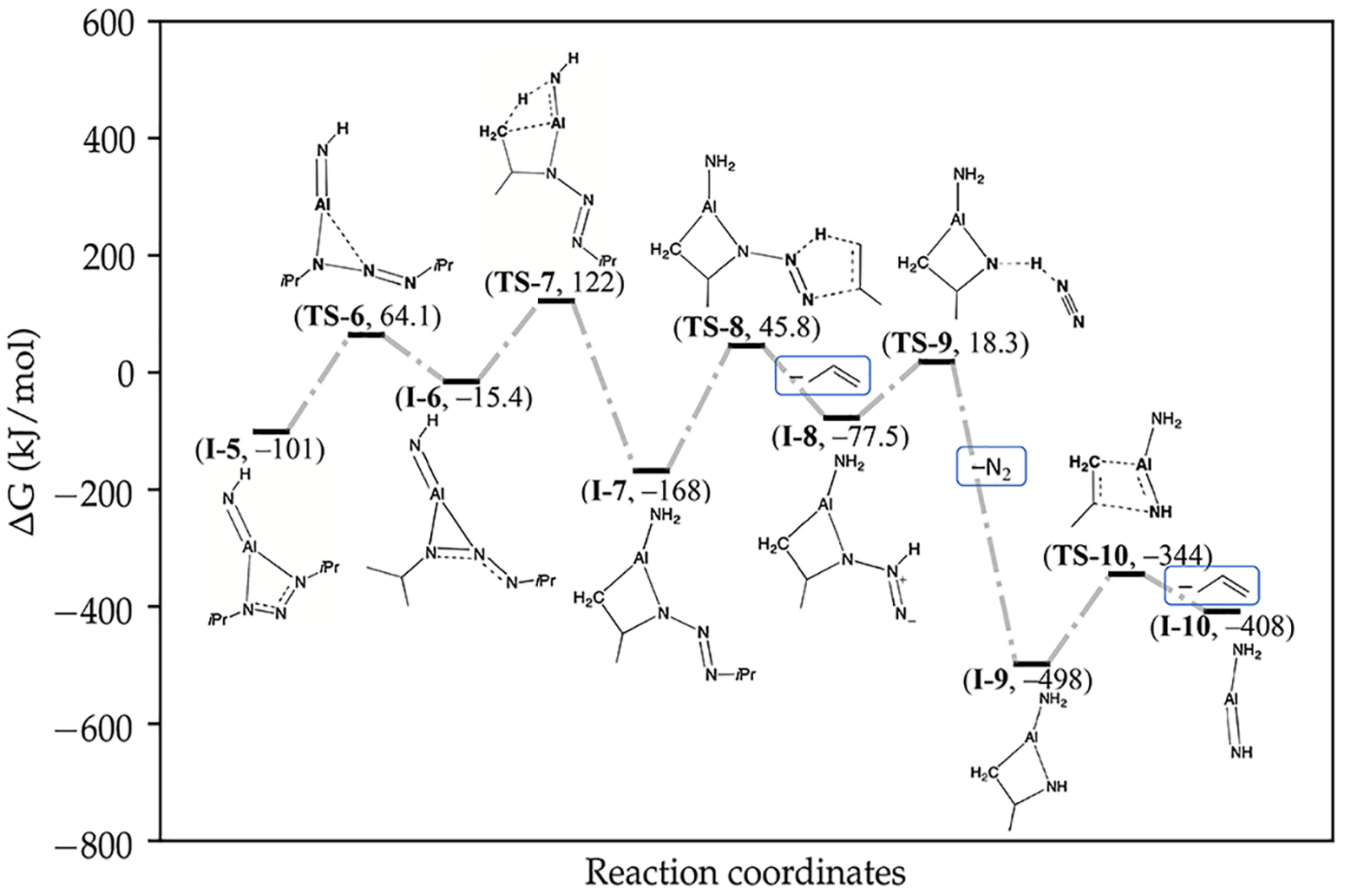
Free energy profile continuing from **I-5**. The steps that transform **I-5** into **I-10** are analogous to the steps that transform **1** into **I-5**. The reverse step through **TS-7** has a significantly larger free energy barrier compared to the analogous **TS-2** (290 vs 85 kJ mol^–1^, respectively).

In previous work, we demonstrated high-quality thin films of indium nitride and gallium nitride by ALD, using the Ga(triaz)_3_ and In(triaz)_3_ as precursors, respectively.^32,31^ From the thermal properties of the compounds, we speculated that the depositions are activated by gas-phase decomposition of the precursor in the ALD reactor. Compounds **1**–**6** have similar thermal properties as Ga(triaz)_3_ and In(triaz)_3_ and are therefore expected to undergo a similar decomposition. DFT was used to study gas-phase thermal decomposition pathways of **1**. We found a decomposition pathway relying on Brönsted–Lowry acid–base reactions between neighboring ligands. Overall, the first ligand leaves as triazene while the second decomposes into an imido ligand. Figure 5 shows the free energy profile for the release and decomposition of the first and second ligand, respectively, at 250 °C and 10 hPa. The third ligand decomposes in the same manner as the second, only with minor differences (Figure 6). The largest free energy barriers are 211 and 214 kJ mol^–1^, for **TS-3** (Figure 5) and **TS-8** (Figure 6), respectively. These barriers are slightly smaller for **6** (192 and 197 kJ mol^–1^). The Supporting Information contains pictures and Cartesian coordinates of all optimized geometries and their respective enthalpies and free energies.

Starting from **1**, a ligand dechelates from the metal center by a 180° rotation of a N–N bond (**TS-1**), resulting in **I-1**. This ligand dechelation enables the isopropyl moiety on the coordinated nitrogen to move closer to neighboring ligands and the metal center. Next, a methyl proton of the isopropyl group migrates to an exocyclic nitrogen on a neighboring ligand (**TS-2**). Simultaneously, a bond is formed between the deprotonated isopropyl group and the metal center. In **I-2A**, the deprotonated ligand regains a bidentate binding mode, now with a C,N-coordination to the metal center. Meanwhile, the protonated ligand dechelates and only has a coordination bond to the metal center. Due to the proton transfer in **TS-2**, the deprotonated and protonated ligand become dianionic and neutral, respectively. **I-2A** is an adduct structure consisting of a triazene (LH) coordinated to the **I-2** structure.

**Scheme 2:**
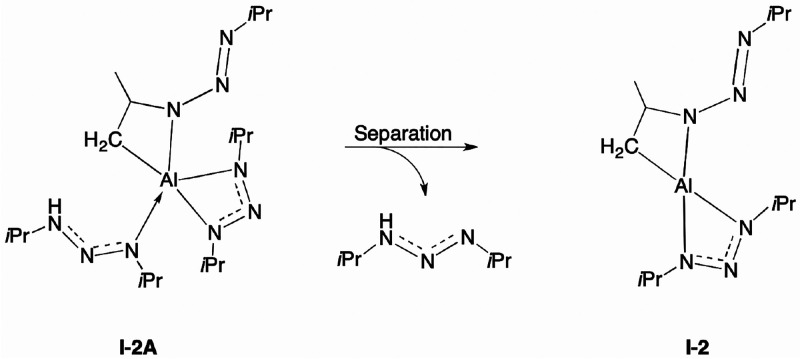
Separation of the Adduct Structure **I-2A** into a Triazene and **I-2** The **I-2** intermediate has one monoanionic N,N-coordinated and one dianionic C,N-coordinated triazenide ligand.

The two steps that transform **1** into **I-2A** are reversible. In contrast, separating **I-2A** into LH and **I-2** may be reversible or irreversible depending on the reaction conditions (Scheme 2). **I-2A** separates spontaneously under reduced pressure and elevated temperature, conditions commonly employed in ALD. Therefore, we assume that when the adduct structure separates, LH becomes inaccessible to **I-2** and cannot facilitate the backward reaction, i.e., making the forward reaction irreversible.

**I-2** has two triazenide ligands: the dianionic C,N-coordinated ligand and an unaltered N,N-coordinated ligand. The dianionic ligand decomposes in three irreversible steps, transforming the C,N-coordinated triazenide into an imido ligand (Scheme S2). First, a methyl proton migrates from the isopropyl group on the β-nitrogen to the α-nitrogen, with respect to the coordinated nitrogen (**TS-3**). This proton transfer results in a molecule of propene leaving the structure, giving **I-3**. Second, the proton on the α-nitrogen migrates to the coordinated nitrogen (**TS-4**), releasing dinitrogen to give **I-4**. Third, passing through **TS-5**, the former isopropyl group, which coordinated to the metal center in **TS-2**, leaves as propene to give **I-5**.

Other than the newly formed imido ligand, **I-5** has one intact triazenide ligand that decomposes in a similar fashion as the first (Figure 6). Moving from **I-5** toward **TS-6**, the intact triazenide dechelates by rotating 180° along a N–N bond, breaking the four-membered ring with the metal center. This step is analogous to the dechelation that transform **1**, via **TS-1**, into **I-1**. In contrast to **I-1**, however, where the ligand remained monodentate, **I-6** is a less crowded structure and allows the triazenide ligand to regain a bidentate binding mode. In **I-6**, two adjacent nitrogen atoms of the triazenide ligand bind to the metal center, forming a three-membered ring. For the interligand proton migration (**TS-7**) to occur, the ligand must adopt a monodentate binding mode. When approaching **TS-7** from **I-6**, the three-membered ring open and the ligand becomes monodentate without passing a transition state. Next, the second interligand proton transfer of the decomposition pathway occurs (**TS-7**). This step is similar to the first (**TS-2**) except that, now, the neighboring imido ligand acts as the Brønsted–Lowry base instead of a neighboring triazenide ligand. Furthermore, this step has a significantly lower free energy barrier compared to **TS-2** due to the imido being a much stronger base compared to the triazenide ligand. For the same reason, the free energy barrier for the reverse reaction via **TS-7** is very large (290 kJ mol^–1^), essentially blocking the backward reaction. After passing **TS-7**, the deprotonated and protonated ligands transform into a dianionic C,N-coordinated triazenide and an amido ligand, respectively, to give **I-7**.

The second dianionic triazenide decompose into an imido ligand via **TS-8** to **TS-10**, which are analogous to **TS-3** to **TS-5**, giving the adduct structure **I-10A**. The adduct separates spontaneously into the final structure, **I-10**, and propene. A lack of viable options for **I-10** to further decompose makes it thermally stable in the gas phase. However, based on the structure, **I-10** is expected to be highly reactive toward surfaces. EI-MS data for **1** show four signals consistent with fragments for intermediates of the presented decomposition pathway (see the Supporting Information). Three potential fragments are identified for **6** and these fragments are structurally analogous to fragments found for **1**. Furthermore, both compounds give a low intensity signal (∼1%) at *m/z* 58, matching **I-10**. The calculated and found *m/z* for all fragments are given in Table 2.

**Table 2:**
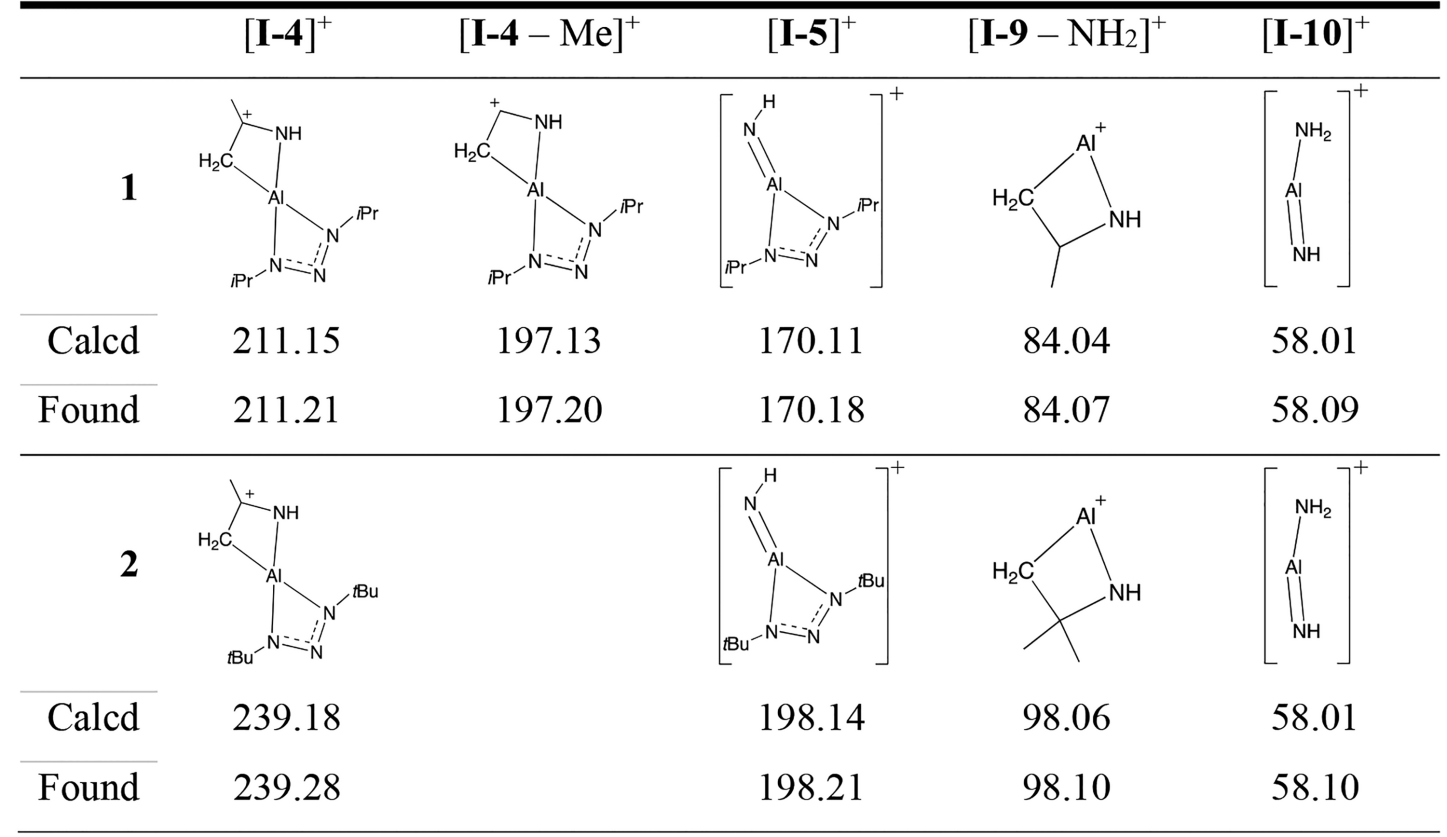
Summarized EI-MS Signals (Given in *m/z*) And Their Matching Intermediate Fragments from the Presented Decomposition Pathway for Compounds **1** and **6**

## 3 Conclusion

In conclusion, six tris(1,3-dialkyltriazenide)aluminum(III) compounds have been made in good yields. The crystal structure of **6** revealed a homoleptic complex with three sets of the 1,3-di-*tert*-butyltriazenide ligand chelating to the Al(III) center. The complexes are highly volatile and, with the exception of **4**, volatilize in a single step with exponential mass loss. Exponential mass loss indicated that the compounds volatilize without decomposing. However, DSC revealed exothermic events, most likely due to decomposition, overlapping with the temperature range for the mass loss event in TGA. Therefore, the compounds may undergo decomposition upon or after volatilization. Using DFT, a gas-phase decomposition pathway was found that relies on protons migrating between ligands. One ligand leaves the complex as a molecule of triazene, and the two remaining ligands transform into C,N-coordinated dianionic triazenide ligands that decompose into imido ligands. The largest free energy barrier for the pathway is 214 kJ mol^–1^ (at 10 hPa and 250 °C) during decomposition of the second dianionic ligand (Figure 6, **TS-8**). A slightly lower free energy barrier of 211 kJ mol^–1^ (at 10 hPa and 250 °C) is found for **TS-3** (decomposition of the first ligand, analogous to **TS-8**). The final intermediate of the pathway is predicted to be highly reactive. Further studies are required to form a better understanding of how the compounds behave during the mass loss events observed in TGA. To our knowledge, compounds **1**–**6** are the first hexacoordinated Al–N bonded compounds that are sufficiently volatile for use as Al precursors in vapor deposition. Based on thermal analysis and DFT calculations, we postulate that the compounds decompose into smaller and more reactive species in the gas phase, which would be highly beneficial for ALD.

## 4 Experimental Section

### 4.1 General Experimental Procedures

*Caution! As catenated nitrogen compounds are known to be associated with explosive hazards, alkylazides and compounds***1**–**6***are possible explosive energetic materials. Although we have not experienced any problems in the synthesis, characterization, sublimation, heating, and handling of compounds***1**–**6***, their energetic properties have not been fully investigated and are therefore unknown. We therefore highly recommend that all appropriate standard safety precautions for handling explosive materials (safety glasses, face shield, blast shield, leather gloves, polymer apron, and ear protection) be used at all times when working with isopropyl-, sec-butyl-, and tert-butylazide and compounds***1**–**6**.

All reactions and manipulations were carried out under a N_2_ atmosphere on a Schlenk line using Schlenk air-free techniques, or in a N_2_-filled drybox from Glovebox-Systemtechnik. All anhydrous solvents were purchased from Sigma-Aldrich and further dried with molecular sieves 4 Å. Isopropyllithium (0.7 M in pentane), *sec*-butyllithium (1.4 M in cyclohexane), and *tert*-butyllithium (1.7 M in pentane) were purchased from Sigma-Aldrich, and AlCl_3_ (99.985%) was purchased from Alfa Aesar; all were used without further purification. *Tert*-butyl-, *sec*-butyl-, and isopropylazide were synthesized according to previously reported literature procedures.^33,34^ All NMR spectra were measured with Oxford Varian 300 and AS500 spectrometers at room temperature unless otherwise stated. Solvents’ peaks were used as an internal standard for the ^1^H NMR (300 and 500 MHz) and ^13^C NMR (75 and 125 MHz) spectra. For temperature stability measurements using NMR, a 40 g L^–1^ solution of **1** in C_6_D_6_ was added to a heavy-walled NMR tube, and the tube was flame-sealed. EI-MS data for **1** and **6** were acquired using a Varian MAT spectrometer operated at 70 eV in the electron ionization mode. Samples were filled into steel cartridges, sealed with lids, and individually fed to the spectrometer via a load-lock chamber which was pumped to ultrahigh vacuum prior to sample transfer to the main chamber. Melting points were determined for samples under N_2_ atmosphere, flame-sealed in capillaries, using a Stuart SMP10 melting point apparatus and are uncorrected. Elemental analysis was performed by Mikroanalytisches Laboratorium Kolbe, Germany. Purification of compounds **1**–**6** by sublimation gave unsatisfactory EA results. Satisfactory EA results were obtained by recrystallizing the sublimed compounds.

### 4.2 General Synthesis Procedure for Al(III) Triazenide Complexes

Alkyllithium (3 equiv) was added to a solution of alkyl azide (3 equiv) in Et_2_O at −78 °C, and the reaction mixture was stirred at this temperature for 30 min and then at room temperature for 1 h. This solution was then added to a −78 °C solution of AlCl_3_ (1 equiv) in Et_2_O via cannula. The reaction mixture was stirred at this temperature for 30 min and then slowly warmed to room temperature and stirred for 16 h. The reaction mixture was then concentrated under reduced pressure, and the resulting residue was suspended in *n*-hexane. Solids were filtered off through a pad of Celite and concentrated under reduced pressure to give the crude product. Purifying the crude product by sublimation resulted in unsatisfactory purity by EA. The crude product was therefore purified by recrystallization from Et_2_O/MeCN at −35 °C to give the desired tris(1,3-dialkyltriazenide)aluminum(III) complexes, **1**–**6**.

#### Tris(1,3-diisopropyltriazenide)aluminum(III) (**1**)

Compound **1** was synthesized according to the general procedure using isopropyl azide (0.42 g, 4.93 mmol) in Et_2_O (25 mL), isopropyllithium (7.05 mL, 4.93 mmol), and AlCl_3_ (0.22 g, 1.65 mmol) in Et_2_O (25 mL). The solid was purified by recrystallization to give **1** as a solid (0.39 g, 58%).

**1**: Colorless solid, mp 255–257 °C. Sublimation: 90 °C (at 0.5 mbar). ^1^H NMR (300 MHz, C_6_D_6_): δ 1.25 (d, *J* = 6.7 Hz, 36H, C*H*_3_), 3.88 (sept, *J* = 6.7 Hz, 6H, C*H*). ^13^C{^1^H} NMR (75 MHz, C_6_D_6_): δ 23.4 (s, *C*H_3_), 52.6 (s, *C*H). ^27^Al NMR (78 MHz, C_6_D_6_): δ 25.1 (br s). EI-MS (LR): 43.1 (60%), 84.1 (4.3%), 156.2 (15%), 170.3 (1.4%), 197.2 (9.3%), 211.2 (19%), 283.3 (30%). Anal. Calcd for C_18_H_42_AlN_9_: C, 52.53%; H, 10.29%; N, 30.63%. Found: C, 51.70%; H, 10.31%; N, 30.04%.

#### Tris(1-isopropyl-3-*sec*-butyltriazenide)aluminum(III) (**2**)

Compound **2** was synthesized according to the general procedure using *sec*-butyl azide (0.59 g, 5.95 mmol) in Et_2_O (30 mL), isopropyllithium (8.50 mL, 5.95 mmol), and AlCl_3_ (0.26 g, 1.98 mmol) in Et_2_O (30 mL). The solid was purified by recrystallization to give **2** as a solid (0.55 g, 61%).

**2**: Colorless solid, mp 187–192 °C. Sublimation: 90 °C (at 0.5 mbar). ^1^H NMR (300 MHz, C_6_D_6_): δ 0.92 (t, *J* = 7.5 Hz, 9H, C*H*_3_), 1.26 (d, *J* = 6.6 Hz, 18H, C*H*_3_), 1.26 (d, *J* = 6.6 Hz, 9H, C*H*_3_), 1.42–1.60 (m, 3H, C*H*_2_), 1.71–1.88 (m, 3H, C*H*_2_), 3.63 (sext, *J* = 6.6 Hz, 3H, C*H*), 3.87 (sept, *J* = 6.6 Hz, 3H, C*H*). ^13^C{^1^H} NMR (75 MHz, C_6_D_6_): δ 11.3 (s, *C*H_3_), 20.2 (br s, *C*H_3_), 23.4 (s, *C*H_3_), 30.7 (s, *C*H_2_), 52.4 (s, *C*H), 58.7 (s, *C*H). ^27^Al NMR (78 MHz, C_6_D_6_): δ 26.0 (br s). Anal. Calcd for C_21_H_48_AlN_9_: C, 55.60%; H, 10.67%; N, 27.79%. Found: C, 53.92%; H, 10.46%; N, 26.42%.

#### Tris(1-isopropyl-3-*tert*-butyltriazenide)aluminum(III) (**3**)

Compound **3** was synthesized according to the general procedure using *tert*-butyl azide (0.38 g, 3.83 mmol) in Et_2_O (20 mL), isopropyllithium (5.48 mL, 3.83 mmol), and AlCl_3_ (0.17 g, 1.28 mmol) in Et_2_O (20 mL). The solid was purified by recrystallization to give **3** as a solid (0.42 g, 72%).

**3**: Colorless crystals, mp >300 °C. Sublimation: 105 °C (at 0.5 mbar). ^1^H NMR (300 MHz, C_6_D_6_, 50 °C): δ 1.26 (d, *J* = 6.5 Hz, 18H, C*H*_3_), 1.34 (s, 27H, C*H*_3_), 3.82 (sept, *J* = 6.6 Hz, 3H, C*H*). ^13^C{^1^H} NMR (75 MHz, C_6_D_6_, 50 °C): δ 23.4 (br s, *C*H_3_), 30.7 (s, *C*H_3_), 52.1 (s, *C*H), 56.4 (s, *C*_q_). ^27^Al NMR (78 MHz, C_6_D_6_): δ 25.5 (br s). Anal. Calcd for C_21_H_48_AlN_9_: C, 55.60%; H, 10.67%; N, 27.79%. Found: C, 55.07%; H, 10.71%; N, 27.54%.

#### Tris(1,3-di-*sec*-butyltriazenide)aluminum(III) (**4**)

Compound **4** was synthesized according to the general procedure using *sec*-butyl azide (0.29 g, 2.93 mmol) in Et_2_O (20 mL), *sec*-butyllithium (2.09 mL, 2.93 mmol), and AlCl_3_ (0.13 g, 0.98 mmol) in Et_2_O (20 mL). The solid was purified by sublimation at 90 °C to give **4** as a semisolid (0.21 g, 43%).

**4**: Colorless semi-solid. Sublimation: 90 °C (at 0.5 mbar). ^1^H NMR (300 MHz, C_6_D_6_, 50 °C): δ 0.93 (t, *J* = 7.5 Hz, 18H, C*H*_3_), 1.28 (d, *J* = 6.0 Hz, 18H, C*H*_3_), 1.42–1.63 (m, 6H, C*H*_2_), 1.75–1.88 (m, 6H, C*H*_2_), 3.62 (sext, *J* = 6.6 Hz, 6H, C*H*). ^13^C{^1^H} NMR (75 MHz, C_6_D_6_, 50 °C): δ 11.3 (s, *C*H_3_), 20.2 (s, *C*H_3_), 30.7 (s, *C*H_2_), 58.6 (s, *C*H). ^27^Al NMR (78 MHz, C_6_D_6_, 50 °C): δ 27.5 (br s). A satisfactory elemental analysis could not be obtained for compound **4**.

#### Tris(1-*sec*-butyl-3-*tert*-butyltriazenide)aluminum(III) (**5**)

Compound **5** was synthesized according to the general procedure using *tert*-butyl azide (0.34 g, 3.43 mmol) in Et_2_O (20 mL), *sec*-butyllithium (2.45 mL, 3.43 mmol), and AlCl_3_ (0.15 g, 1.14 mmol) in Et_2_O (20 mL). The solid was purified by recrystallization to give **5** as a solid (0.43 g, 75%).

**5**: Colorless crystals, mp >300 °C. Sublimation: 120 °C (at 0.5 mbar). ^1^H NMR (300 MHz, C_6_D_6_, 50 °C): δ 0.92 (t, *J* = 7.4 Hz, 9H, C*H*_3_), 1.27 (d, *J* = 6.6 Hz, 9H, C*H*_3_), 1.35 (s, 27H, C*H*_3_), 1.40–1.62 (m, 3H, C*H*_2_), 1.75–1.94 (m, 3H, C*H*_2_), 3.51–3.66 (m, 3H, C*H*). ^13^C{^1^H} NMR (755 MHz, C_6_D_6_, 50 °C): δ 10.4–12.1 (m, *C*H_3_), 17.9–19.7 (m, *C*H_3_), 30.5–30.9 (m, *C*H_3_), 31.0 (s, *C*H_2_), 56.4 (*C*_q_), 57.5–58.6 (m, *C*H). ^27^Al NMR (78 MHz, C_6_D_6_): δ 26.0 (br s). Anal. Calcd for C_24_H_54_AlN_9_: C, 58.15%; H, 10.98%; N, 25.43%. Found: C, 55.92%; H, 10.66%; N, 24.42%.

#### Tris(1,3-di-*tert*-butyltriazenide)aluminum(III) (**6**)

Compound **6** was synthesized according to the general procedure using *tert*-butyl azide (0.45 g, 4.54 mmol) in Et_2_O (25 mL), *tert*-butyllithium (2.67 mL, 4.54 mmol), and AlCl_3_ (0.20 g, 1.51 mmol) in Et_2_O (25 mL). The solid was purified by recrystallization to give **6** as a solid (0.59 g, 78%).

**6**: Colorless crystals, mp >300 °C. Sublimation: 125 °C (at 0.5 mbar). ^1^H NMR (300 MHz, C_6_D_6_): δ 1.38 (s, 54H, C*H*_3_). ^13^C{^1^H} NMR (75 MHz, C_6_D_6_): δ 31.3 (s, *C*H_3_), 57.3 (s, *C*_q_). ^27^Al NMR (78 MHz, C_6_D_6_): δ 23.7 (br s). EI-MS (LR): 41.1 (3.7%), 57.1 (19%), 98.1 (1.1%), 198.2 (9.9%), 239.3 (2.2%), 339.4 (100%), 495.5 (0.51%). Anal. Calcd for C_24_H_54_AlN_9_: C, 58.15%; H, 10.98%; N, 25.43%. Found: C, 58.21%; H, 10.96%; N, 25.41%.

### 4.3 X-ray Crystallographic Analysis

Colorless single crystals for **6** were obtained by recrystallization from *n*-hexanes at −35 °C. The single crystals were used for X-ray diffraction data collection on a Bruker D8 SMART Apex-II diffractometer, using graphite-monochromated Mo Kα radiation (λ = 0.710 73 Å) at 153 K. All data were collected in hemisphere with over 95% completeness to 2θ < 50.05°. The structure is monoclinic, centrosymmetric, space group *C*2/*m*, *a* = 28.940(5), *b* = 16.958(3), *c* = 9.8864(17) Å, β = 94.118(2)°. In spite of data collection at low temperature, the data produce an electron density map that is rather “flat”, which is a result of very heavy disorder, rendering an almost amorphous structure. The structure was solved by direct methods. Coordinates of metal atoms were determined from the initial solutions, and from the N and C methods, located in subsequent differential Fourier syntheses. The solution does not contain much residual electron density, but it stays for a multitude of additional possible positions of light atoms. All nonhydrogen atoms were refined, first in isotropic and then in anisotropic approximation, using Bruker SHELXTL software. Additional crystal data treatment details are available from the Cambridge Crystallographic Data Centre, deposition no. CCDC 2046808.

The data collection on crystals of **1** was carried out under same conditions as for **6**. An even less featured electron density map was obtained from the reflections provided by an analogous structure, monoclinic centrosymmetric, space group *C*2/*m*, *a* = 26.639(25), *b* = 15.817(15), *c* = 9.184(9) Å, β = 95.069(14)°. A model analogous to that for **6** was obtained but could not be refined successfully because of poor data quality. The details for the model obtained for the structure of **1** are available in the Supporting Information.

### 4.4 Thermogravimetric Analysis

Volatilization and vapor pressure curves were collected using a TA Instruments thermogravimetric analysis Q500 tool operating inside a N_2_-filled glovebox. The ramp experiment of compounds **1**–**6** was undertaken in tared platinum pans loaded with ∼5–10 mg for low mass volatilization experiments. The furnace was heated at a rate of 10 °C min^–1^ to 500 °C with a maintained N_2_ flow rate of 60 sccm. The Langmuir vapor pressure equations for compounds **1**–**4** and **6** were derived from TGA mass-loss derivative data of the ramp experiments according to a previously reported method^39^ employing bis(2,2,6,6-tetramethyl-3,5-heptanedionato)copper(II) as a calibrant.^40^

### 4.5 Differential Scanning Calorimetry Analysis

DSC measurements were performed using a TA Instruments DSC Q10 tool. For each compound, **1**–**6**, 0.2–0.5 mg of the compound was sealed in a platinum pan in a N_2_-filled glovebox. All experiments were performed at a heating rate of 10 °C min^–1^ between 25 and 400 °C. Exothermic and endothermic events are indicated by positive and negative heat flow, respectively.

### 4.6 Quantum-Chemical Computations

All quantum-chemical computations were performed using Gaussian 16 software.^41^ Structural optimization and harmonic normal mode vibrational calculations were performed using the hybrid DFT method B3LYP^42,43^ together with Grimme’s version 3 dispersion correction^44^ and def2TZVP^45,46^ basis set. The decomposition pathway was investigated by searching for possible stable structures as well as finding transition states connecting these structures. Minima were confirmed to have no imaginary frequencies, while transition states were verified to have one imaginary frequency, lying along the reaction path.

## Supporting Information Available

Characterization of the compounds and computational calculation details (PDF)
Compound **1** (CIF)
Compound **6** (CIF)

## Supplementary Material

ic0c03496_si_001.pdf

ic0c03496_si_003.cif

ic0c03496_si_004.cif

## Accession Codes

CCDC 2046808 contains the supplementary crystallographic data for this paper. These data can be obtained free of charge via www.ccdc.cam.ac.uk/data_request/cif, or by emailing data_request@ccdc.cam.ac.uk, or by contacting The Cambridge Crystallographic Data Centre, 12 Union Road, Cambridge CB2 1EZ, UK; fax: +44 1223 336033.
