# Supplementary material for: Synthesis and Thermal Study of Hexacoordinated Aluminum(III) Triazenides for Use in Atomic Layer Deposition
Source: Inorg Chem. 2021 Mar 12;60(7):4578–87. doi: 10.1021/acs.inorgchem.0c03496 (PMC8041287; doi:10.1021/acs.inorgchem.0c03496)
Supplement: Supplementary file 1 — ic0c03496_si_001.pdf [file ic0c03496_si_001.pdf]

Supporting information
for
Synthesis and Thermal Study of Hexacoordinated Aluminum(III) Triazenides for Use in Atomic Layer Deposition

Rouzbeh Samii,* David Zanders,[†,‡] Sydney C. Buttera,[‡] Vadim Kessler,[§] Lars Ojamäe,* Henrik Pedersen,* and Nathan J. O'Brien[ǁ,]*

[*]Department of Physics, Chemistry and Biology, Linköping University, SE-581 83 Linköping, Sweden

[†]Faculty of Chemistry and Biochemistry, Ruhr University Bochum, Universitätsstraße 150, 44801, Bochum, Germany

[‡]Department of Chemistry, Carleton University, 1125 Colonel By Drive, Ottawa, Ontario, K1S5B6, Canada

[§]Department of Molecular Sciences, Swedish University of Agricultural Sciences, P.O. Box 7015, 75007 Uppsala, Sweden

[ǁ]E-mail: nathan.o.brien@liu.se

Table of contents

NMR spectral charts	S1–12
EI-MS for **1** and **6**	S13
TGA curves and plots for calculating 1 Torr vapor pressure	S14–16
DSC data	S17–18
Computational Details	S19–78

NMR Spectral charts

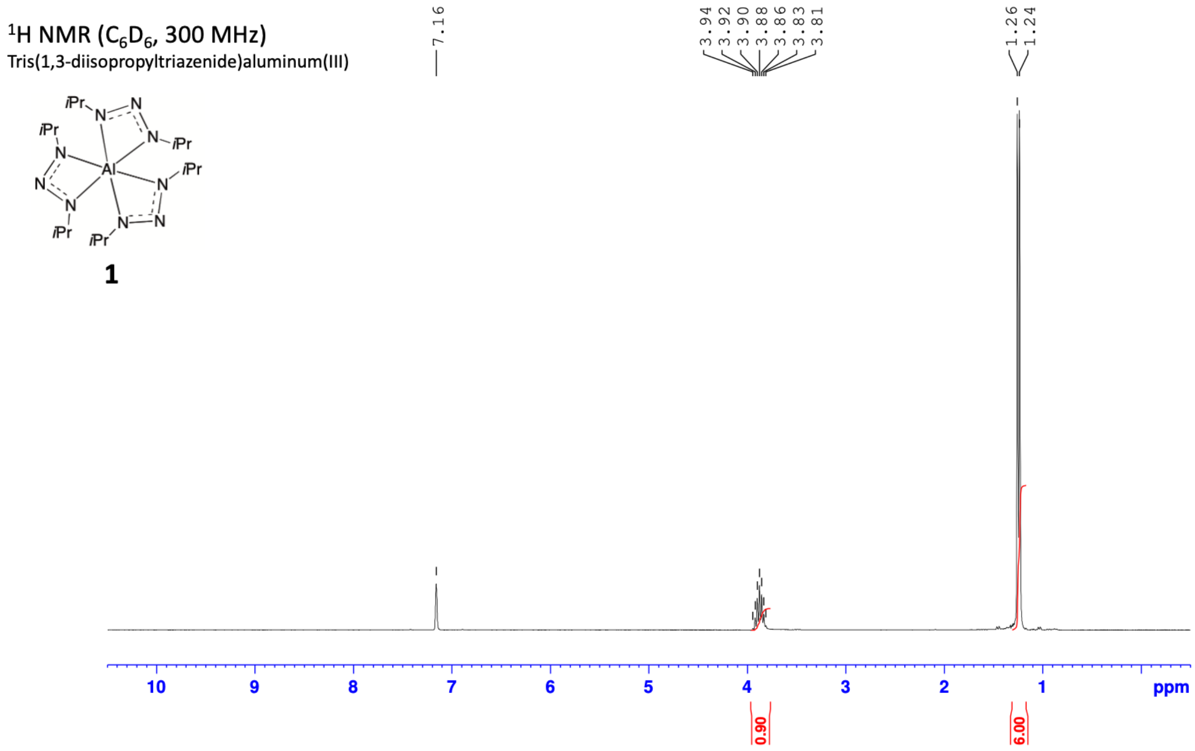

Figure S1: ¹H NMR spectrum of **1**.

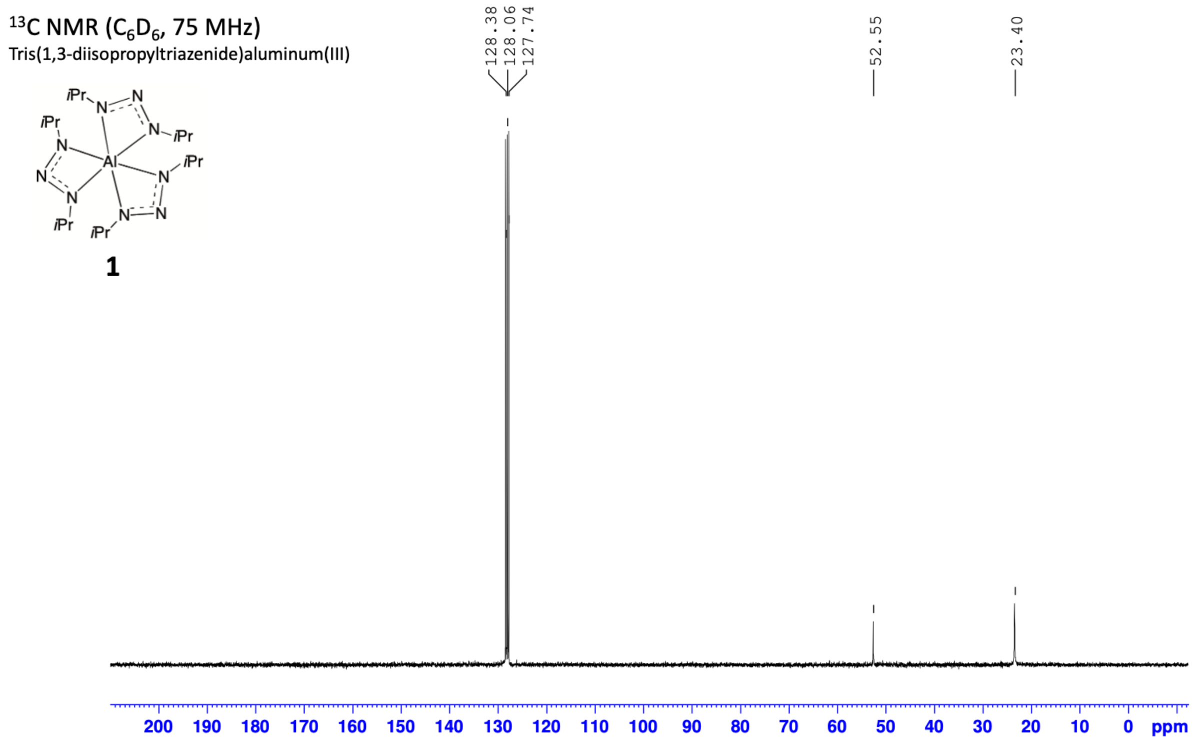

Figure S2: ¹³C NMR spectrum of **1**.

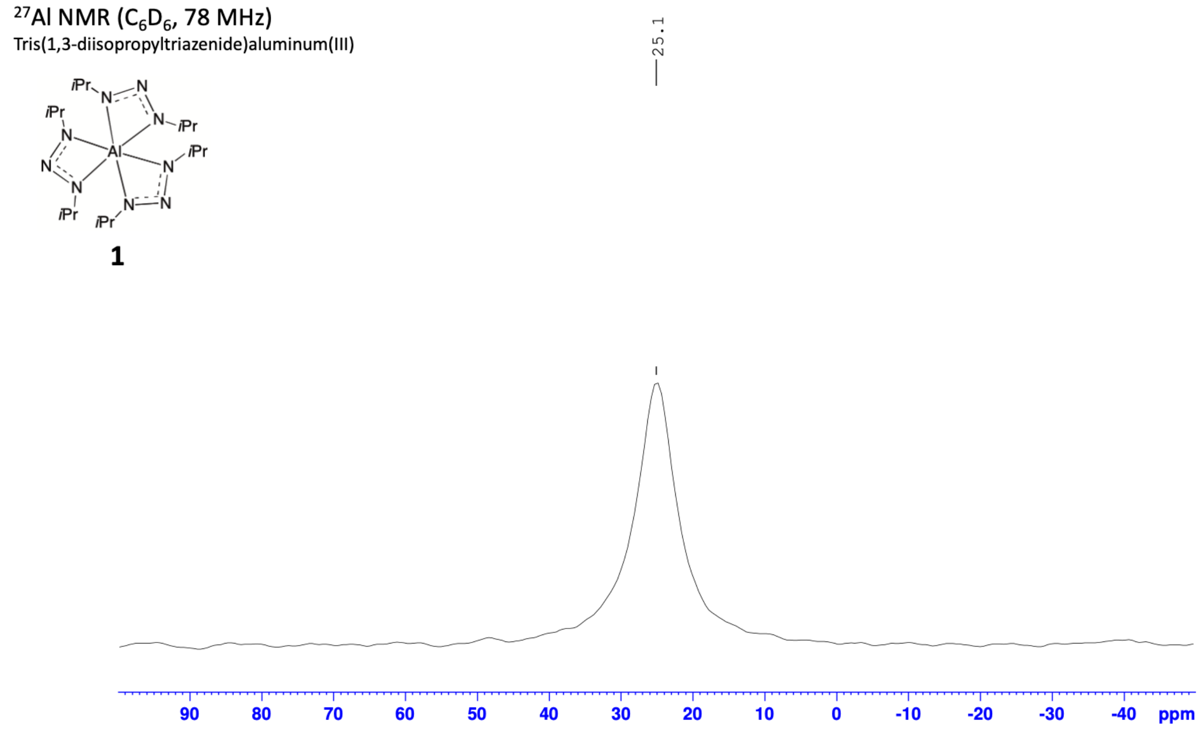

Figure S3: ^{27}Al NMR spectrum of **1**.

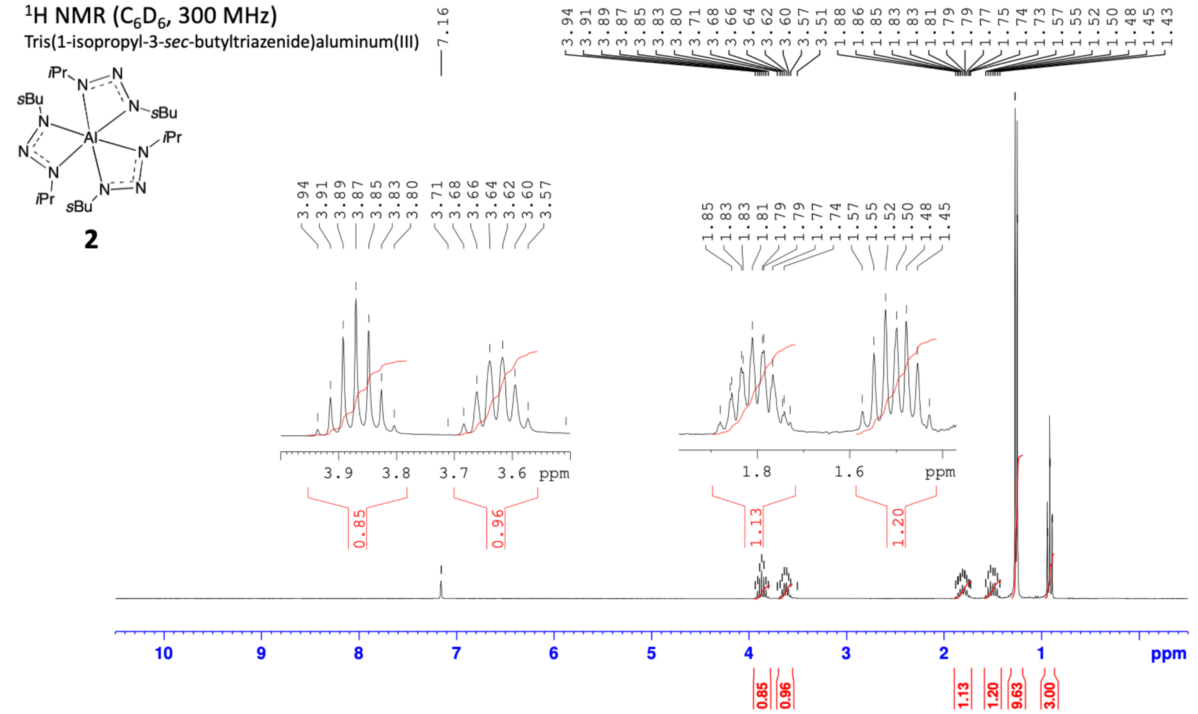

Figure S4: ^{1}H NMR spectrum of **2** at 50 °C.

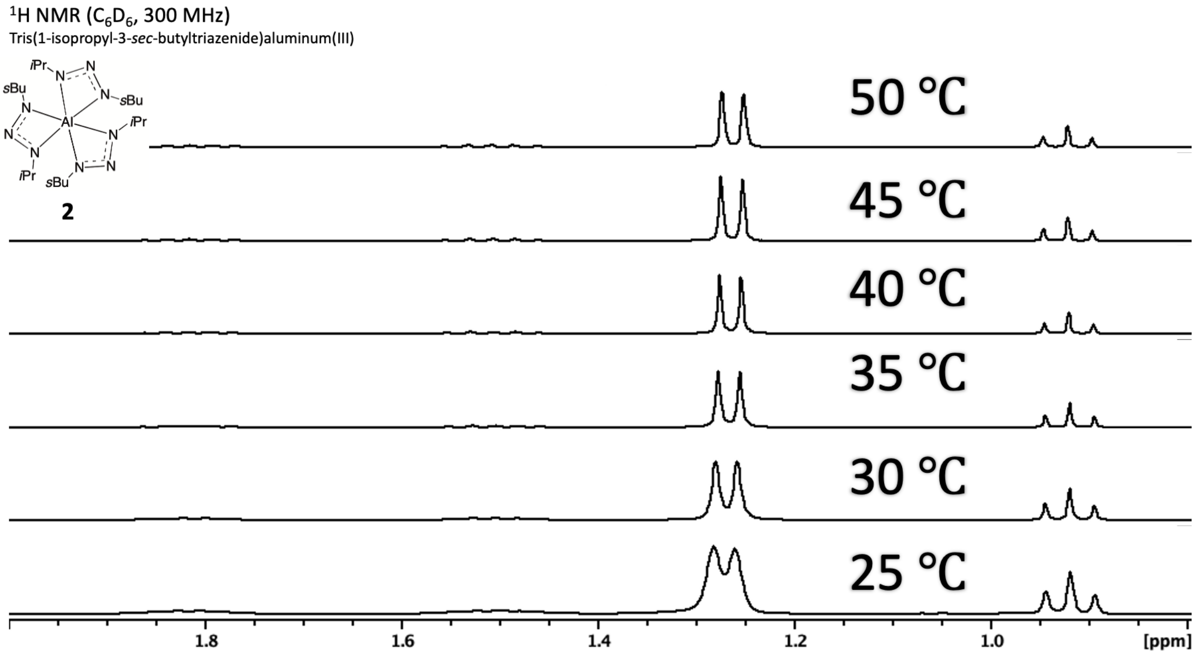

Figure S5: ^{1}H NMR VT spectrum of **2**.

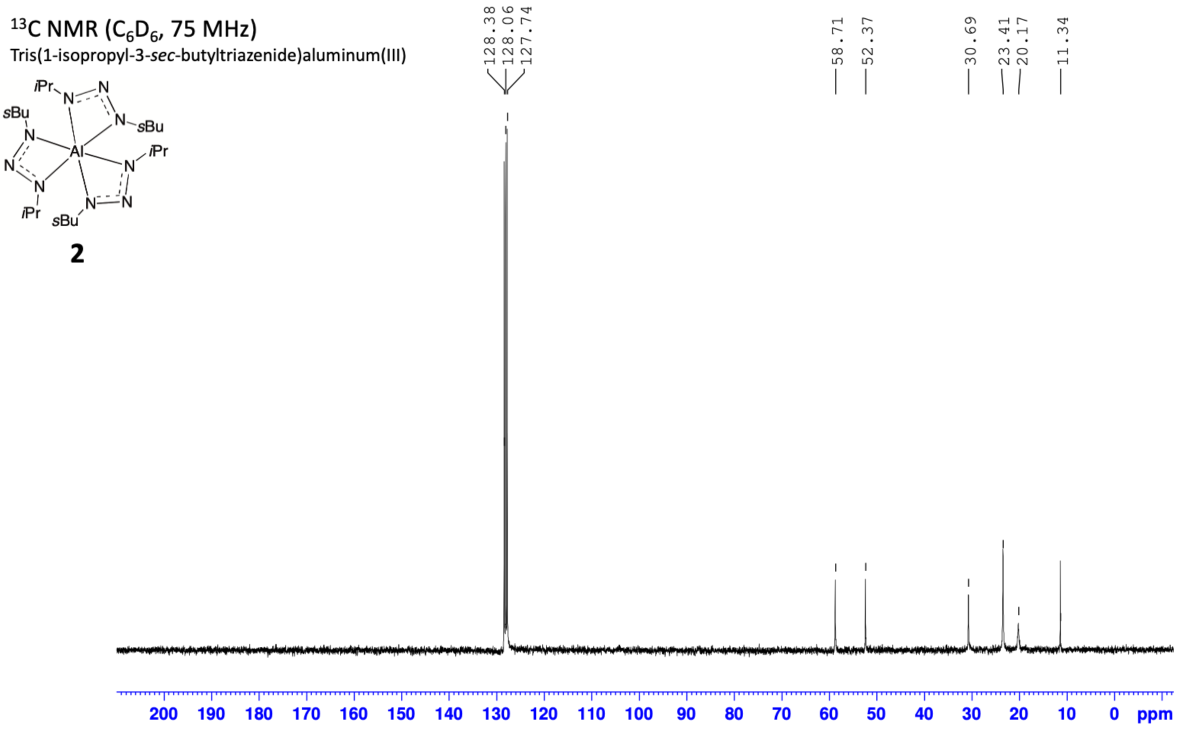

Figure S6: ^{13}C NMR spectrum of **2** at 50 °C.

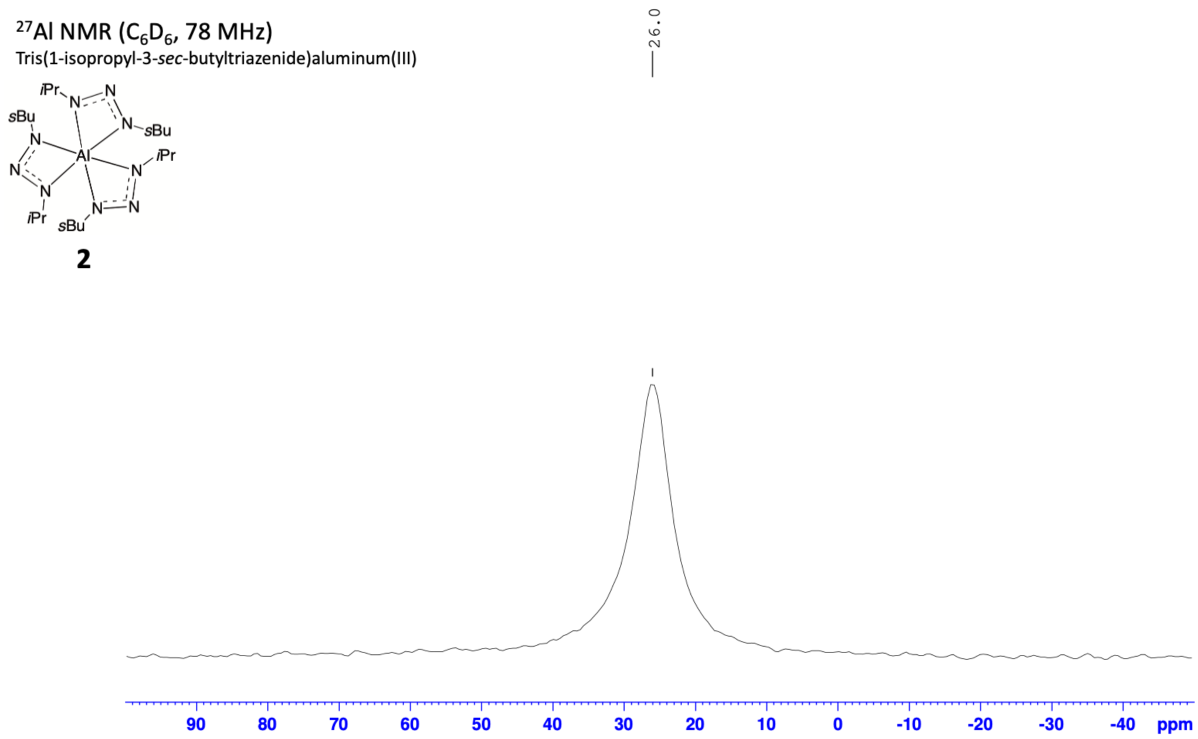

Figure S7: ^{27}Al NMR spectrum of **2** in C_6D_6.

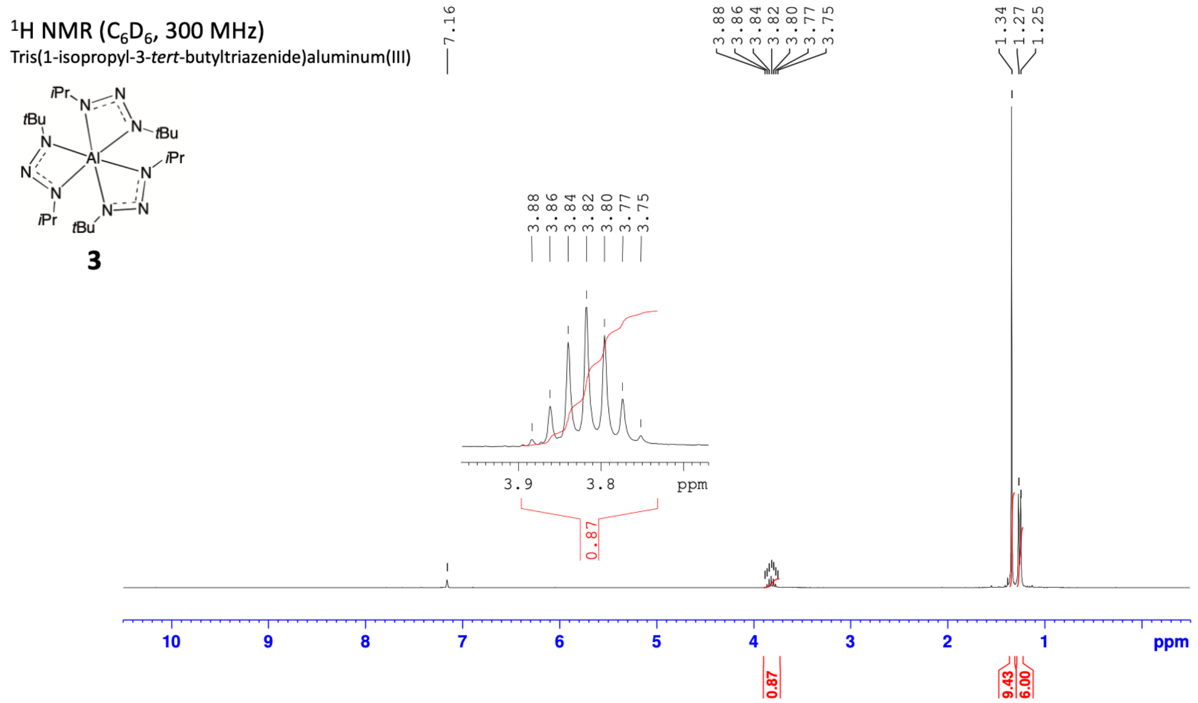

Figure S8: ^{1}H NMR spectrum of **3** at 50 °C.

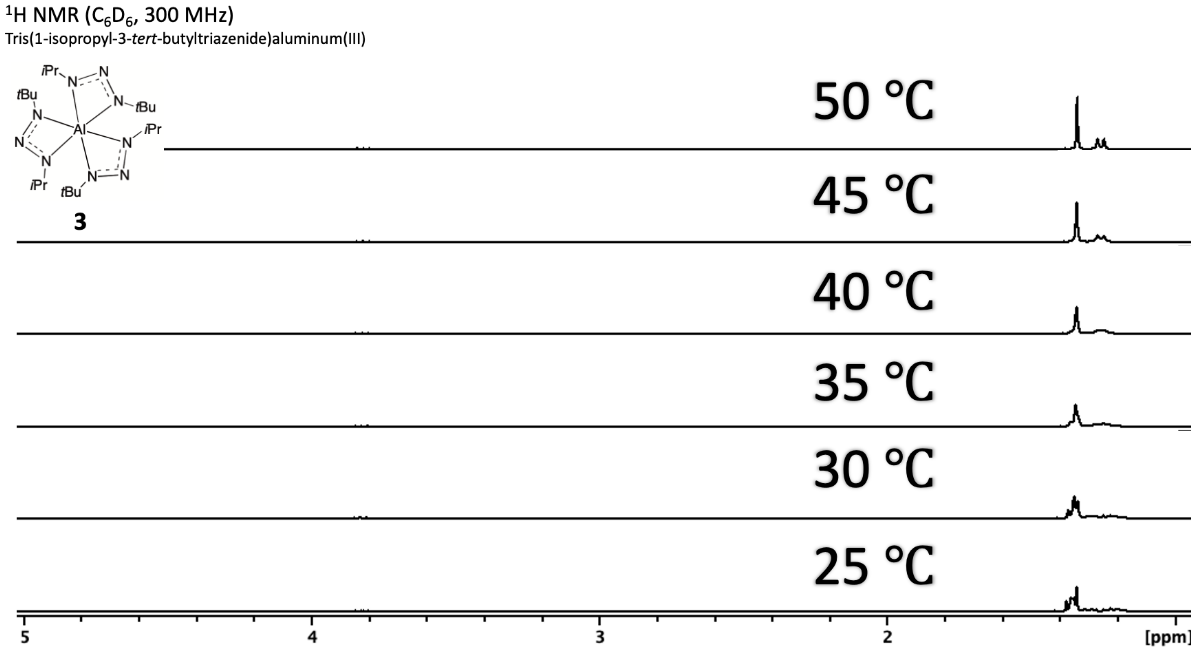

Figure S9: ^{1}H NMR VT spectrum of **3**.

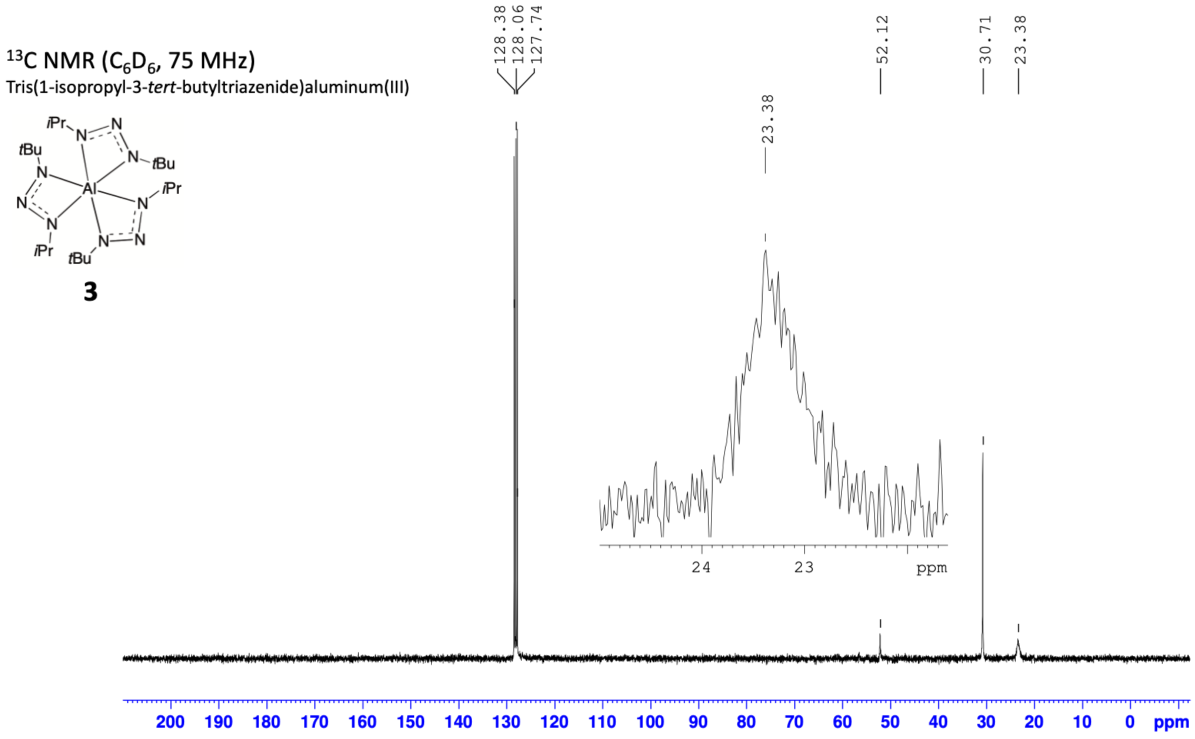

Figure S10: ^{13}C NMR spectrum of **3** at 50 °C. The *tert*-butyl $C(CH_3)_3$ is missing.

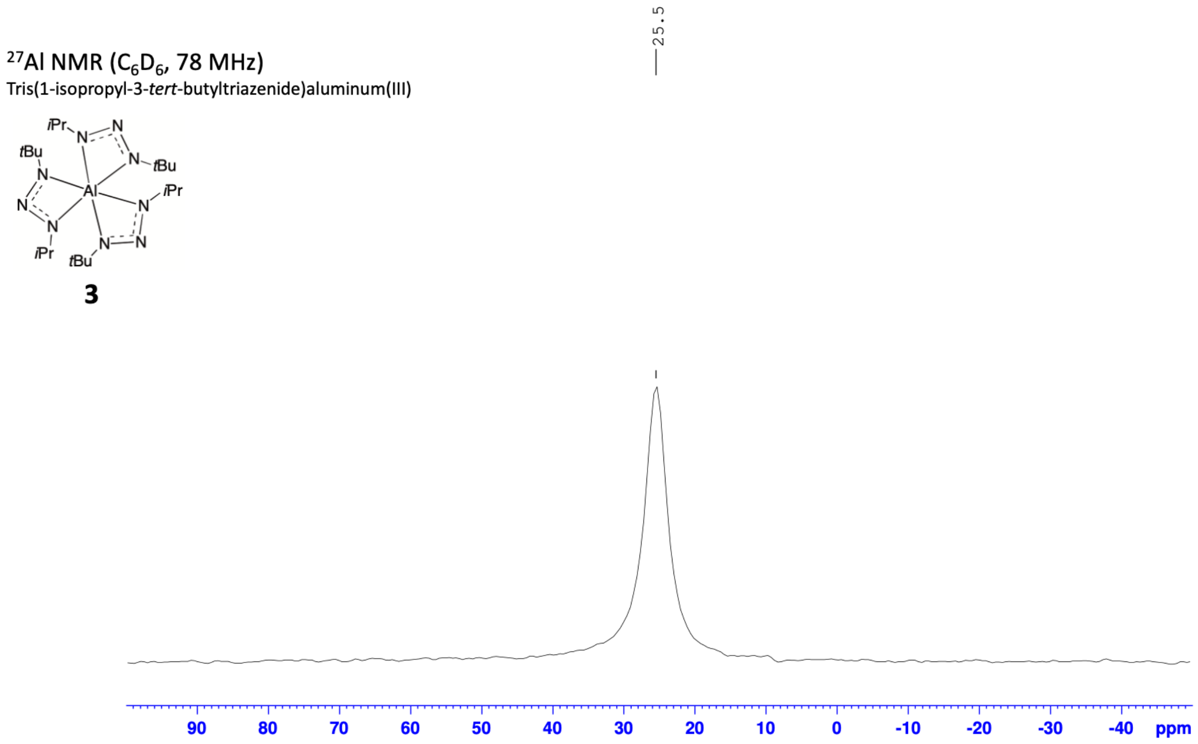

Figure S11: ^{27}Al NMR spectrum of **3** at 50 °C.

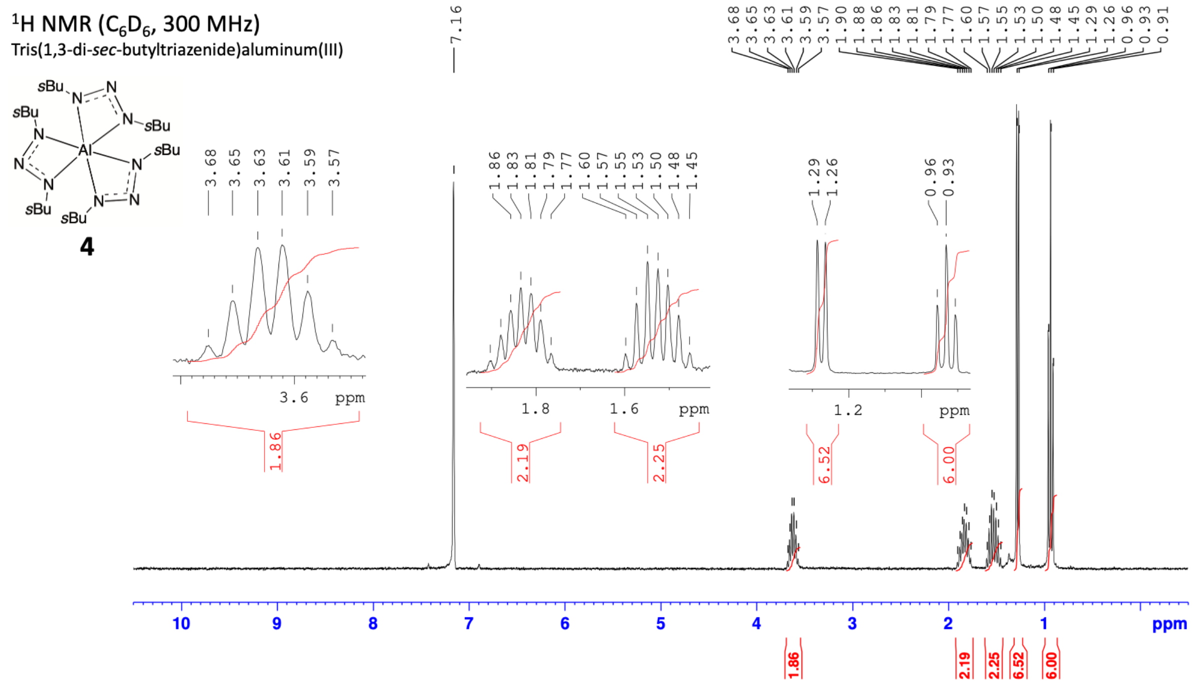

Figure S12: ^{1}H NMR spectrum of **4**.

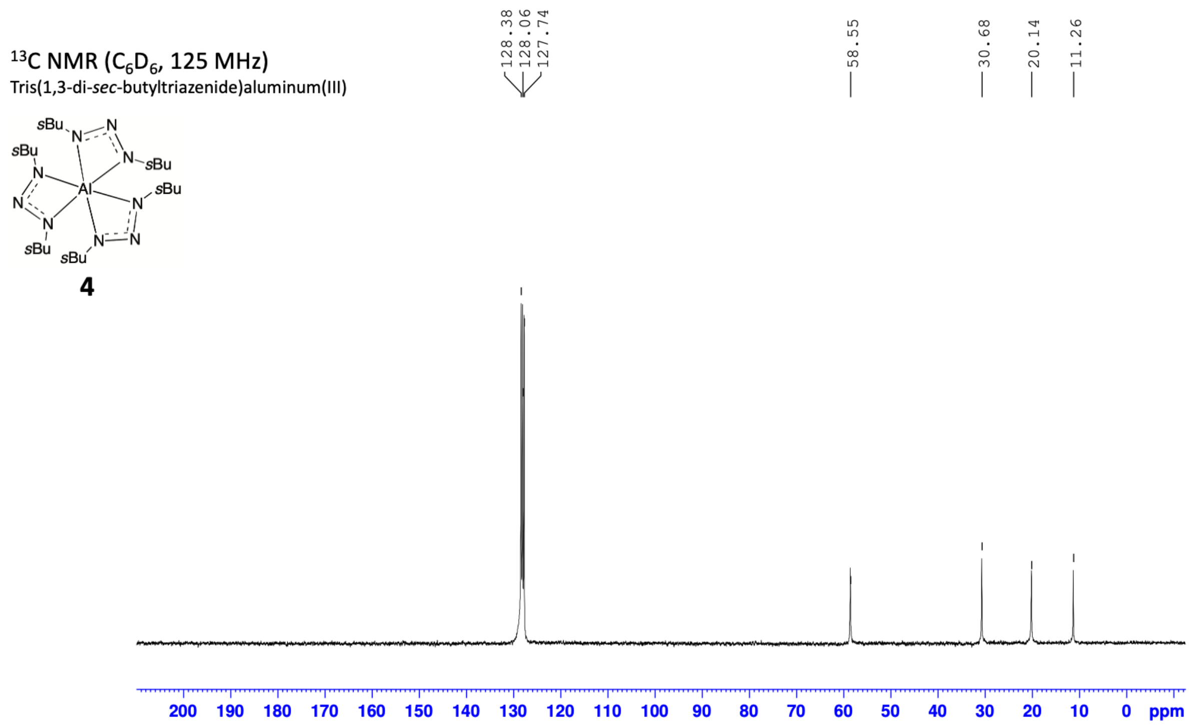

Figure S13: ^{13}C NMR spectrum of **4**.

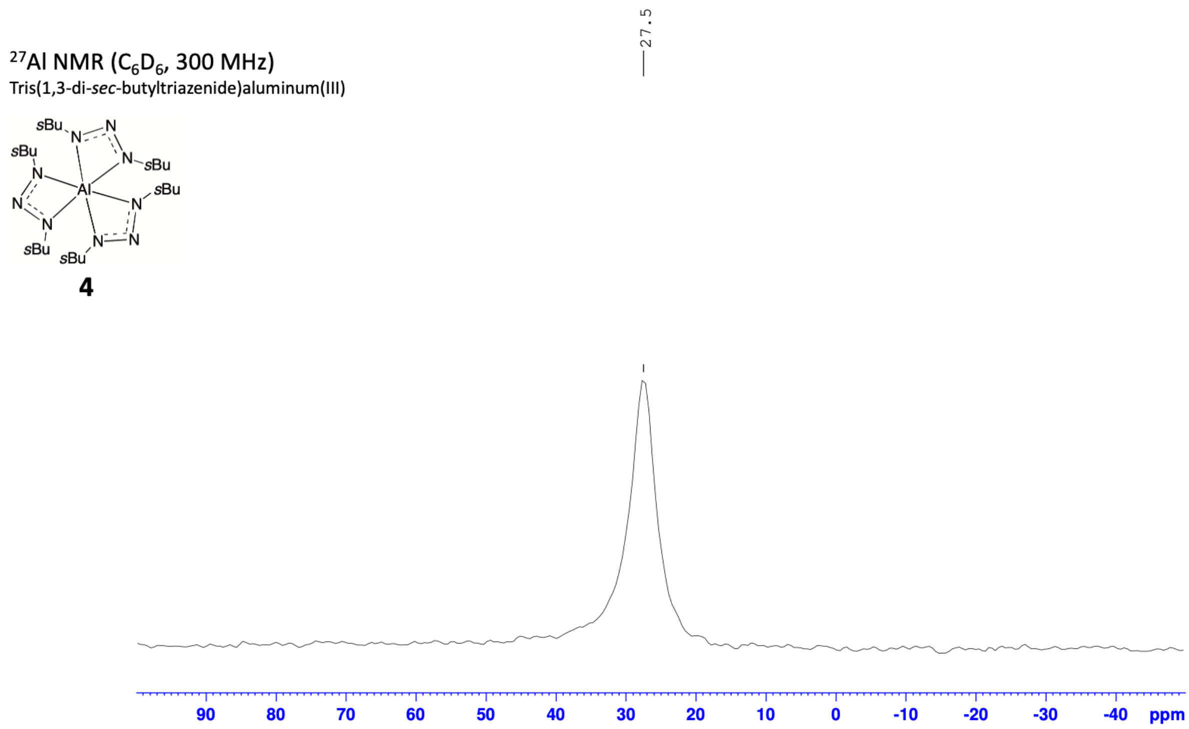

Figure S14: ^{27}Al NMR spectrum of **4** at 50 °C.

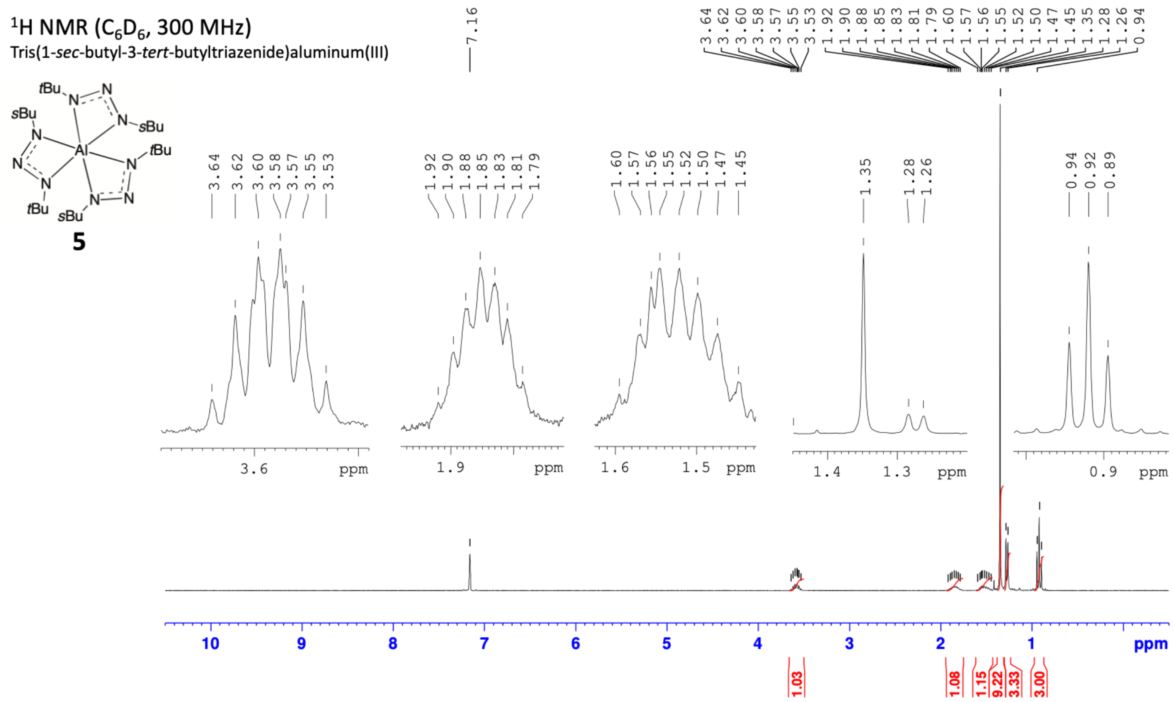

Figure S15: ^{1}H NMR spectrum of **5** at 50 °C.

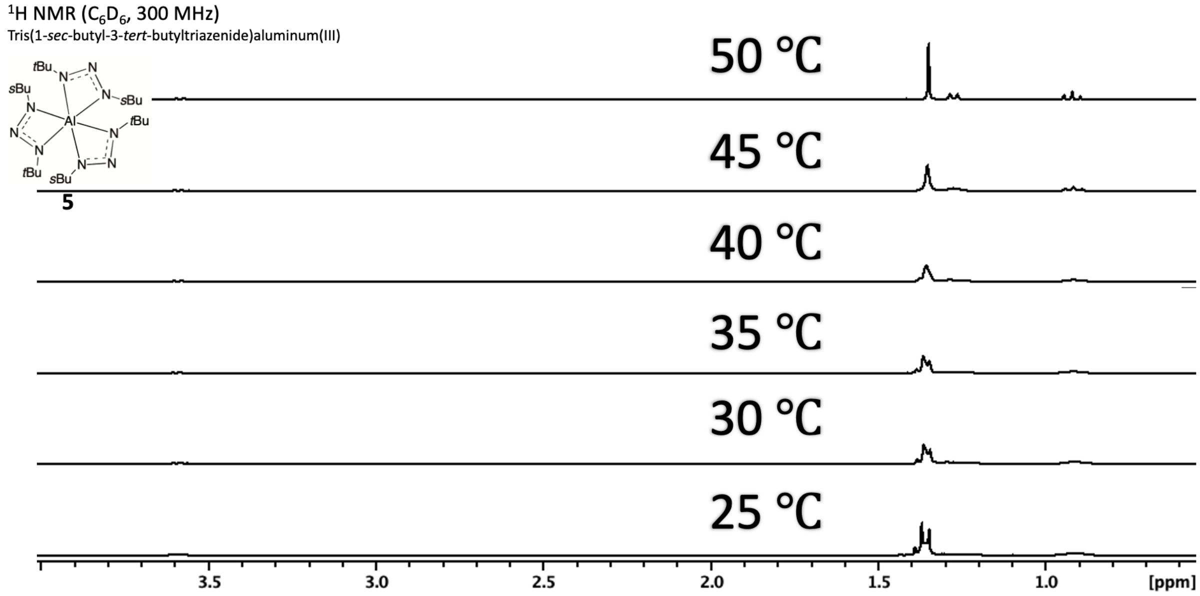

Figure S16: ^{1}H NMR VT spectrum of **5**.

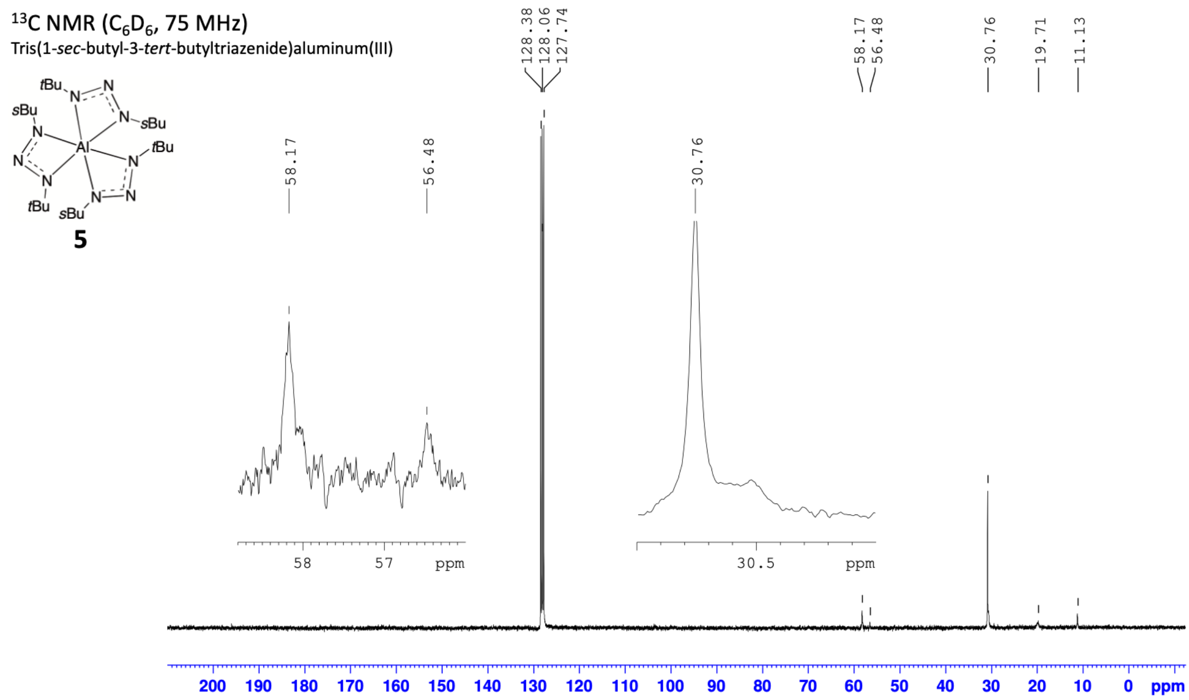

Figure S17: ^{13}C NMR spectrum of **5** at 50 °C.

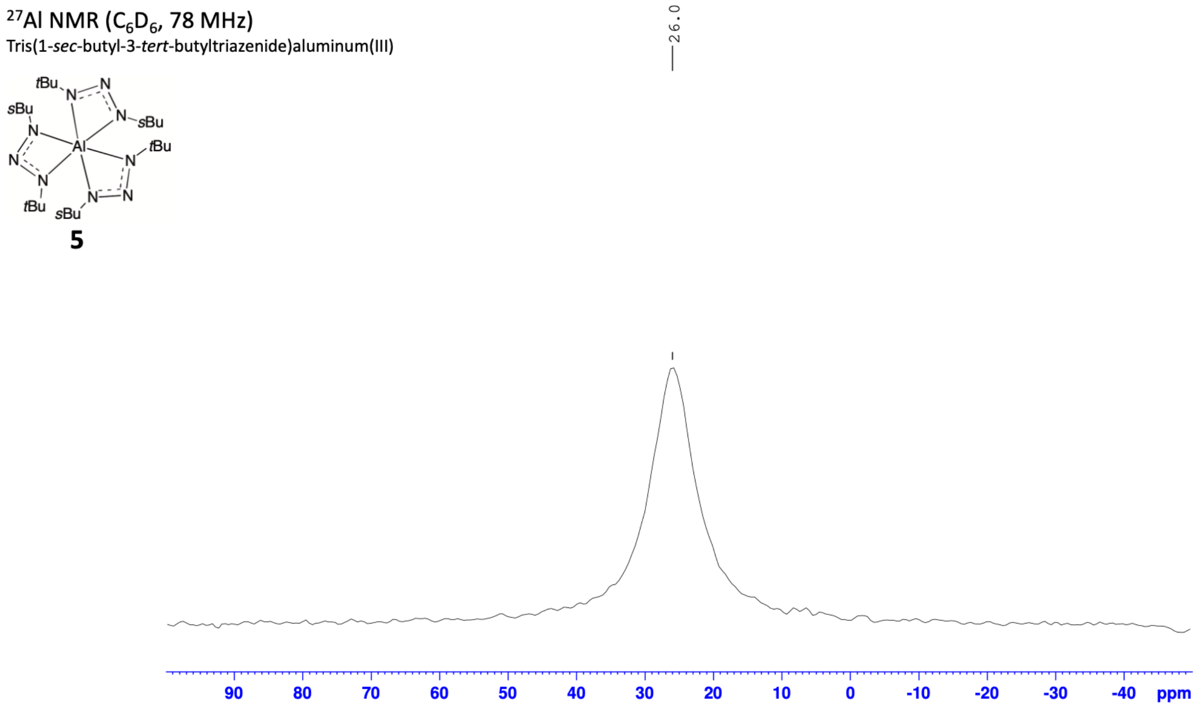

Figure S18: ^{27}Al NMR spectrum of **5**.

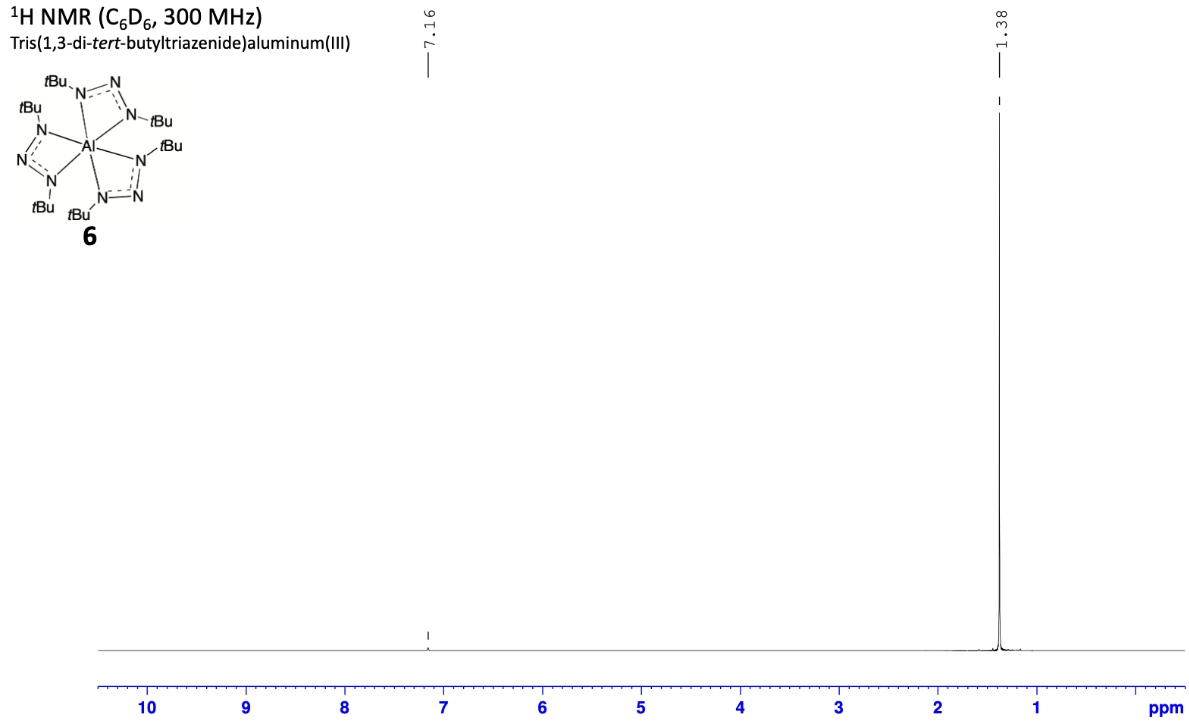

Figure S19: ^{1}H NMR spectrum of **6**.

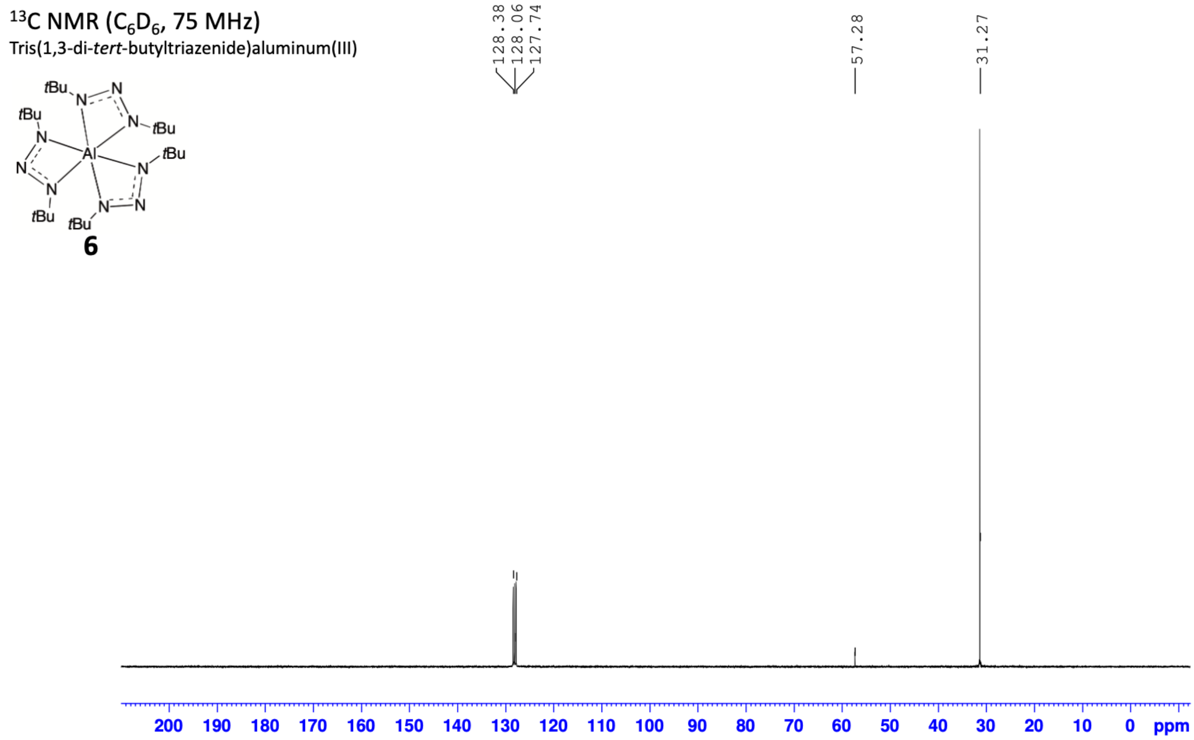

Figure S20: ^{13}C NMR spectrum of **6**.

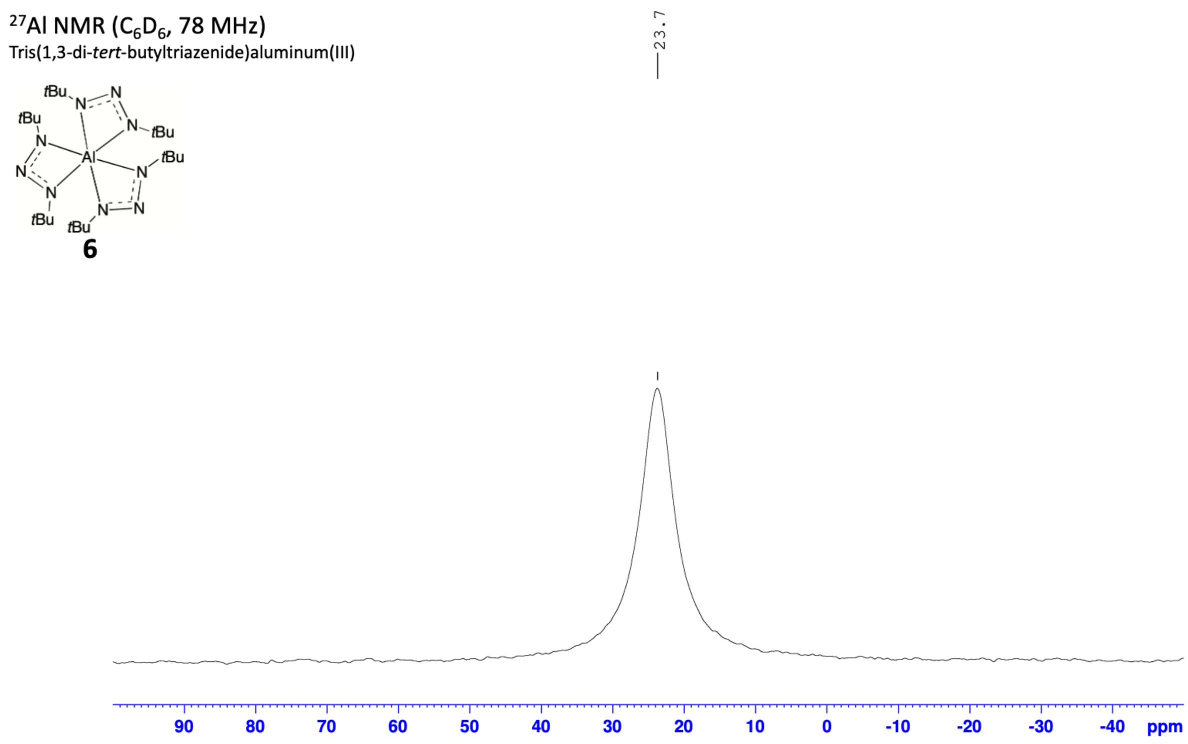

Figure S21: ^{27}Al NMR spectrum of **6**.

NMR decomposition study of **1**

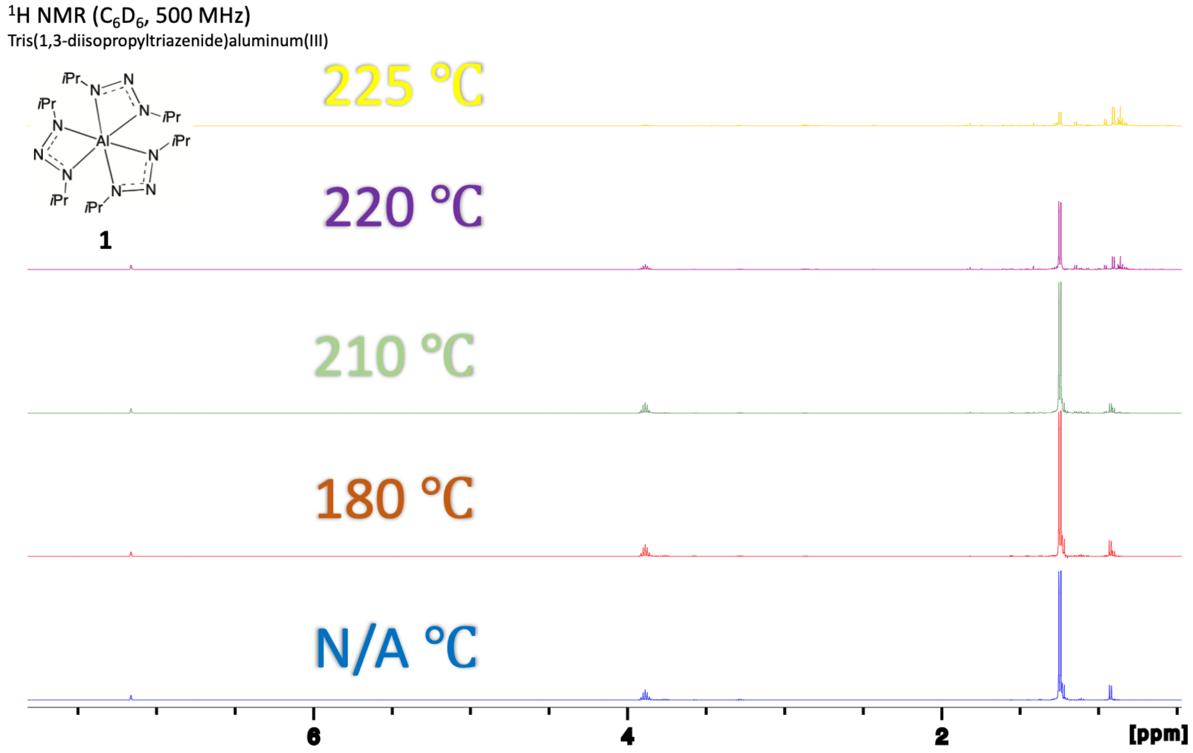

Figure S22: ^{1}H NMR in the 0–8 ppm range for NMR decomposition study of **1**. NMR experiments performed at 50 °C.

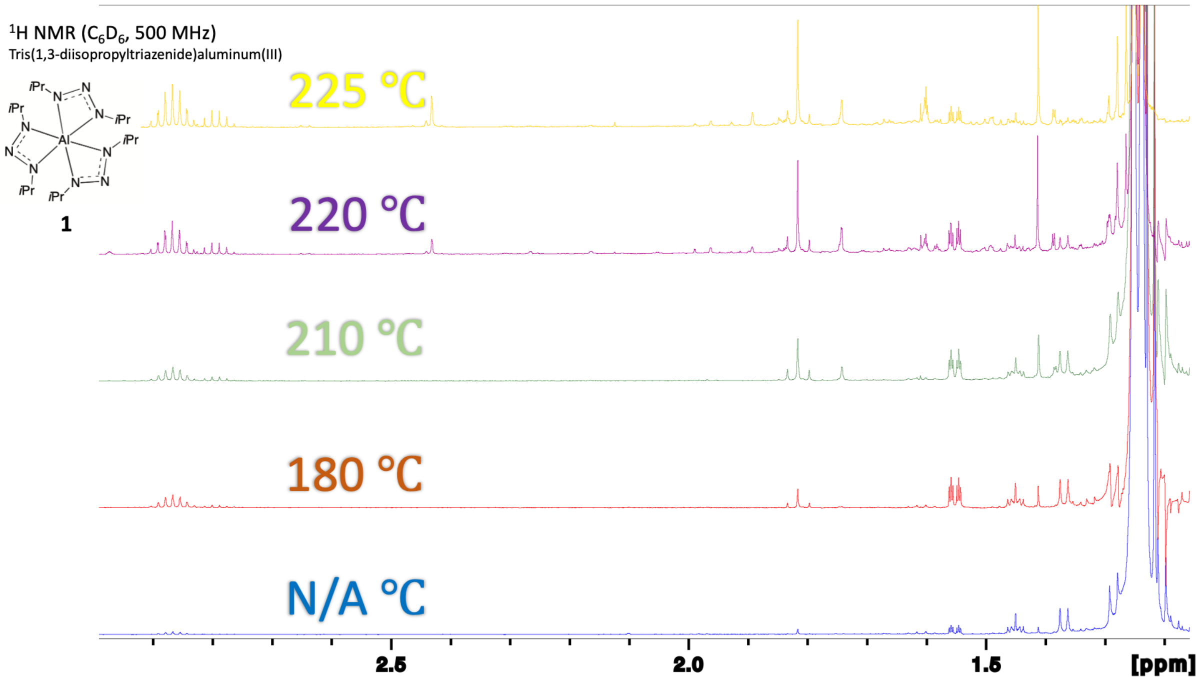

Figure S23: ^{1}H NMR in the 1.2–3.0 ppm range for NMR decomposition study of **1**. NMR experiments performed at 50 °C.

EI-MS for **1** and **6**

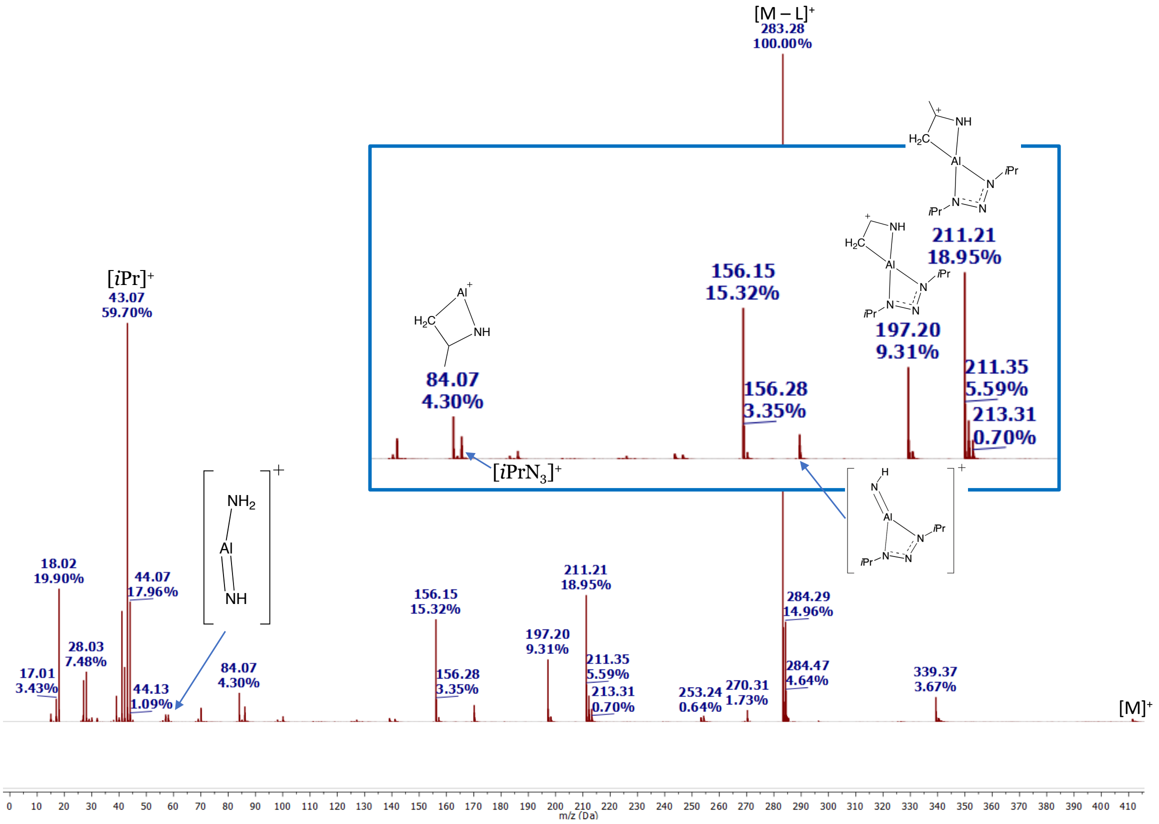

Figure S24: EI-MS data of **1** with potential fragments identified.

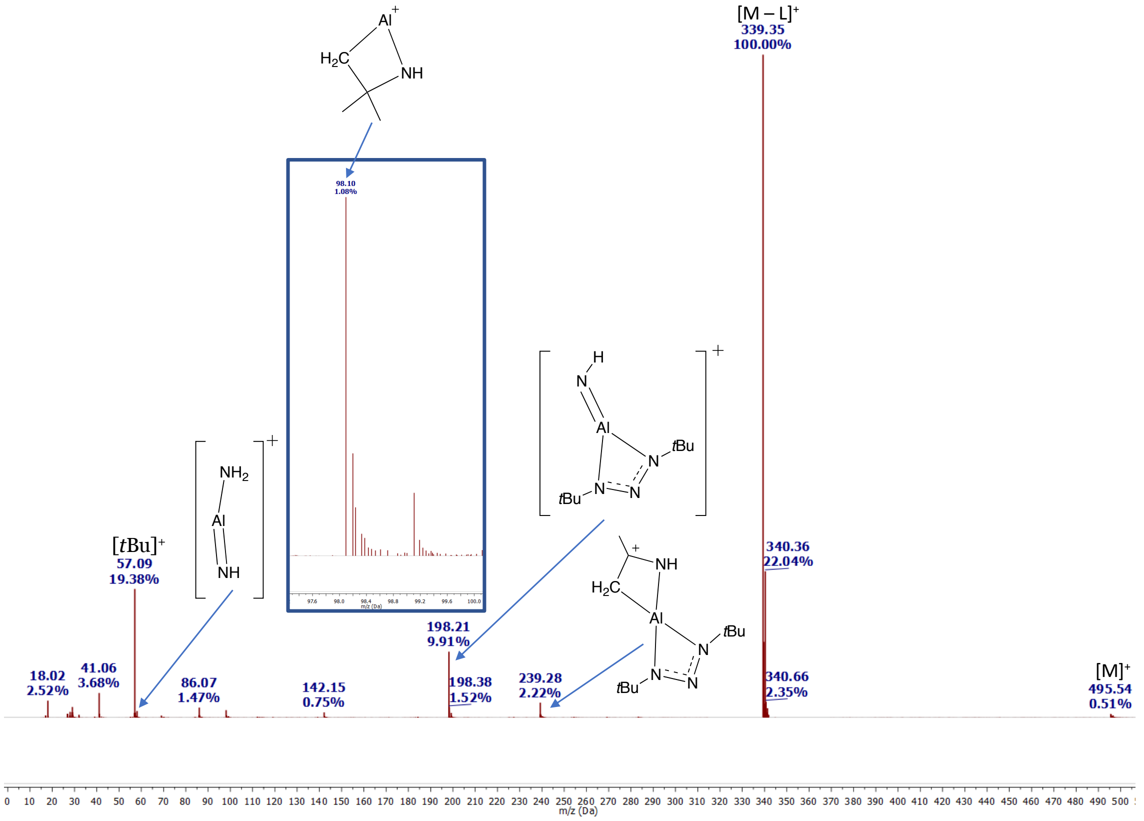

Figure S25: EI-MS data of **6** with potential fragments identified.

TGA curves and plots for calculating 1 Torr vapor pressure

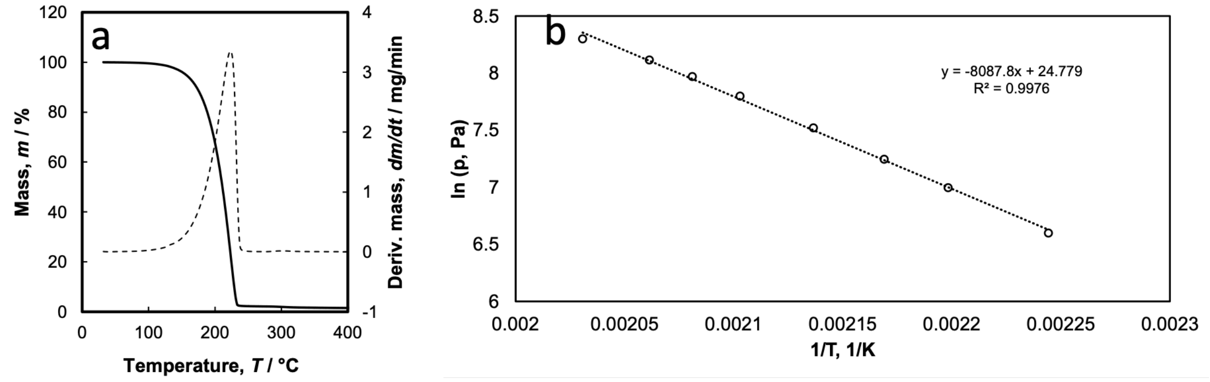

Figure S26: TGA curve (a) and Arrhenius plot for calculation of the 1 torr vapor pressure (b) for **1**.

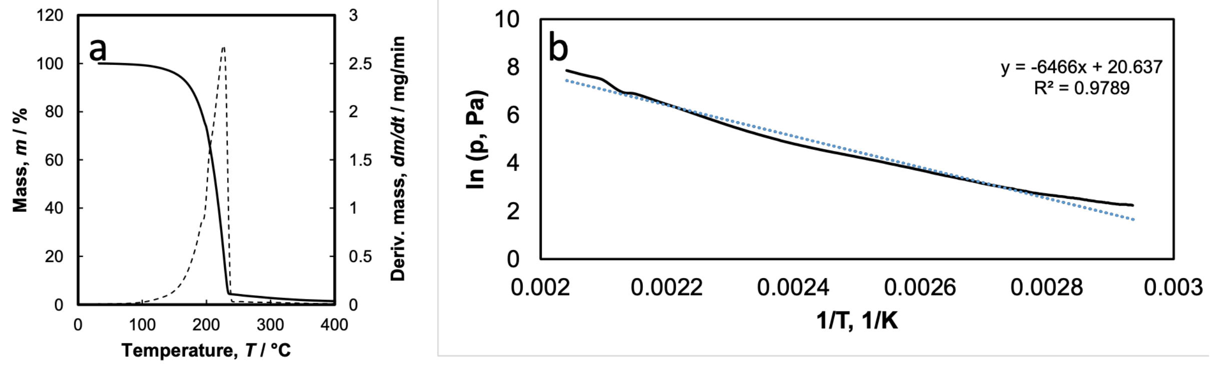

Figure S27: TGA curve (a) and Arrhenius plot for calculation of the 1 torr vapor pressure (b) for **2**.

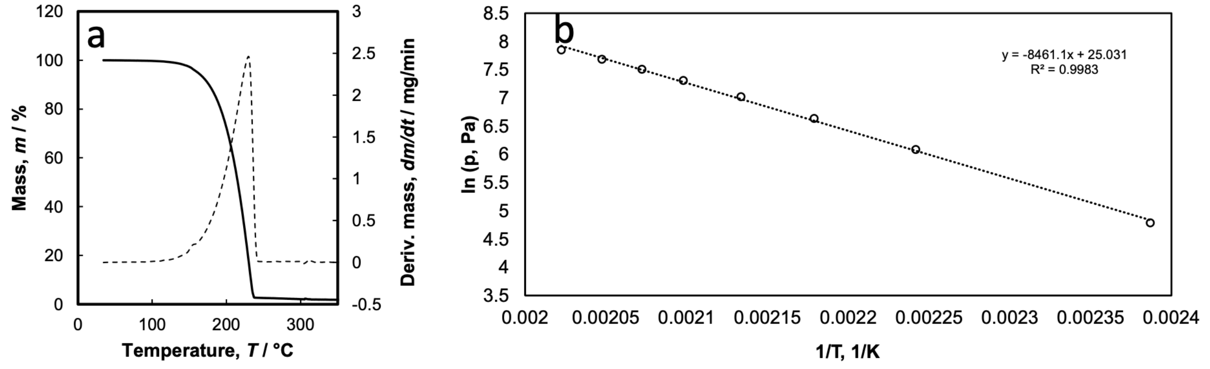

Figure S28: TGA curve (a) and Arrhenius plot for calculation of the 1 torr vapor pressure (b) for **3**.

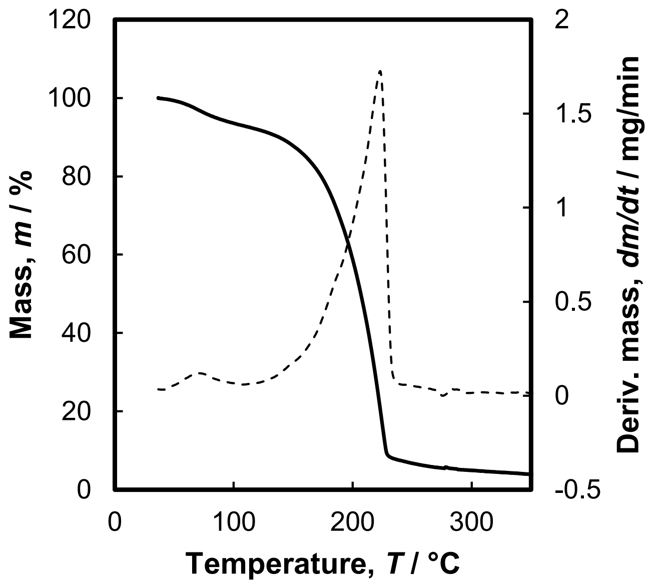

Figure S29: TGA curve of **4**.

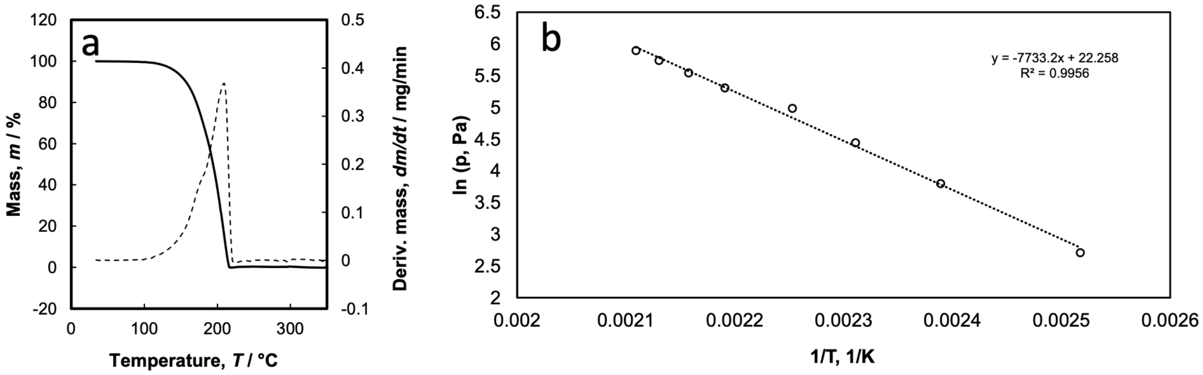

Figure S30: TGA curve (a) and Arrhenius plot for calculation of the 1 torr vapor pressure (b) for **5**.

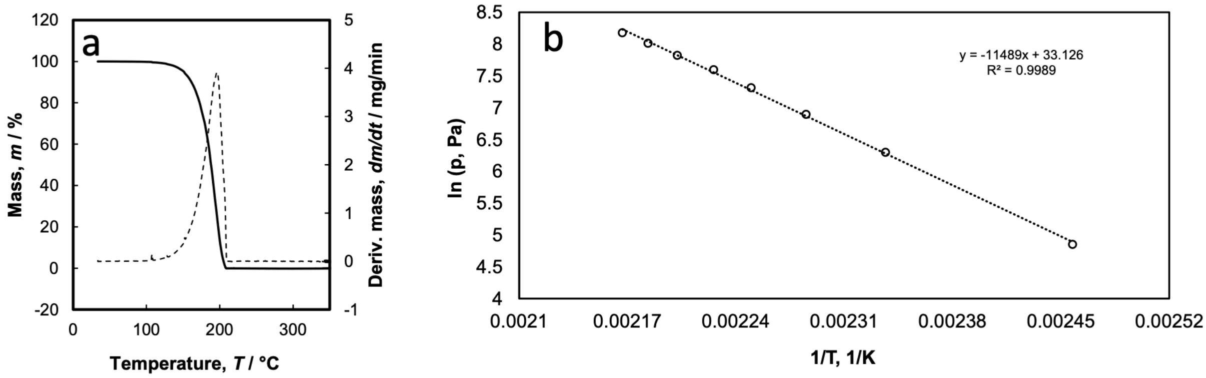

Figure S31: TGA curve (a) and Arrhenius plot for calculation of the 1 torr vapor pressure (b) for **6**.

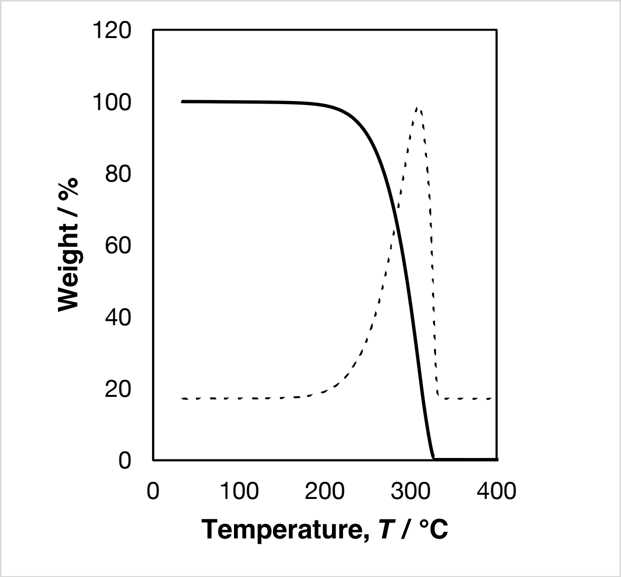

Figure S32: TGA curve of amidinate analog of compound **1**.

DSC data

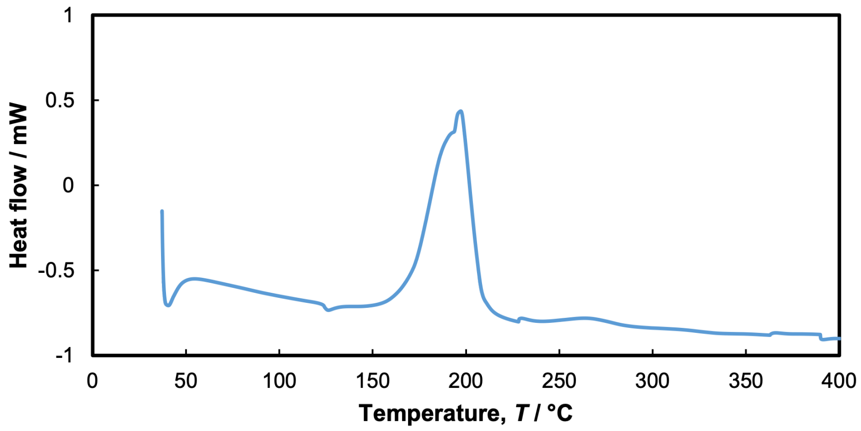

Figure S33: DSC of **1**.

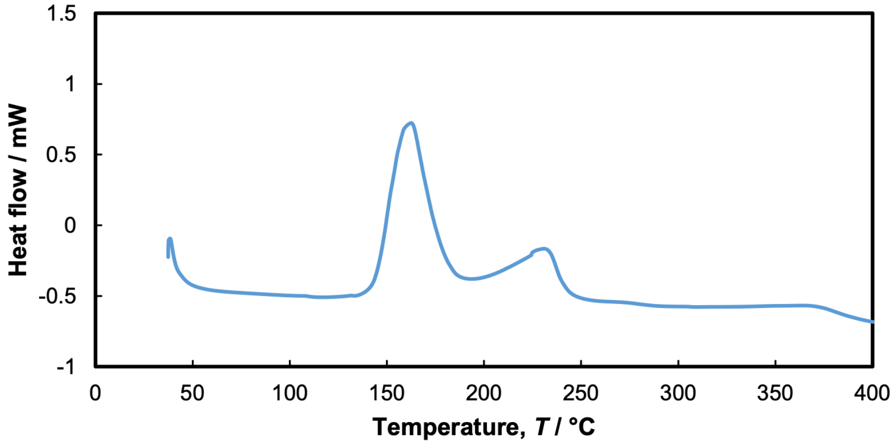

Figure S34: DSC of **2**.

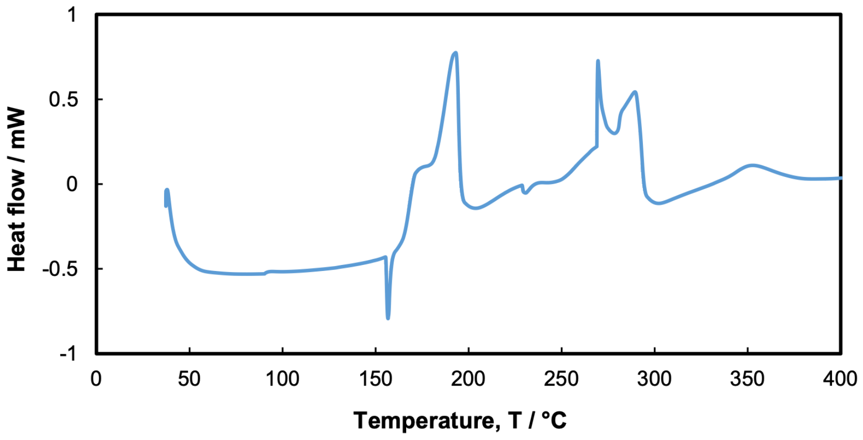

Figure S35: DSC of **3**.

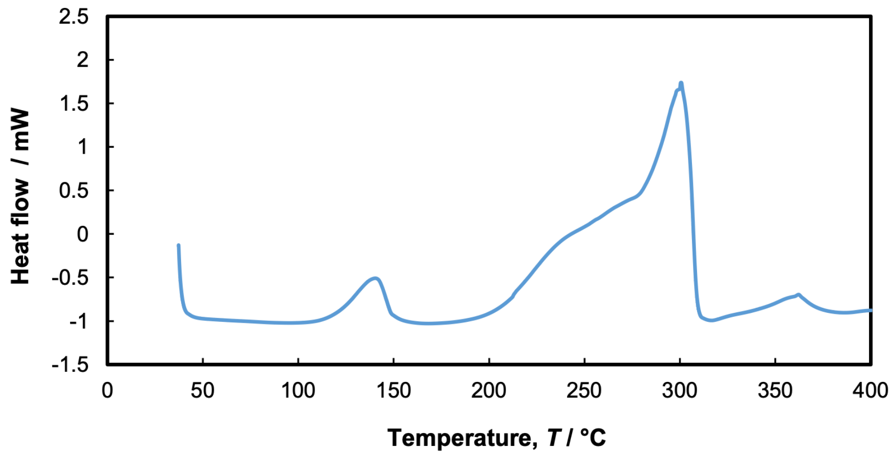

Figure S36: DSC of **4**.

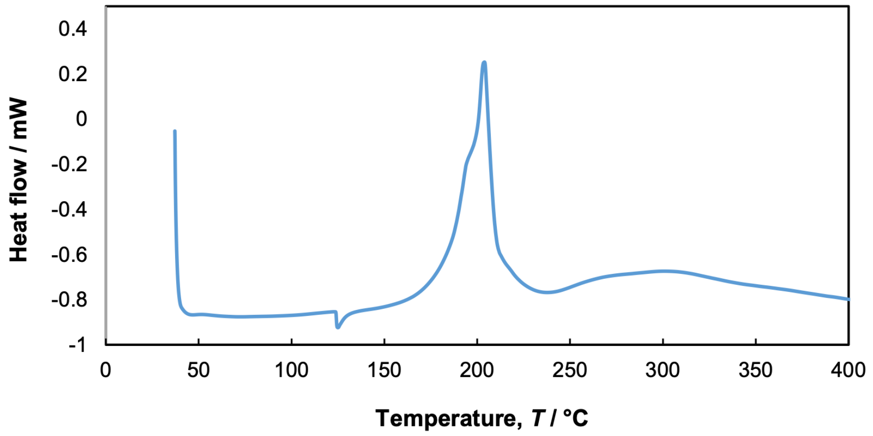

Figure S37: DSC of **5**.

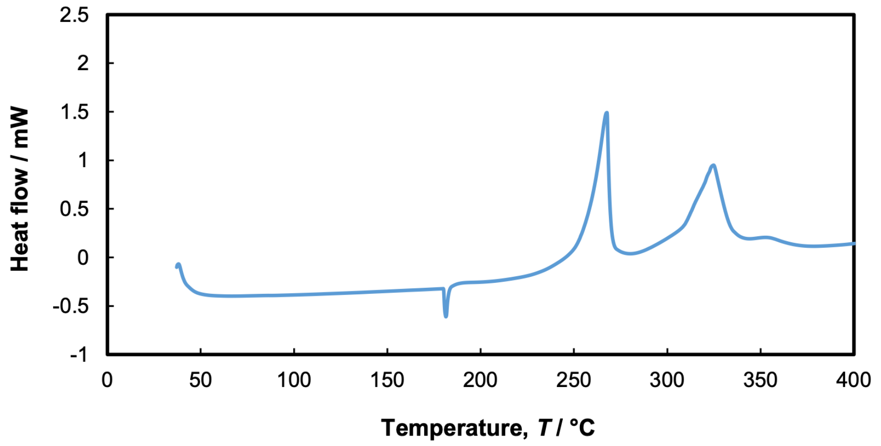

Figure S38: DSC of **6**.

Computational Details

Selected bond lengths for transition state and intermediate structures for the gas phase decomposition pathway of **1**

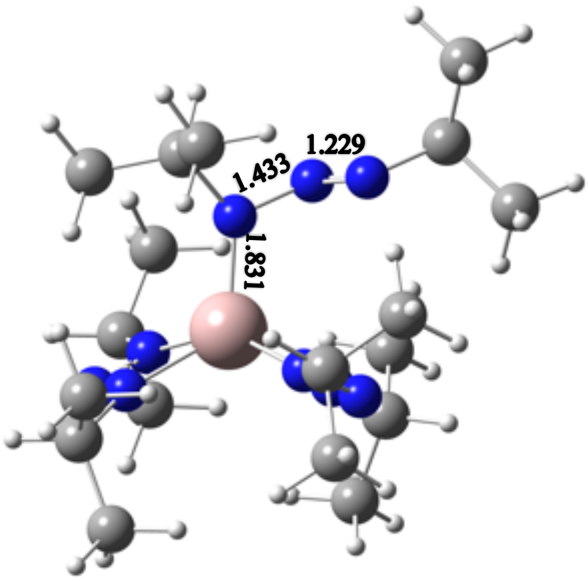

Figure S39: **TS-1**, selected bond lengths in Å.

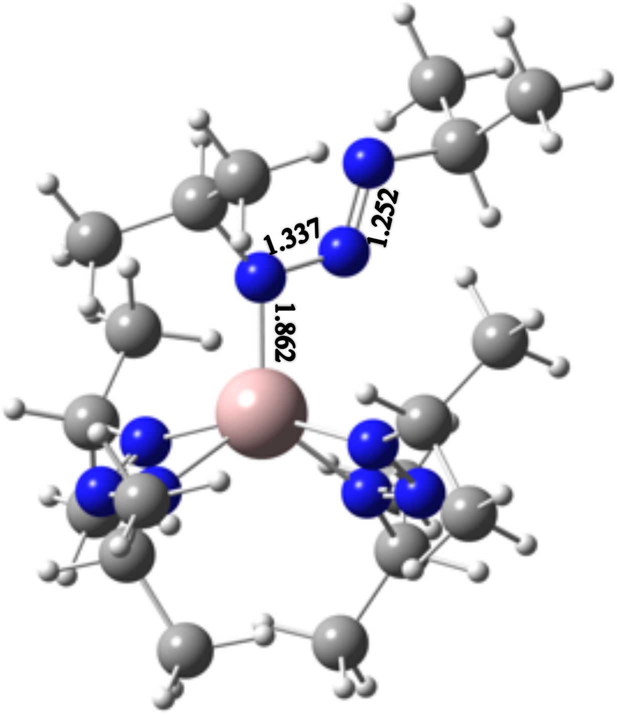

Figure S40: **I-1**, selected bond lengths in Å.

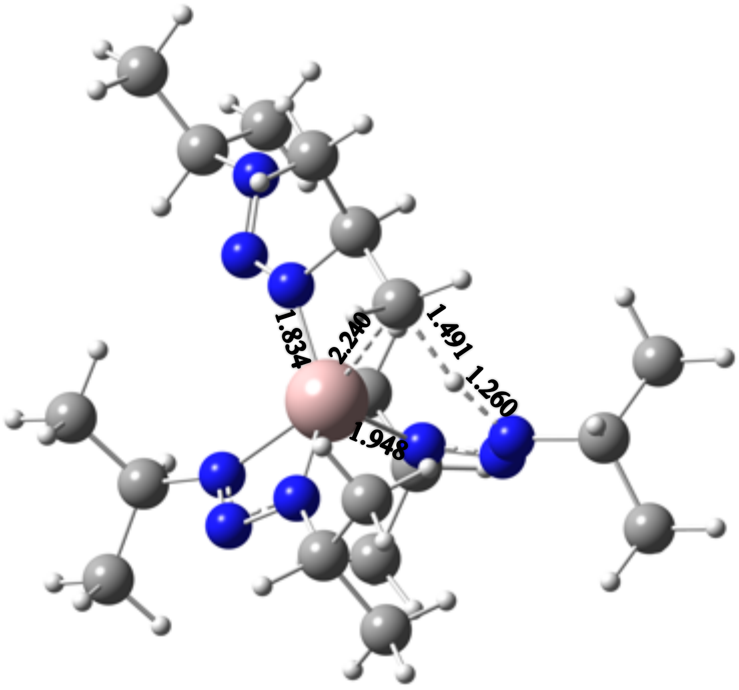

Figure S41: **TS-2**, selected bond lengths in Å.

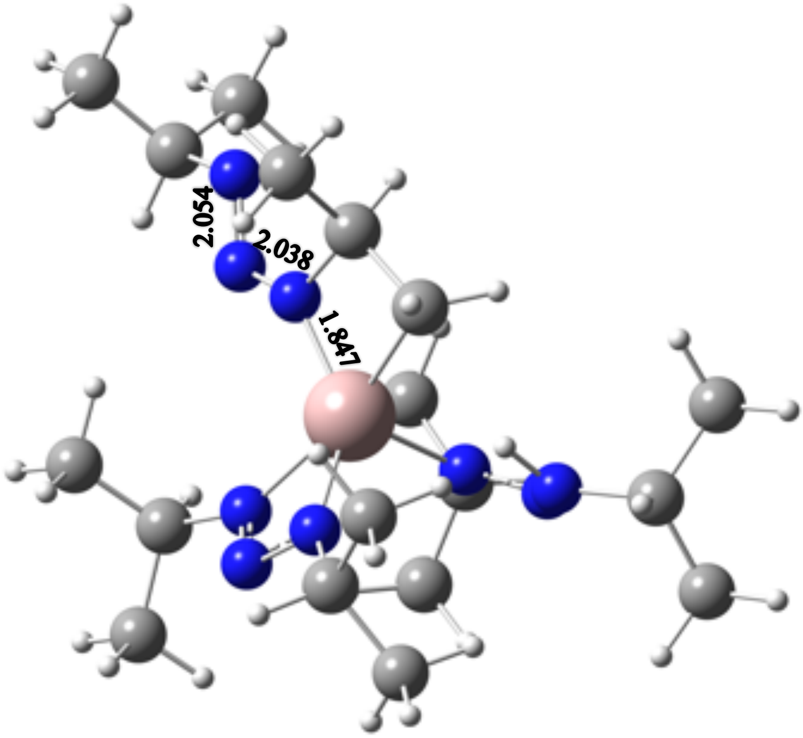

Figure S42: **I-2A**, selected bond lengths in Å.

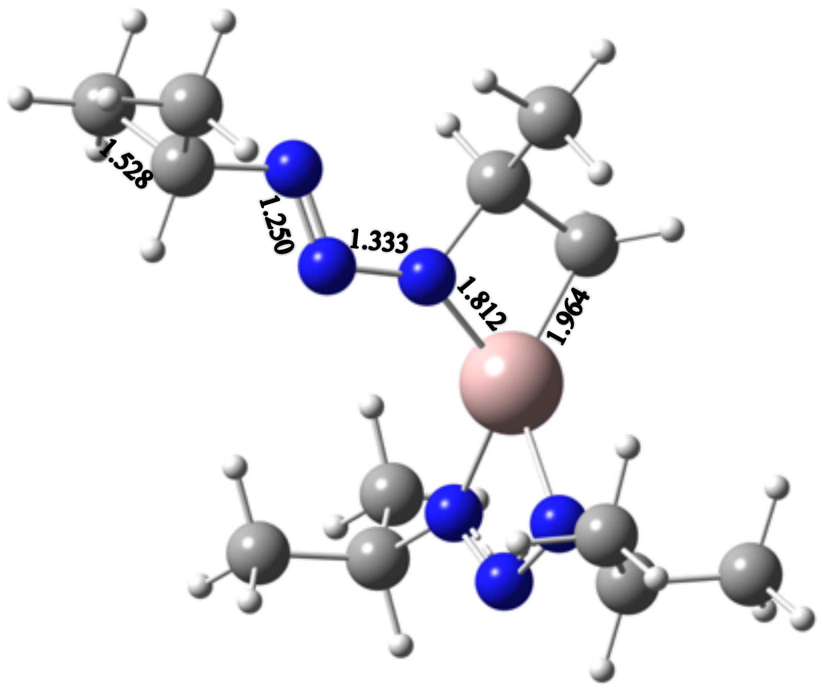

Figure S43: **I-2**, selected bond lengths in Å.

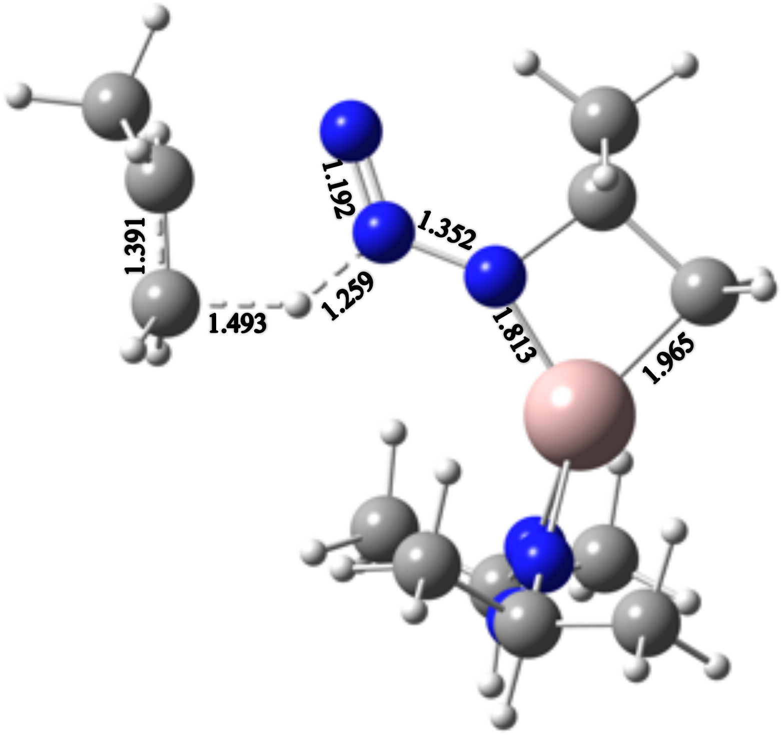

Figure S44: **TS-3**, selected bond lengths in Å.

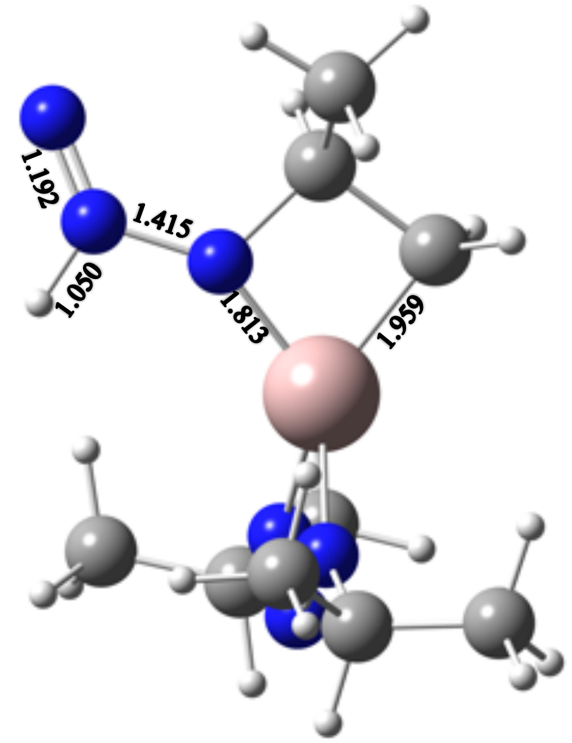

Figure S45: **I-3**, selected bond lengths in Å.

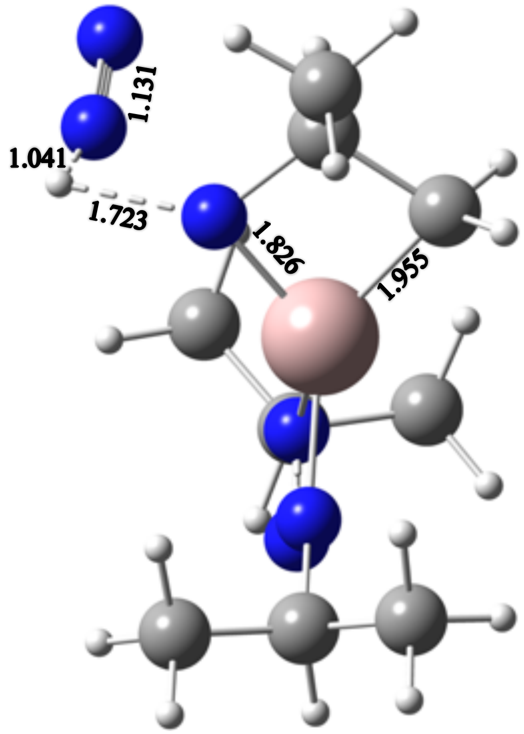

Figure S46: **TS-4**, selected bond lengths in Å.

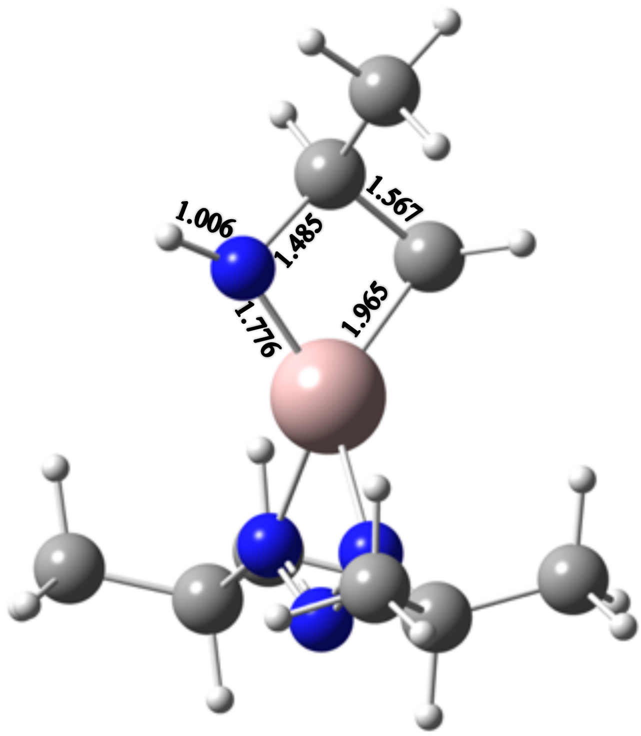

Figure S47: **I-4**, selected bond lengths in Å.

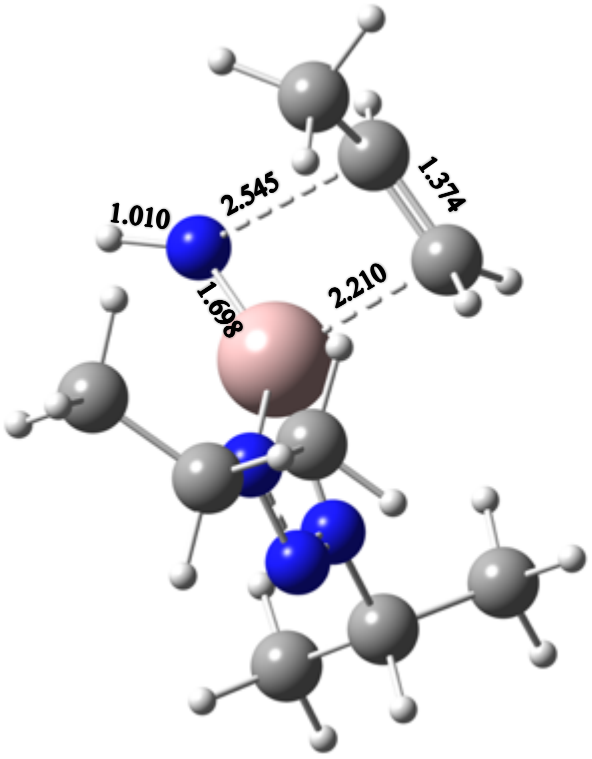

Figure S48: **TS-5**, selected bond lengths in Å.

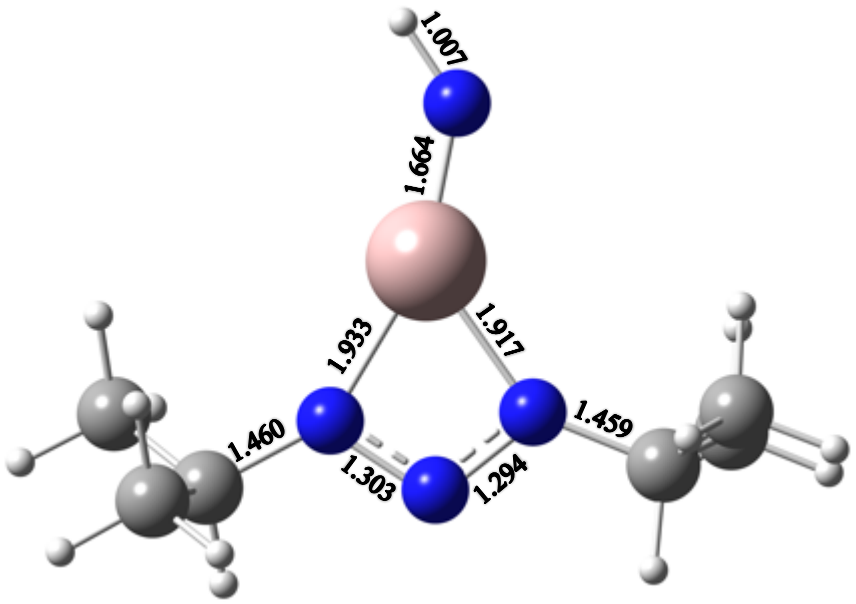

Figure S49: **I-5**, selected bond lengths in Å.

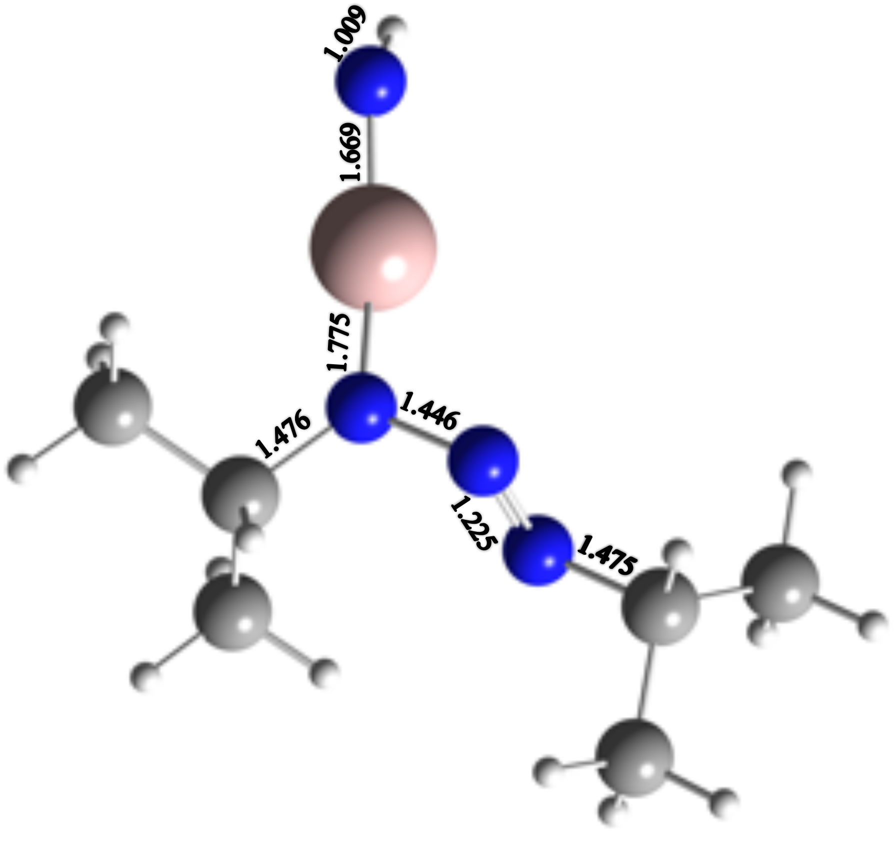

Figure S50: **TS-6**, selected bond lengths in Å.

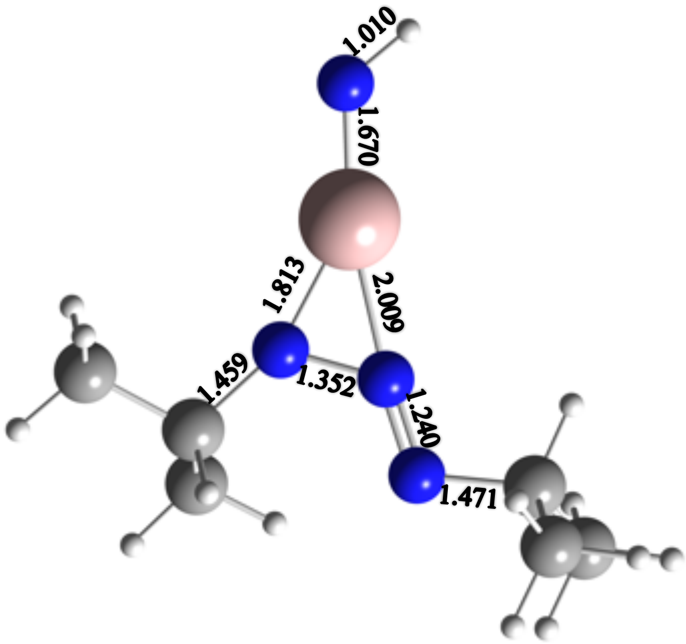

Figure S51: **I-6**, selected bond lengths in Å.

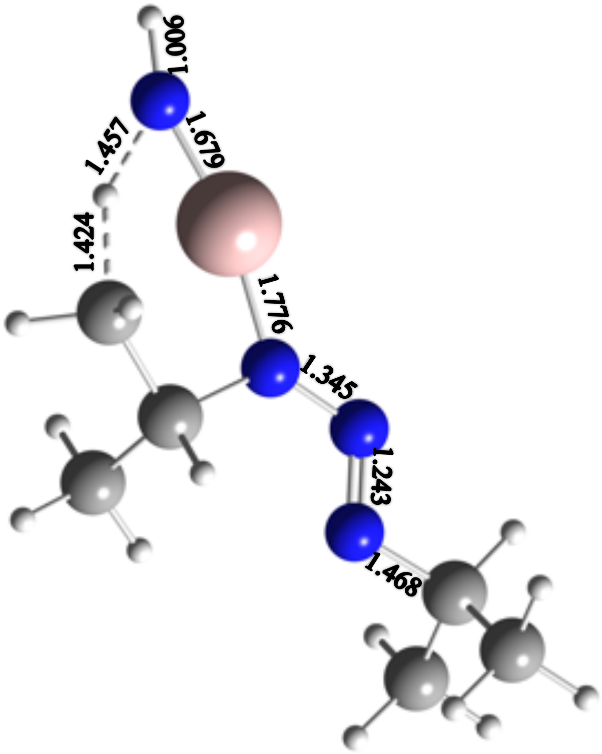

Figure S52: **TS-7**, selected bond lengths in Å.

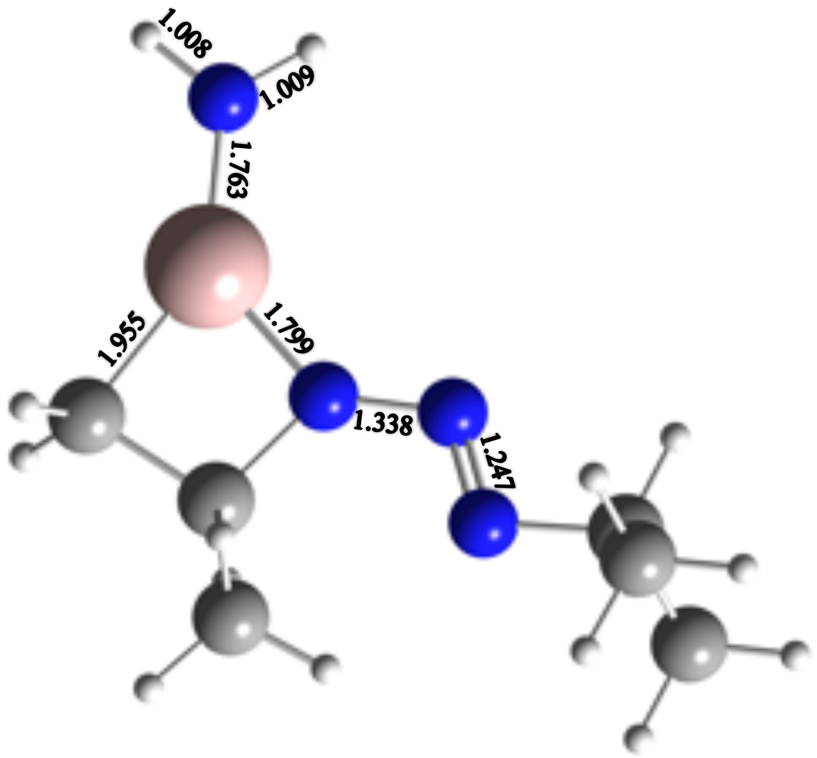

Figure S53: **I-7**, selected bond lengths in Å.

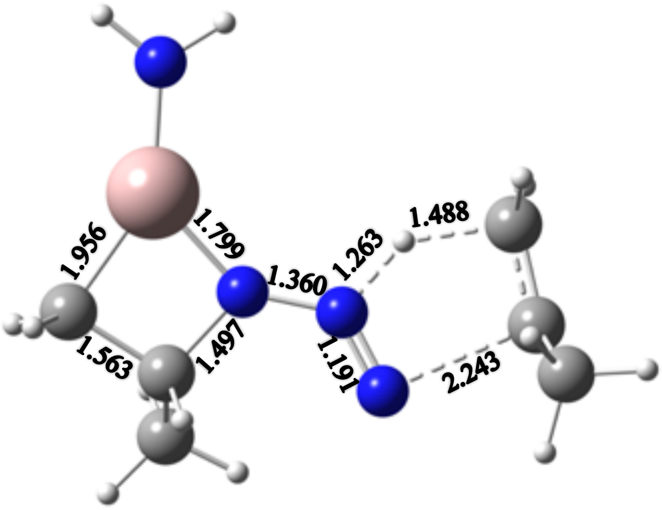

Figure S54: **TS-8**, selected bond lengths in Å.

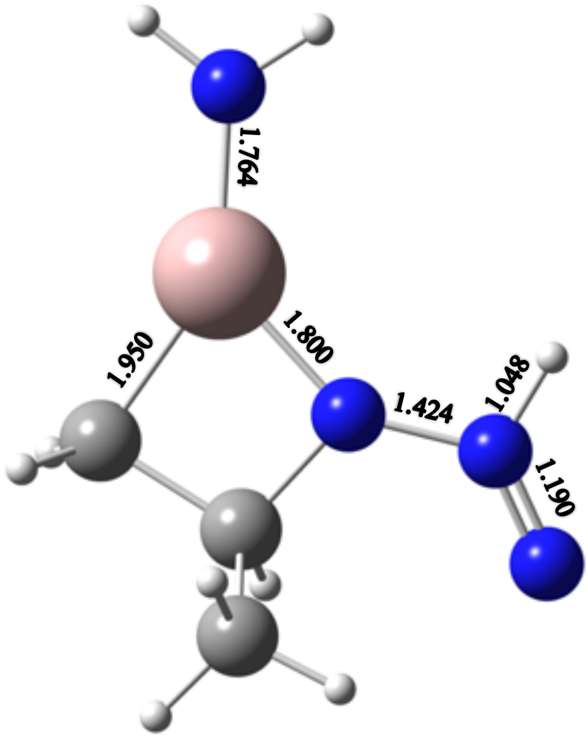

Figure S55: **I-8**, selected bond lengths in Å.

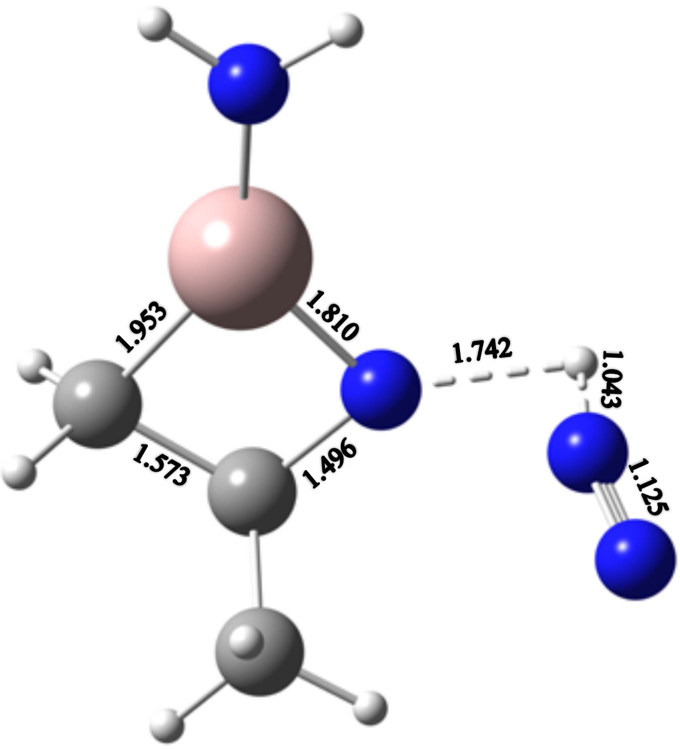

Figure S56: **TS-9**, selected bond lengths in Å.

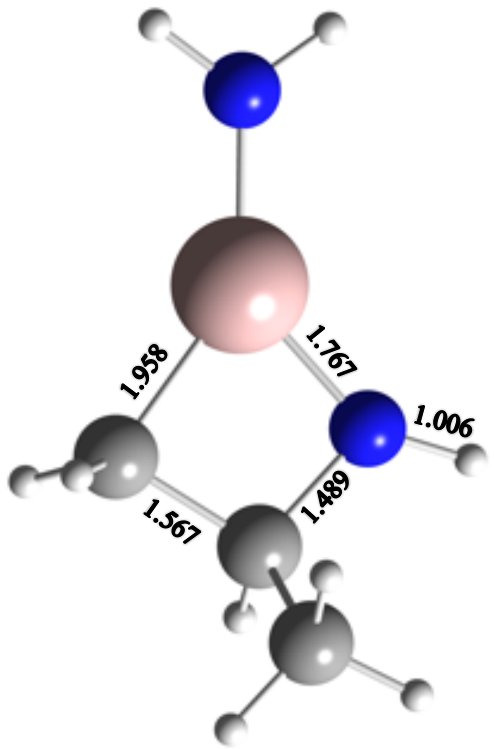

Figure S57: **I-9**, selected bond lengths in Å.

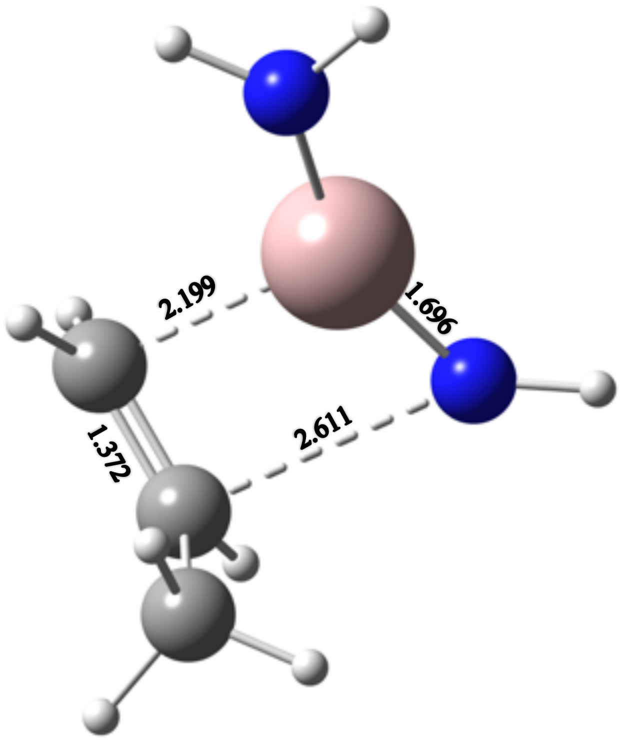

Figure S58: **TS-10**, selected bond lengths in Å.

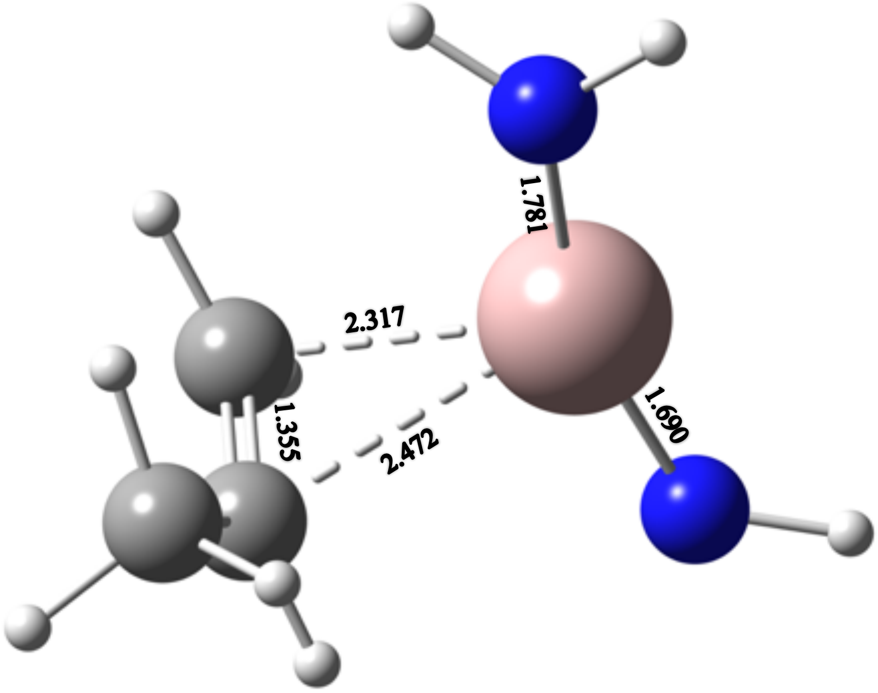

Figure S59: **I-10A**, selected bond lengths in Å.

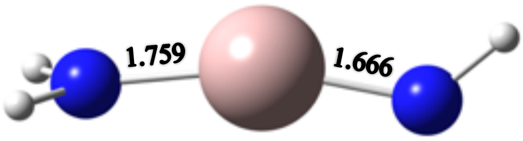

Figure S60: **I-10**, selected bond lengths in Å.

Schemes for decomposition of **1**

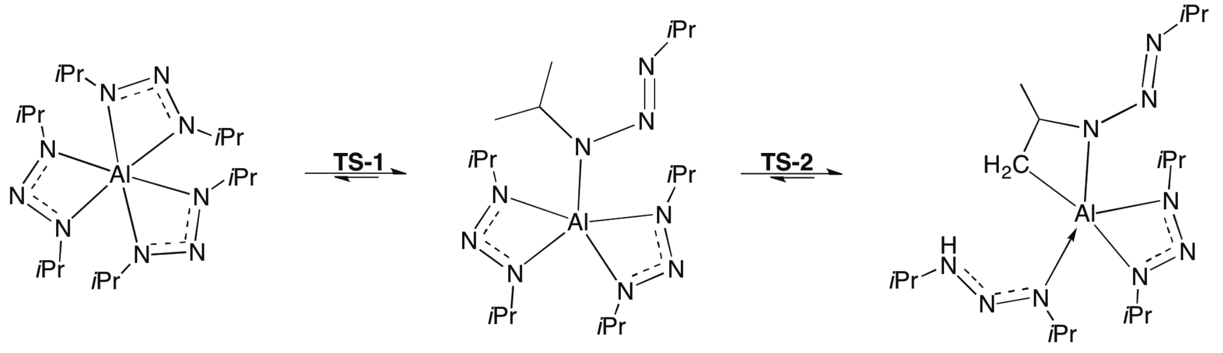

1 **I-1** **I-2A**

Scheme S1: The decomposition is initiated by one ligand de-chelating, transforming **1** into **I-1**. Next, a methyl proton migrates from the iso-propyl on the coordinated nitrogen to a neighboring ligand, resulting in the adduct structure, **I-2A**.

I-2 **I-3**

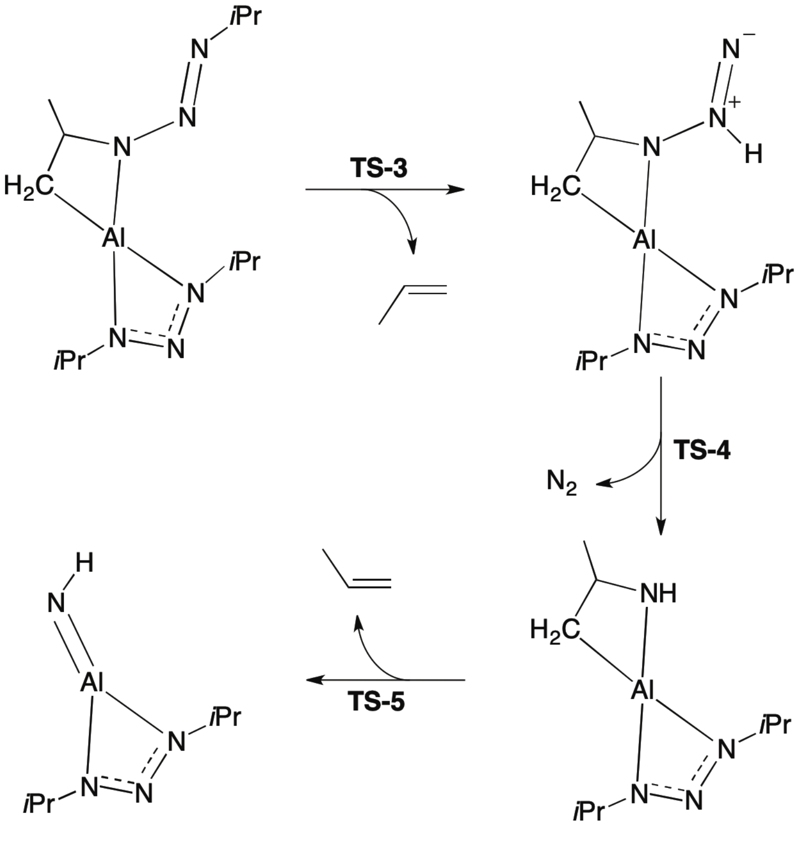

I-5 **I-4**

Scheme S2: Decomposition of the dianionic C,N-coordinated triazenide ligand in three steps. Part of the triazenide ligand is released as propene, dinitrogen and propene during **TS-3**, **TS-4** and **TS-5** respectively.

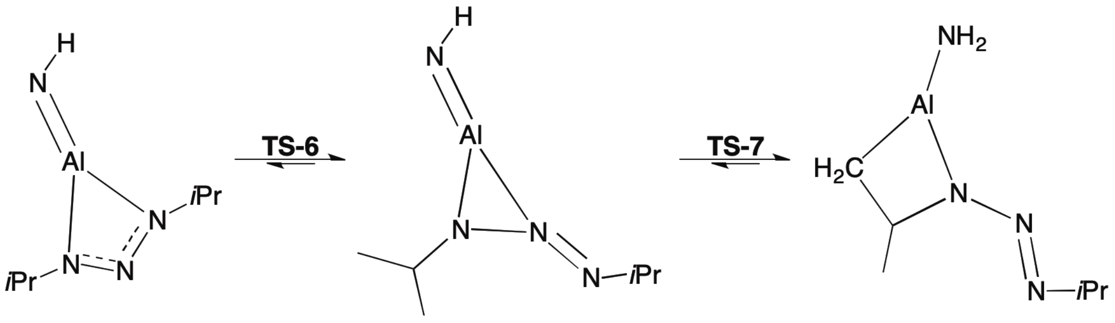

I-5 I-6 I-7

Scheme S3: Decomposition of the last remaining triazenide ligand. Passing **TS-6** causes dechelation by rotating 180° along a N–N bond, analogous to **TS-1**. The binding mode of the triazenide ligand in **I-6** is, however, a bit different than in **I-1**. In **I-6**, the triazenide binds to the metal center with two neighboring nitrogen atoms, forming a three-membered ring. While approaching **TS-7**, the triazenide ligand become monodentate without passing a transition state. Analogous to **TS-2**, an inter-ligand proton migration occurs in **TS-7**. A methyl proton migrates from the isopropyl on the coordinated nitrogen to the neighboring imido ligand (analogous to **TS-2**).

I-7 I-8

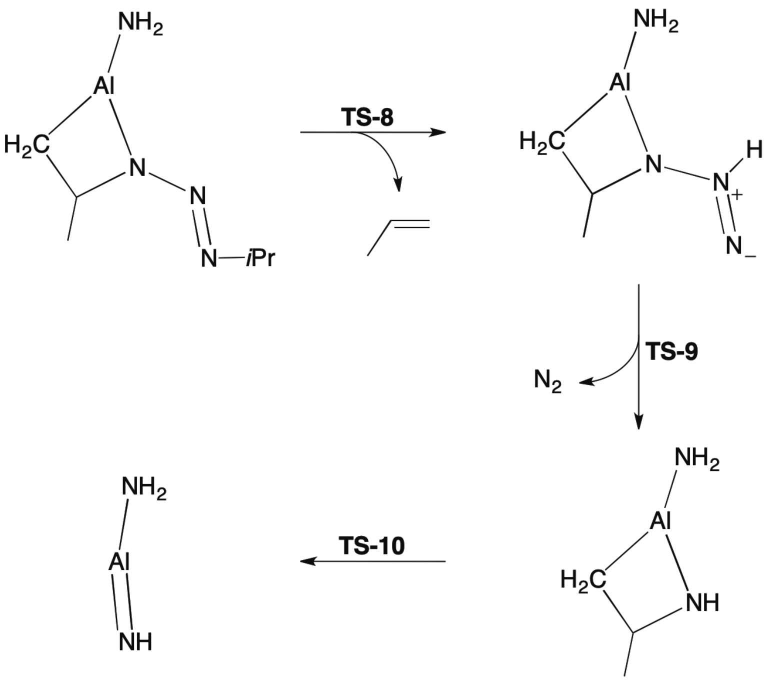

I-10 I-9

Scheme S4: A three-step decomposition of the second dianionic C,N-coordinated triazenide ligand. The steps, **I-7** to **I-10**, transforming the triazenide ligand into an imido ligand are analogous to the decomposition of the first ligand (**I-I** to **I-5**)

Thermochemical data from vibrational analysis for decomposition of **1** and **6**

Table S1: Thermochemical and electronic data from energy calculation and vibrational analysis for **1** and associated transition states and intermediates (displayed in first row, first column). Each column, 2–11, have thermochemical data (in Hartree) calculated at a specific temperature and pressure (displayed in the first element of each column). For the first column, E is the electronic energy, E+ZPE is the sum of electronic and zero-point energy, E+E_Thermal is the sum of electronic and thermal energy, H is the enthalpy and G is the Gibbs free energy.

**1**	**273.15 K, 10 hPa**	**298.15 K, 10 hPa**	**323.15 K, 10 hPa**	**373.15 K, 10 hPa**	**423.15 K, 10 hPa**	**523.15 K, 10 hPa**	**773.15 K, 10 hPa**	**373.15 K, 1 hPa**	**373.15 K, 100 hPa**	**373.15 K, 1000 hPa**
E	-1447.022572									
E+ZPE	-1446.406921	-1446.406921	-1446.406921	-1446.406921	-1446.406921	-1446.406921	-1446.406921	-1446.406921	-1446.406921	-1446.406921
E+E_Thermal	-1446.376057	-1446.370927	-1446.365448	-1446.353446	-1446.340078	-1446.309527	-1446.214985	-1446.353446	-1446.353446	-1446.353446
H	0.647380	0.652589	0.658147	0.670309	0.683835	0.714702	0.810036	0.670309	0.670309	0.670309
G	0.553232	0.544386	0.535089	0.515171	0.493530	0.445262	0.297576	0.512449	0.517892	0.520613
**TS-1**	**273.15 K, 10 hPa**	**298.15 K, 10 hPa**	**323.15 K, 10 hPa**	**373.15 K, 10 hPa**	**423.15 K, 10 hPa**	**523.15 K, 10 hPa**	**773.15 K, 10 hPa**	**373.15 K, 1 hPa**	**373.15 K, 100 hPa**	**373.15 K, 1000 hPa**
E	-1446.966948									
E+ZPE	-1446.354191	-1446.354191	-1446.354191	-1446.354191	-1446.354191	-1446.354191	-1446.354191	-1446.354191	-1446.354191	-1446.354191
E+E_Thermal	-1446.323011	-1446.317900	-1446.312444	-1446.300496	-1446.287192	-1446.256796	-1446.162757	-1446.300496	-1446.300496	-1446.300496
H	0.644801	0.649991	0.655527	0.667633	0.681095	0.711808	0.806639	0.667633	0.667633	0.667633
G	0.548989	0.539992	0.530545	0.510333	0.488407	0.439592	0.290678	0.507611	0.513054	0.515775
**I-1**	**273.15 K, 10 hPa**	**298.15 K, 10 hPa**	**323.15 K, 10 hPa**	**373.15 K, 10 hPa**	**423.15 K, 10 hPa**	**523.15 K, 10 hPa**	**773.15 K, 10 hPa**	**373.15 K, 1 hPa**	**373.15 K, 100 hPa**	**373.15 K, 1000 hPa**
E	-1446.997198									
E+ZPE	-1446.383365	-1446.383365	-1446.383365	-1446.383365	-1446.383365	-1446.383365	-1446.383365	-1446.383365	-1446.383365	-1446.383365
E+E_Thermal	-1446.351707	-1446.346548	-1446.341044	-1446.328994	-1446.315585	-1446.284963	-1446.190285	-1446.328994	-1446.328994	-1446.328994
H	0.646357	0.651594	0.657178	0.669386	0.682953	0.713892	0.809362	0.669386	0.669386	0.669386
G	0.548221	0.539009	0.529344	0.508683	0.486294	0.436516	0.285007	0.505961	0.511404	0.514125
**TS-2**	**273.15 K, 10 hPa**	**298.15 K, 10 hPa**	**323.15 K, 10 hPa**	**373.15 K, 10 hPa**	**423.15 K, 10 hPa**	**523.15 K, 10 hPa**	**773.15 K, 10 hPa**	**373.15 K, 1 hPa**	**373.15 K, 100 hPa**	**373.15 K, 1000 hPa**
E	-1446.926176									

E+ZPE	-1446.316406	-1446.316406	-1446.316406	-1446.316406	-1446.316406	-1446.316406	-1446.316406	-1446.316406	-1446.316406	-1446.316406
E+E_Thermal	-1446.286021	-1446.280952	-1446.275529	-1446.263624	-1446.250338	-1446.219913	-1446.125591	-1446.263624	-1446.263624	-1446.263624
H	0.641020	0.646168	0.651670	0.663734	0.677179	0.707920	0.803034	0.663734	0.663734	0.663734
G	0.546956	0.538120	0.528838	0.508963	0.487376	0.439242	0.291968	0.506241	0.511684	0.514405
**I-2A**	**273.15 K, 10 hPa**	**298.15 K, 10 hPa**	**323.15 K, 10 hPa**	**373.15 K, 10 hPa**	**423.15 K, 10 hPa**	**523.15 K, 10 hPa**	**773.15 K, 10 hPa**	**373.15 K, 1 hPa**	**373.15 K, 100 hPa**	**373.15 K, 1000 hPa**
E	-1446.938214									
E+ZPE	-1446.324062	-1446.324062	-1446.324062	-1446.324062	-1446.324062	-1446.324062	-1446.324062	-1446.324062	-1446.324062	-1446.324062
E+E_Thermal	-1446.293008	-1446.287871	-1446.282380	-1446.270340	-1446.256922	-1446.226253	-1446.131434	-1446.270340	-1446.270340	-1446.270340
H	0.646071	0.651287	0.656857	0.669055	0.682632	0.713617	0.809228	0.669055	0.669055	0.669055
G	0.549755	0.540711	0.531214	0.510893	0.488844	0.439740	0.289873	0.508171	0.513614	0.516335
**I-2**	**273.15 K, 10 hPa**	**298.15 K, 10 hPa**	**323.15 K, 10 hPa**	**373.15 K, 10 hPa**	**423.15 K, 10 hPa**	**523.15 K, 10 hPa**	**773.15 K, 10 hPa**	**373.15 K, 1 hPa**	**373.15 K, 100 hPa**	**373.15 K, 1000 hPa**
E	-1044.848428									
E+ZPE	-1044.452017	-1044.452017	-1044.452017	-1044.452017	-1044.452017	-1044.452017	-1044.452017	-1044.452017	-1044.452017	-1044.452017
E+E_Thermal	-1044.431293	-1044.427923	-1044.424319	-1044.416411	-1044.407594	-1044.387439	-1044.325182	-1044.416411	-1044.416411	-1044.416411
H	0.418001	0.421449	0.425132	0.433198	0.442174	0.462646	0.525695	0.433198	0.433198	0.433198
G	0.341833	0.334711	0.327289	0.311568	0.294704	0.257668	0.147186	0.308846	0.314289	0.317010
**TS-3**	**273.15 K, 10 hPa**	**298.15 K, 10 hPa**	**323.15 K, 10 hPa**	**373.15 K, 10 hPa**	**423.15 K, 10 hPa**	**523.15 K, 10 hPa**	**773.15 K, 10 hPa**	**373.15 K, 1 hPa**	**373.15 K, 100 hPa**	**373.15 K, 1000 hPa**
E	-1044.761312									
E+ZPE	-1044.371959	-1044.371959	-1044.371959	-1044.371959	-1044.371959	-1044.371959	-1044.371959	-1044.371959	-1044.371959	-1044.371959
E+E_Thermal	-1044.351183	-1044.347794	-1044.344169	-1044.336217	-1044.327356	-1044.307125	-1044.244800	-1044.336217	-1044.336217	-1044.336217
H	0.410994	0.414462	0.418166	0.426276	0.435296	0.455843	0.518960	0.426276	0.426276	0.426276
G	0.335004	0.327897	0.320489	0.304790	0.287942	0.250925	0.140451	0.302068	0.307511	0.310232
**I-3**	**273.15 K, 10 hPa**	**298.15 K, 10 hPa**	**323.15 K, 10 hPa**	**373.15 K, 10 hPa**	**423.15 K, 10 hPa**	**523.15 K, 10 hPa**	**773.15 K, 10 hPa**	**373.15 K, 1 hPa**	**373.15 K, 100 hPa**	**373.15 K, 1000 hPa**
E	-926.8066094									
E+ZPE	-926.495011	-926.495011	-926.495011	-926.495011	-926.495011	-926.495011	-926.495011	-926.495011	-926.495011	-926.495011
E+E_Thermal	-926.477755	-926.474982	-926.472021	-926.465537	-926.458327	-926.441904	-926.391453	-926.465537	-926.465537	-926.465537

H	0.329720	0.332572	0.335612	0.342255	0.349623	0.366362	0.417605	0.342255	0.342255	0.342255
G	0.260343	0.253868	0.247146	0.232978	0.217871	0.184947	0.088058	0.230256	0.235699	0.238420
**TS-4**	**273.15 K, 10 hPa**	**298.15 K, 10 hPa**	**323.15 K, 10 hPa**	**373.15 K, 10 hPa**	**423.15 K, 10 hPa**	**523.15 K, 10 hPa**	**773.15 K, 10 hPa**	**373.15 K, 1 hPa**	**373.15 K, 100 hPa**	**373.15 K, 1000 hPa**
E	-926.7603238									
E+ZPE	-926.454066	-926.454066	-926.454066	-926.454066	-926.454066	-926.454066	-926.454066	-926.454066	-926.454066	-926.454066
E+E_Thermal	-926.436358	-926.433528	-926.430512	-926.423926	-926.416632	-926.400101	-926.349700	-926.423926	-926.423926	-926.423926
H	0.324831	0.327740	0.330836	0.337579	0.345032	0.361879	0.413073	0.337579	0.337579	0.337579
G	0.256171	0.249760	0.243096	0.229033	0.214017	0.181254	0.084737	0.226311	0.231754	0.234475
**I-4**	**273.15 K, 10 hPa**	**298.15 K, 10 hPa**	**323.15 K, 10 hPa**	**373.15 K, 10 hPa**	**423.15 K, 10 hPa**	**523.15 K, 10 hPa**	**773.15 K, 10 hPa**	**373.15 K, 1 hPa**	**373.15 K, 100 hPa**	**373.15 K, 1000 hPa**
E	-817.3553746									
E+ZPE	-817.052879	-817.052879	-817.052879	-817.052879	-817.052879	-817.052879	-817.052879	-817.052879	-817.052879	-817.052879
E+E_Thermal	-817.037341	-817.034812	-817.032102	-817.026149	-817.019505	-817.004314	-816.957422	-817.026149	-817.026149	-817.026149
H	0.318898	0.321507	0.324295	0.330407	0.337209	0.352717	0.400401	0.330407	0.330407	0.330407
G	0.254435	0.248420	0.242180	0.229033	0.215020	0.184489	0.094618	0.226311	0.231754	0.234475
**TS-5**	**273.15 K, 10 hPa**	**298.15 K, 10 hPa**	**323.15 K, 10 hPa**	**373.15 K, 10 hPa**	**423.15 K, 10 hPa**	**523.15 K, 10 hPa**	**773.15 K, 10 hPa**	**373.15 K, 1 hPa**	**373.15 K, 100 hPa**	**373.15 K, 1000 hPa**
E	-817.2855687									
E+ZPE	-816.986829	-816.986829	-816.986829	-816.986829	-816.986829	-816.986829	-816.986829	-816.986829	-816.986829	-816.986829
E+E_Thermal	-816.970802	-816.968196	-816.965418	-816.959350	-816.952622	-816.937349	-816.890625	-816.959350	-816.959350	-816.959350
H	0.315632	0.318317	0.321174	0.327400	0.334286	0.349877	0.397392	0.327400	0.327400	0.327400
G	0.252952	0.247097	0.241010	0.228156	0.214423	0.184430	0.095907	0.225434	0.230877	0.233598
**I-5**	**273.15 K, 10 hPa**	**298.15 K, 10 hPa**	**323.15 K, 10 hPa**	**373.15 K, 10 hPa**	**423.15 K, 10 hPa**	**523.15 K, 10 hPa**	**773.15 K, 10 hPa**	**373.15 K, 1 hPa**	**373.15 K, 100 hPa**	**373.15 K, 1000 hPa**
E	-699.3153163									
E+ZPE	-699.097711	-699.097711	-699.097711	-699.097711	-699.097711	-699.097711	-699.097711	-699.097711	-699.097711	-699.097711
E+E_Thermal	-699.085136	-699.083143	-699.081023	-699.076411	-699.071316	-699.059789	-699.024672	-699.076411	-699.076411	-699.076411
H	0.231045	0.233118	0.235317	0.240087	0.245341	0.257184	0.293093	0.240087	0.240087	0.240087
G	0.175065	0.169851	0.164457	0.153148	0.141166	0.115280	0.040311	0.150426	0.155869	0.158590

TS-6	273.15 K, 10 hPa	298.15 K, 10 hPa	323.15 K, 10 hPa	373.15 K, 10 hPa	423.15 K, 10 hPa	523.15 K, 10 hPa	773.15 K, 10 hPa	373.15 K, 1 hPa	373.15 K, 100 hPa	373.15 K, 1000 hPa
E					-699.2527398					
E+ZPE	-699.037281	-699.037281	-699.037281	-699.037281	-699.037281	-699.037281	-699.037281	-699.037281	-699.037281	-699.037281
E+E_Thermal	-699.024828	-699.022869	-699.020787	-699.016255	-699.011246	-698.999913	-698.965367	-699.016255	-699.016255	-699.016255
H	0.228777	0.230815	0.232976	0.237667	0.242833	0.254484	0.289821	0.237667	0.237667	0.237667
G	0.174091	0.168997	0.163726	0.152672	0.140956	0.115634	0.042238	0.149950	0.155393	0.158114
**I-6**	**273.15 K, 10 hPa**	**298.15 K, 10 hPa**	**323.15 K, 10 hPa**	**373.15 K, 10 hPa**	**423.15 K, 10 hPa**	**523.15 K, 10 hPa**	**773.15 K, 10 hPa**	**373.15 K, 1 hPa**	**373.15 K, 100 hPa**	**373.15 K, 1000 hPa**
E					-699.2842497					
E+ZPE	-699.067007	-699.067007	-699.067007	-699.067007	-699.067007	-699.067007	-699.067007	-699.067007	-699.067007	-699.067007
E+E_Thermal	-699.054324	-699.052323	-699.050197	-699.045573	-699.040468	-699.028924	-698.993780	-699.045573	-699.045573	-699.045573
H	0.230791	0.232871	0.235076	0.239858	0.245122	0.256982	0.292918	0.239858	0.239858	0.239858
G	0.175765	0.170637	0.165330	0.154192	0.142381	0.116831	0.042693	0.151470	0.156913	0.159634
**TS-7**	**273.15 K, 10 hPa**	**298.15 K, 10 hPa**	**323.15 K, 10 hPa**	**373.15 K, 10 hPa**	**423.15 K, 10 hPa**	**523.15 K, 10 hPa**	**773.15 K, 10 hPa**	**373.15 K, 1 hPa**	**373.15 K, 100 hPa**	**373.15 K, 1000 hPa**
E					-699.231617					
E+ZPE	-699.019047	-699.019047	-699.019047	-699.019047	-699.019047	-699.019047	-699.019047	-699.019047	-699.019047	-699.019047
E+E_Thermal	-699.007344	-699.005414	-699.003351	-698.998833	-698.993812	-698.982397	-698.947491	-698.998833	-698.998833	-698.998833
H	0.225138	0.227147	0.229289	0.233966	0.239145	0.250877	0.286574	0.233966	0.233966	0.233966
G	0.172704	0.167817	0.162756	0.152123	0.140830	0.116341	0.044937	0.149401	0.154844	0.157565
**I-7**	**273.15 K, 10 hPa**	**298.15 K, 10 hPa**	**323.15 K, 10 hPa**	**373.15 K, 10 hPa**	**423.15 K, 10 hPa**	**523.15 K, 10 hPa**	**773.15 K, 10 hPa**	**373.15 K, 1 hPa**	**373.15 K, 100 hPa**	**373.15 K, 1000 hPa**
E					-699.3457894					
E+ZPE	-699.128321	-699.128321	-699.128321	-699.128321	-699.128321	-699.128321	-699.128321	-699.128321	-699.128321	-699.128321
E+E_Thermal	-699.116262	-699.114276	-699.112156	-699.107520	-699.102381	-699.090739	-699.055345	-699.107520	-699.107520	-699.107520
H	0.230392	0.232457	0.234657	0.239451	0.244748	0.256707	0.292893	0.239451	0.239451	0.239451
G	0.177292	0.172341	0.167211	0.156429	0.144970	0.120111	0.047617	0.153707	0.159150	0.161871
**TS-8**	**273.15 K, 10 hPa**	**298.15 K, 10 hPa**	**323.15 K, 10 hPa**	**373.15 K, 10 hPa**	**423.15 K, 10 hPa**	**523.15 K, 10 hPa**	**773.15 K, 10 hPa**	**373.15 K, 1 hPa**	**373.15 K, 100 hPa**	**373.15 K, 1000 hPa**

E	-699.2571786									
E+ZPE	-699.046853	-699.046853	-699.046853	-699.046853	-699.046853	-699.046853	-699.046853	-699.046853	-699.046853	-699.046853
E+E_Thermal	-699.034731	-699.032725	-699.030583	-699.025902	-699.020719	-699.009000	-698.973531	-699.025902	-699.025902	-699.025902
H	0.223313	0.225398	0.227619	0.232458	0.237800	0.249835	0.286096	0.232458	0.232458	0.232458
G	0.170278	0.165333	0.160207	0.149426	0.137963	0.113081	0.040489	0.146704	0.152147	0.154868
**I-8**	**273.15 K, 10 hPa**	**298.15 K, 10 hPa**	**323.15 K, 10 hPa**	**373.15 K, 10 hPa**	**423.15 K, 10 hPa**	**523.15 K, 10 hPa**	**773.15 K, 10 hPa**	**373.15 K, 1 hPa**	**373.15 K, 100 hPa**	**373.15 K, 1000 hPa**
E	-581.3037865									
E+ZPE	-581.171194	-581.171194	-581.171194	-581.171194	-581.171194	-581.171194	-581.171194	-581.171194	-581.171194	-581.171194
E+E_Thermal	-581.162584	-581.161192	-581.159711	-581.156494	-581.152959	-581.145043	-581.121444	-581.156494	-581.156494	-581.156494
H	0.142067	0.143539	0.145099	0.148474	0.152168	0.160400	0.184790	0.148474	0.148474	0.148474
G	0.097602	0.093468	0.089206	0.080314	0.070947	0.050869	0.006360	0.077592	0.083035	0.085756
**TS-9**	**273.15 K, 10 hPa**	**298.15 K, 10 hPa**	**323.15 K, 10 hPa**	**373.15 K, 10 hPa**	**423.15 K, 10 hPa**	**523.15 K, 10 hPa**	**773.15 K, 10 hPa**	**373.15 K, 1 hPa**	**373.15 K, 100 hPa**	**373.15 K, 1000 hPa**
E	-581.2571129									
E+ZPE	-581.130280	-581.130280	-581.130280	-581.130280	-581.130280	-581.130280	-581.130280	-581.130280	-581.130280	-581.130280
E+E_Thermal	-581.120922	-581.119460	-581.117912	-581.114572	-581.110934	-581.102882	-581.079295	-581.114572	-581.114572	-581.114572
H	0.137055	0.138597	0.140225	0.143723	0.147519	0.155887	0.180266	0.143723	0.143723	0.143723
G	0.090011	0.085638	0.081131	0.071734	0.061848	0.040702	0.019245	0.069012	0.074455	0.077176
**I-9**	**273.15 K, 10 hPa**	**298.15 K, 10 hPa**	**323.15 K, 10 hPa**	**373.15 K, 10 hPa**	**423.15 K, 10 hPa**	**523.15 K, 10 hPa**	**773.15 K, 10 hPa**	**373.15 K, 1 hPa**	**373.15 K, 100 hPa**	**373.15 K, 1000 hPa**
E	-471.8564333									
E+ZPE	-471.727942	-471.727942	-471.727942	-471.727942	-471.727942	-471.727942	-471.727942	-471.727942	-471.727942	-471.727942
E+E_Thermal	-471.720920	-471.719756	-471.718513	-471.715800	-471.712807	-471.706081	-471.685971	-471.715800	-471.715800	-471.715800
H	0.130810	0.132053	0.133376	0.136247	0.139398	0.146441	0.167343	0.136247	0.136247	0.136247
G	0.090642	0.086911	0.083072	0.075081	0.066686	0.048747	0.002126	0.072359	0.077802	0.080523
**TS-10**	**273.15 K, 10 hPa**	**298.15 K, 10 hPa**	**323.15 K, 10 hPa**	**373.15 K, 10 hPa**	**423.15 K, 10 hPa**	**523.15 K, 10 hPa**	**773.15 K, 10 hPa**	**373.15 K, 1 hPa**	**373.15 K, 100 hPa**	**373.15 K, 1000 hPa**
E	-471.7862563									
E+ZPE	-471.666760	-471.666760	-471.666760	-471.666760	-471.666760	-471.666760	-471.666760	-471.666760	-471.666760	-471.666760

E+E_Thermal	-471.659113	-471.657895	-471.656604	-471.653818	-471.650779	-471.644037	-471.624206	-471.653818	-471.653818	-471.653818
H	0.128009	0.129306	0.130675	0.133620	0.136817	0.143876	0.164499	0.133620	0.133620	0.133620
G	0.086200	0.082317	0.078322	0.070008	0.061281	0.042671	0.009836	0.067285	0.072728	0.075449
**I-10A**	**273.15 K, 10 hPa**	**298.15 K, 10 hPa**	**323.15 K, 10 hPa**	**373.15 K, 10 hPa**	**423.15 K, 10 hPa**	**523.15 K, 10 hPa**	**773.15 K, 10 hPa**	**373.15 K, 1 hPa**	**373.15 K, 100 hPa**	**373.15 K, 1000 hPa**
E	-471.7876269									
E+ZPE	-471.667913	-471.667913	-471.667913	-471.667913	-471.667913	-471.667913	-471.667913	-471.667913	-471.667913	-471.667913
E+E_Thermal	-471.659353	-471.658048	-471.656673	-471.653724	-471.650528	-471.643482	-471.622905	-471.653724	-471.653724	-471.653724
H	0.129139	0.130523	0.131977	0.135084	0.138439	0.145801	0.167170	0.135084	0.135084	0.135084
G	0.085131	0.081042	0.076834	0.068076	0.058885	0.039292	0.015906	0.065354	0.070797	0.073518
**I-10**	**273.15 K, 10 hPa**	**298.15 K, 10 hPa**	**323.15 K, 10 hPa**	**373.15 K, 10 hPa**	**423.15 K, 10 hPa**	**523.15 K, 10 hPa**	**773.15 K, 10 hPa**	**373.15 K, 1 hPa**	**373.15 K, 100 hPa**	**373.15 K, 1000 hPa**
E	-353.8062401									
E+ZPE	-353.768982	-353.768982	-353.768982	-353.768982	-353.768982	-353.768982	-353.768982	-353.768982	-353.768982	-353.768982
E+E_Thermal	-353.764271	-353.763633	-353.762974	-353.761595	-353.760148	-353.757090	-353.748766	-353.761595	-353.761595	-353.761595
H	0.042834	0.043551	0.044290	0.045827	0.047432	0.050807	0.059923	0.045827	0.045827	0.045827
G	0.009581	0.006506	0.003370	0.003074	0.009729	0.023604	0.061027	0.005796	0.000353	0.002368

Table S2: Thermochemical and electronic data from energy calculation and vibrational analysis for **6** and a set of selected associated transition states and intermediates (displayed in first row, first column). Each column, 2–11, have thermochemical data (in Hartree) calculated at a specific temperature and pressure (displayed in the first element of each column). For the first column, E is the electronic energy, E+ZPE is the sum of electronic and zero-point energy, E+E_Thermal is the sum of electronic and thermal energy, H is the enthalpy and G is the Gibbs free energy.

**6**	**273.15 K, 10 hPa**	**298.15 K, 10 hPa**	**323.15 K, 10 hPa**	**373.15 K, 10 hPa**	**423.15 K, 10 hPa**	**523.15 K, 10 hPa**	**773.15 K, 10 hPa**	**373.15 K, 1 hPa**	**373.15 K, 100 hPa**	**373.15 K, 1000 hPa**
E	-1683.013638									
E+ZPE	-1682.230217	-1682.230217	-1682.230217	-1682.230217	-1682.230217	-1682.230217	-1682.230217	-1682.230217	-1682.230217	-1682.230217
E+E_Thermal	-1682.193583	-1682.187125	-1682.180214	-1682.165051	-1682.148158	-1682.109580	-1681.990483	-1682.165051	-1682.165051	-1682.165051
H	0.820920	0.827458	0.834448	0.849768	0.866820	0.905714	1.025603	0.849768	0.849768	0.849768
G	0.719253	0.709661	0.699501	0.677516	0.653359	0.598762	0.428263	0.674794	0.680237	0.682958
**I-1**	**273.15 K, 10 hPa**	**298.15 K, 10 hPa**	**323.15 K, 10 hPa**	**373.15 K, 10 hPa**	**423.15 K, 10 hPa**	**523.15 K, 10 hPa**	**773.15 K, 10 hPa**	**373.15 K, 1 hPa**	**373.15 K, 100 hPa**	**373.15 K, 1000 hPa**

E	-1682.994672									
E+ZPE	-1682.212516	-1682.212516	-1682.212516	-1682.212516	-1682.212516	-1682.212516	-1682.212516	-1682.212516	-1682.212516	-1682.212516
E+E_Thermal	-1682.175513	-1682.169047	-1682.162128	-1682.146949	-1682.130039	-1682.091429	-1681.972256	-1682.146949	-1682.146949	-1682.146949
H	0.820024	0.826569	0.833567	0.848904	0.865973	0.904899	1.024864	0.848904	0.848904	0.848904
G	0.716389	0.706616	0.696276	0.673926	0.649404	0.594068	0.421700	0.671204	0.676647	0.679368
**TS-2**	**273.15 K, 10 hPa**	**298.15 K, 10 hPa**	**323.15 K, 10 hPa**	**373.15 K, 10 hPa**	**423.15 K, 10 hPa**	**523.15 K, 10 hPa**	**773.15 K, 10 hPa**	**373.15 K, 1 hPa**	**373.15 K, 100 hPa**	**373.15 K, 1000 hPa**
E	-1682.922595									
E+ZPE	-1682.145777	-1682.145777	-1682.145777	-1682.145777	-1682.145777	-1682.145777	-1682.145777	-1682.145777	-1682.145777	-1682.145777
E+E_Thermal	-1682.109462	-1682.103064	-1682.096207	-1682.081140	-1682.064325	-1682.025872	-1681.906986	-1682.081140	-1682.081140	-1682.081140
H	0.813997	0.820475	0.827411	0.842637	0.859610	0.898380	1.018057	0.842637	0.842637	0.842637
G	0.710709	0.700972	0.690672	0.668416	0.644001	0.588910	0.417256	0.665694	0.671137	0.673858
**I-2**	**273.15 K, 10 hPa**	**298.15 K, 10 hPa**	**323.15 K, 10 hPa**	**373.15 K, 10 hPa**	**423.15 K, 10 hPa**	**523.15 K, 10 hPa**	**773.15 K, 10 hPa**	**373.15 K, 1 hPa**	**373.15 K, 100 hPa**	**373.15 K, 1000 hPa**
E	-1202.179942									
E+ZPE	-1201.672692	-1201.672692	-1201.672692	-1201.672692	-1201.672692	-1201.672692	-1201.672692	-1201.672692	-1201.672692	-1201.672692
E+E_Thermal	-1201.647732	-1201.643467	-1201.638901	-1201.628875	-1201.617700	-1201.592189	-1201.513555	-1201.628875	-1201.628875	-1201.628875
H	0.533063	0.537406	0.542052	0.552236	0.563569	0.589398	0.668823	0.552236	0.552236	0.552236
G	0.448552	0.440627	0.432324	0.414611	0.395455	0.352968	0.224199	0.411889	0.417332	0.420053
**TS-3**	**273.15 K, 10 hPa**	**298.15 K, 10 hPa**	**323.15 K, 10 hPa**	**373.15 K, 10 hPa**	**423.15 K, 10 hPa**	**523.15 K, 10 hPa**	**773.15 K, 10 hPa**	**373.15 K, 1 hPa**	**373.15 K, 100 hPa**	**373.15 K, 1000 hPa**
E	-1202.09904									
E+ZPE	-1201.598815	-1201.598815	-1201.598815	-1201.598815	-1201.598815	-1201.598815	-1201.598815	-1201.598815	-1201.598815	-1201.598815
E+E_Thermal	-1201.573664	-1201.569388	-1201.564811	-1201.554769	-1201.543582	-1201.518064	-1201.439496	-1201.554769	-1201.554769	-1201.554769
H	0.526241	0.530596	0.535252	0.545453	0.556798	0.582633	0.661993	0.545453	0.545453	0.545453
G	0.441313	0.433349	0.425007	0.407213	0.387974	0.345317	0.216133	0.404491	0.409934	0.412655
**I-4**	**273.15 K, 10 hPa**	**298.15 K, 10 hPa**	**323.15 K, 10 hPa**	**373.15 K, 10 hPa**	**423.15 K, 10 hPa**	**523.15 K, 10 hPa**	**773.15 K, 10 hPa**	**373.15 K, 1 hPa**	**373.15 K, 100 hPa**	**373.15 K, 1000 hPa**
E	-935.3569748									
E+ZPE	-934.971233	-934.971233	-934.971233	-934.971233	-934.971233	-934.971233	-934.971233	-934.971233	-934.971233	-934.971233

E+E_Thermal	-934.952642	-934.949446	-934.946018	-934.938483	-934.930076	-934.910874	-934.851707	-934.938483	-934.938483	-934.938483
H	0.405197	0.408473	0.411980	0.419673	0.428238	0.447757	0.507716	0.419673	0.419673	0.419673
G	0.336534	0.330106	0.323393	0.309131	0.293779	0.259918	0.158235	0.306409	0.311852	0.314573
**TS-5**	**273.15 K, 10 hPa**	**298.15 K, 10 hPa**	**323.15 K, 10 hPa**	**373.15 K, 10 hPa**	**423.15 K, 10 hPa**	**523.15 K, 10 hPa**	**773.15 K, 10 hPa**	**373.15 K, 1 hPa**	**373.15 K, 100 hPa**	**373.15 K, 1000 hPa**
E	-935.2710945									
E+ZPE	-934.908474	-934.908474	-934.908474	-934.908474	-934.908474	-934.908474	-934.908474	-934.908474	-934.908474	-934.908474
E+E_Thermal	-934.889132	-934.885869	-934.882385	-934.874764	-934.866305	-934.847084	-934.788199	-934.874764	-934.874764	-934.874764
H	0.402164	0.405506	0.409069	0.416848	0.425466	0.445004	0.504680	0.416848	0.416848	0.416848
G	0.332439	0.325911	0.319093	0.304610	0.289026	0.254694	0.151873	0.301887	0.307330	0.310051
**I-7**	**273.15 K, 10 hPa**	**298.15 K, 10 hPa**	**323.15 K, 10 hPa**	**373.15 K, 10 hPa**	**423.15 K, 10 hPa**	**523.15 K, 10 hPa**	**773.15 K, 10 hPa**	**373.15 K, 1 hPa**	**373.15 K, 100 hPa**	**373.15 K, 1000 hPa**
E	-778.0098705									
E+ZPE	-777.737030	-777.737030	-777.737030	-777.737030	-777.737030	-777.737030	-777.737030	-777.737030	-777.737030	-777.737030
E+E_Thermal	-777.722826	-777.720389	-777.717784	-777.712082	-777.705759	-777.691432	-777.647840	-777.712082	-777.712082	-777.712082
H	0.287909	0.290426	0.293110	0.298970	0.305451	0.320096	0.364479	0.298970	0.298970	0.298970
G	0.230537	0.225175	0.219596	0.207798	0.195172	0.167543	0.085793	0.205076	0.210519	0.213240
**TS-8**	**273.15 K, 10 hPa**	**298.15 K, 10 hPa**	**323.15 K, 10 hPa**	**373.15 K, 10 hPa**	**423.15 K, 10 hPa**	**523.15 K, 10 hPa**	**773.15 K, 10 hPa**	**373.15 K, 1 hPa**	**373.15 K, 100 hPa**	**373.15 K, 1000 hPa**
E	-777.9274289									
E+ZPE	-777.661682	-777.661682	-777.661682	-777.661682	-777.661682	-777.661682	-777.661682	-777.661682	-777.661682	-777.661682
E+E_Thermal	-777.647268	-777.644818	-777.642200	-777.636480	-777.630143	-777.615804	-777.572268	-777.636480	-777.636480	-777.636480
H	0.281026	0.283555	0.286252	0.292131	0.298626	0.313282	0.357609	0.292131	0.292131	0.292131
G	0.223365	0.217976	0.212369	0.200512	0.187825	0.160072	0.078015	0.197790	0.203233	0.205954

Table S3: Free energies (in kJ mol^{-1}) for the decomposition pathway for **1** (relative to **1** and in kJ mol^{-1}).

	**273.15 K, 10 hPa**	**298.15 K, 10 hPa**	**323.15 K, 10 hPa**	**373.15 K, 10 hPa**	**423.15 K, 10 hPa**	**523.15 K, 10 hPa**	**773.15 K, 10 hPa**	**373.15 K, 1 hPa**	**373.15 K, 100 hPa**	**373.15 K, 1000 hPa**
**TS-1**	135	135	134	133	133	131	128	133	133	133
**I-1**	54	53	52	50	48	44	34	50	50	50

**TS-2**	237	237	237	237	237	237	238	237	237	237
**I-2A**	212	212	211	210	209	207	201	210	210	210
**I-2**	223	216	208	194	180	152	83	187	201	209
**TS-3**	433	426	419	405	391	363	294	398	412	420
**I-3**	362	349	336	310	284	234	108	296	324	339
**TS-4**	472	459	447	421	396	345	220	407	436	450
**I-4**	-14	-31	-49	-83	-118	-187	-357	-105	-62	-40
**TS-5**	166	149	132	98	64	-4	-171	76.2	119	141
**I-5**	126	103	81	35	-11	-101	-325	6	64	92
**TS-6**	288	265	243	198	153	64	-155	169	227	255
**I-6**	210	187	164	119	74.2	-15	-237	91	148	176
**TS-7**	340	318	296	252	208	121	-92.9	223	281	309
**I-7**	52.0	30	8	-36	-81	-168	-386	-65	-8	21
**TS-8**	266	244	222	178	134	46	-172	149	206	235
**I-8**	196	169	141	86.3	32	-77.5	-347	51	122	158
**TS-9**	299	271	243	186	130	18.3	-258	151	222	258
**I-9**	-178	-210	-242	-306	-370	-498	-814	-349	-263	-220
**TS-10**	-20	-52	-85	-150	-215	-344	-665	-193	-107	-64
**I-10A**	-26	-59	-92	-158	-225	-357	-684	-201	-116	-73
**I-10**	-30	-68	-106	-182	-257	-408	-781	-232	-132	-82

Table S4: Free energies for the decomposition pathway for **6** (relative to **6** and in kJ mol^{-1}).

	**273.15 K, 10 hPa**	**298.15 K, 10 hPa**	**323.15 K, 10 hPa**	**373.15 K, 10 hPa**	**423.15 K, 10 hPa**	**523.15 K, 10 hPa**	**773.15 K, 10 hPa**	**373.15 K, 1 hPa**	**373.15 K, 100 hPa**	**373.15 K, 1000 hPa**
**I-1**	42	42	41	40	39	38	33	41	41	41
**TS-2**	217	216	216	215	214	213	210	215	215	215
**I-2**	186	177	169	152	136	103	21	145	159	167
**TS-3**	379	371	362	345	328	295	212	338	352	359
**I-4**	-63	-81	-100	-136	-173	-246	-426	-158	-115	-94

**TS-5**	101	82	64	26	-11	-85	-268	5	48	69
**I-7**	-1	-25	-48	-96	-143	-238	-471	-125	-67	-39
**TS-8**	197	173	149	101	54	-41	-275	73	130	159

Table S5: Enthalpy for the decomposition pathway for **1** (relative to **1** and in kJ mol^{-1}).

	**273.15 K, 10 hPa**	**298.15 K, 10 hPa**	**323.15 K, 10 hPa**	**373.15 K, 10 hPa**	**423.15 K, 10 hPa**	**523.15 K, 10 hPa**	**773.15 K, 10 hPa**	**373.15 K, 1 hPa**	**373.15 K, 100 hPa**	**373.15 K, 1000 hPa**
**TS-1**	139	139	139	139	139	138	137	139	139	139
**I-1**	64	64	64	64	64	64	64	64	64	64
**TS-2**	236	236	236	236	236	235	235	236	236	236
**I-2A**	218	218	218	218	218	219	219	218	218	218
**I-2**	301	300	300	300	299	298	295	300	300	300
**TS-3**	511	511	511	510	510	509	506	510	510	510
**I-3**	503	503	502	502	501	499	494	502	502	502
**TS-4**	612	611	611	611	610	609	603	611	611	611
**I-4**	177	177	176	176	175	173	166	176	176	176
**TS-5**	352	351	351	351	350	349	341	351	351	351
**I-5**	376	376	375	375	374	371	361	375	375	375
**TS-6**	534	534	533	533	531	528	517	533	533	533
**I-6**	457	456	456	456	455	452	442	456	456	456
**TS-7**	580	580	579	578	577	574	564	578	578	578
**I-7**	294	294	294	293	292	290	281	293	293	293
**TS-8**	508	508	508	507	507	505	496	507	507	507
**I-8**	497	496	496	495	494	491	479	495	495	495
**TS-9**	606	606	606	606	605	602	590	606	606	606
**I-9**	174	174	174	173	172	169	155	173	173	173
**I-10A**	335	336	336	336	335	333	321	336	336	336
**I-10**	384	384	384	384	383	379	363	384	384	384

Table S6: Enthalpy for the decomposition pathway for **6** (Relative to **6** and in kJ mol^{-1}).

	273.15 K, 10 hPa	298.15 K, 10 hPa	323.15 K, 10 hPa	373.15 K, 10 hPa	423.15 K, 10 hPa	523.15 K, 10 hPa	773.15 K, 10 hPa	373.15 K, 1 hPa	373.15 K, 100 hPa	373.15 K, 1000 hPa
**I-1**	47	47	47	48	48	48	48	48	48	48
**TS-2**	221	221	221	220	220	220	219	220	220	220
**I-2**	277	277	277	276	276	275	272	276	276	276
**TS-3**	472	471	471	471	470	469	466	471	471	471
**I-4**	138	138	137	137	136	134	126	137	137	137
**TS-5**	305	305	304	304	303	301	293	304	304	304
**I-7**	260	259	259	258	257	255	245	258	258	258
**TS-8**	458	458	458	457	456	453	443	457	457	457

NBO charges for **1**, **6** and form-amidinate analog of **1**

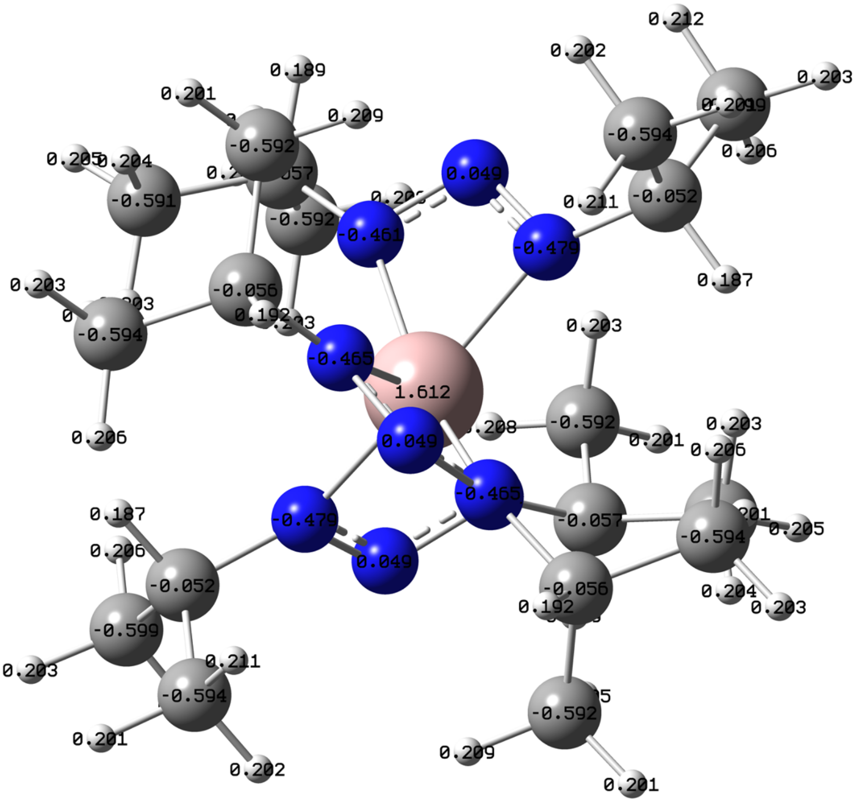

Figure S61: NBO charges of **1**.

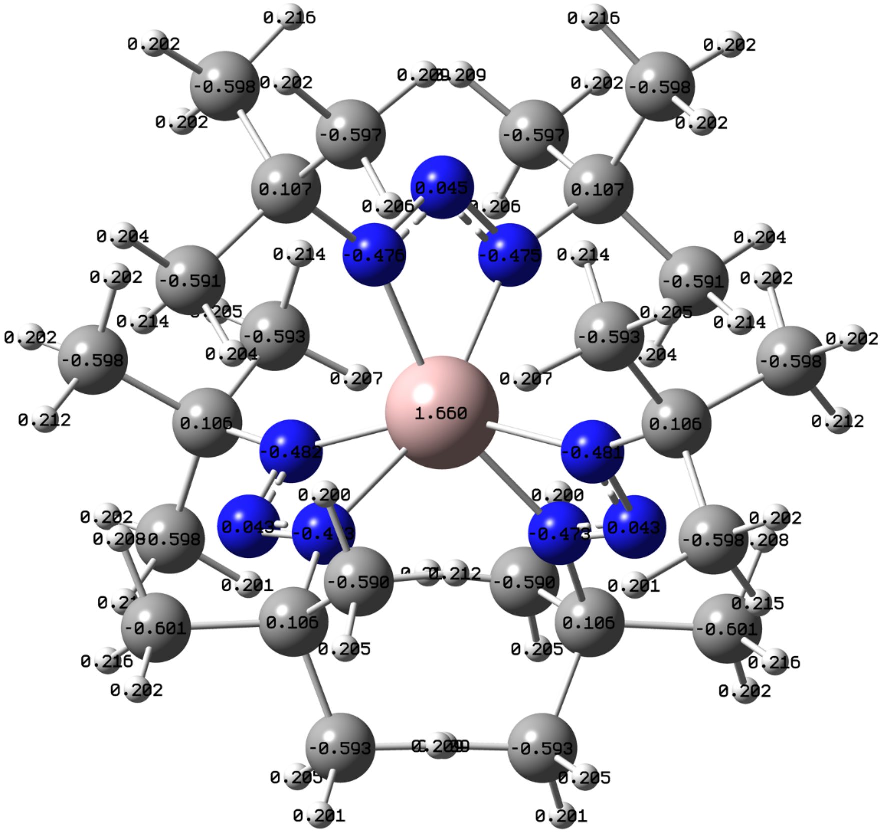

Figure S62: NBO charges of **6**.

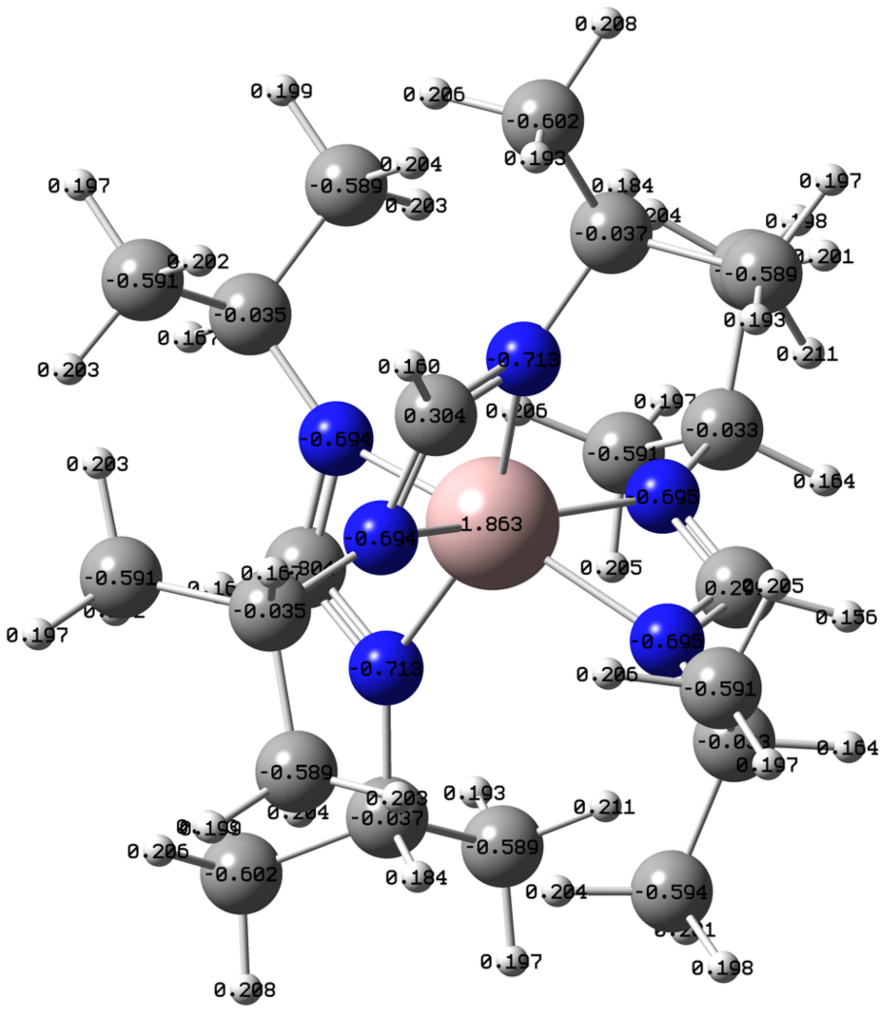

Figure S63: NBO charges of Al(*i*Pr$_2$-f-amd)$_3$.

Cartesian coordinates for **1** and associated intermediate and transition state structures

Table S7: Cartesian coordinates for geometry of **1**.

Center Number	Atomic Number	Coordinates (Å) X	Y	Z
1	7	1.197936	-1.760846	1.138123
2	7	1.71324	-0.902799	0.315801
3	6	3.119208	-1.039901	-0.042761
4	6	3.993954	-1.482795	1.128407
5	1	3.735824	-2.491455	1.450573
6	1	5.044274	-1.472523	0.831866
7	1	3.868111	-0.81354	1.981221
8	6	3.274979	-1.959812	-1.257973
9	1	2.647659	-1.616042	-2.081204
10	1	4.312962	-1.982562	-1.597344
11	1	2.972685	-2.976695	-0.998483
12	7	-0.072371	-1.526494	1.207942

13	6	-0.834621	-2.298415	2.180588
14	6	-0.974372	-1.529036	3.496407
15	1	-1.494967	-0.584401	3.327787
16	1	-1.543002	-2.111863	4.224546
17	1	0.005215	-1.308271	3.918998
18	7	-0.457989	-0.943487	-1.807213
19	7	0.457764	0.944168	-1.806937
20	7	-0.000135	0.000463	-2.563155
21	6	-1.033152	-2.095048	-2.487454
22	6	1.032739	2.096016	-2.486852
23	6	-2.551546	-2.12124	-2.297216
24	6	0.369357	3.378629	-1.984956
25	1	-3.001905	-1.209002	-2.691458
26	1	-2.991873	-2.975816	-2.815486
27	1	-2.805375	-2.197143	-1.239338
28	1	0.48779	3.474008	-0.905047
29	1	0.814416	4.254775	-2.460671
30	1	-0.698589	3.370161	-2.203112
31	7	0.072593	1.526038	1.20852
32	7	-1.713141	0.902694	0.316376
33	7	-1.197713	1.760454	1.138922
34	6	0.834958	2.297531	2.181419
35	6	-3.119158	1.03993	-0.04194
36	6	2.205239	2.636825	1.599935
37	6	-3.993742	1.482403	1.129508
38	6	-3.275087	1.960286	-1.256796
39	1	2.114852	3.257093	0.708994
40	1	2.808431	3.176327	2.331952
41	1	2.736584	1.722008	1.329088
42	1	-3.735562	2.490945	1.452005
43	1	-5.044102	1.472245	0.833107
44	1	-3.867786	0.812838	1.982062
45	1	-2.64788	1.616812	-2.080237
46	1	-4.313116	1.983164	-1.596018
47	1	-2.972752	2.977072	-0.996977
48	6	0.97471	1.527644	3.496939
49	1	-0.004874	1.306823	3.919508
50	1	1.495195	0.583016	3.327933
51	1	1.543445	2.110137	4.225262
52	6	2.551148	2.122324	-2.296744
53	1	2.805065	2.197937	-1.238868
54	1	3.001578	1.21026	-2.691305
55	1	2.991328	2.977109	-2.814795

56	6	-0.369883	-3.377897	-1.986011
57	1	0.698049	-3.369483	-2.204237
58	1	-0.488257	-3.473605	-0.906122
59	1	-0.815077	-4.253839	-2.461975
60	6	-2.204896	-2.637602	1.599029
61	1	-2.73633	-1.722727	1.328552
62	1	-2.114486	-3.257521	0.707846
63	1	-2.808014	-3.177439	2.330861
64	1	3.42638	-0.034726	-0.344
65	1	0.817117	1.976043	-3.553152
66	1	-0.81761	-1.974759	-3.553734
67	1	-0.281048	-3.223769	2.371521
68	1	-3.426375	0.034865	-0.343503
69	1	0.281469	3.222857	2.372731
70	13	0.000019	0.000023	-0.09686

Table S8: Cartesian coordinates for **1** adopting **TS-1** structure.

Center	Atomic	Coordinates (Å)		
Number	Number	X	Y	Z
1	7	-0.645752	0.679177	-1.908889
2	7	-0.054312	-0.457691	-1.641185
3	6	-0.115136	-1.481353	-2.675064
4	6	1.188307	-1.51141	-3.477496
5	1	2.034691	-1.723457	-2.822144
6	1	1.15179	-2.280314	-4.252516
7	1	1.362702	-0.54643	-3.955739
8	6	-0.435051	-2.835185	-2.046057
9	1	-1.365325	-2.794019	-1.479892
10	1	-0.524082	-3.603296	-2.816259
11	1	0.355581	-3.136946	-1.359364
12	7	-0.431554	1.479356	-0.932615
13	6	-1.123212	2.769703	-0.963245
14	6	-0.586675	3.663365	-2.083266
15	1	-0.782746	3.206179	-3.054565
16	1	-1.072296	4.640803	-2.057519
17	1	0.489549	3.81256	-1.986085
18	7	2.252052	0.926305	0.463406
19	7	2.205057	-1.171282	0.466793
20	7	2.963289	-0.158413	0.650312
21	6	2.982898	2.186953	0.450071
22	6	2.778277	-2.477891	0.786318
23	6	2.135202	3.286829	1.082241
24	6	1.714238	-3.334123	1.469485

25	1	1.854552	3.030436	2.104256
26	1	2.687527	4.22756	1.102933
27	1	1.222534	3.446489	0.50756
28	1	0.844218	-3.464802	0.824615
29	1	2.118665	-4.319746	1.706725
30	1	1.366251	-2.866443	2.389572
31	7	-2.669959	-0.524714	0.687231
32	7	-0.584804	-0.405352	1.477482
33	7	-1.718211	-1.191608	1.088187
34	6	-3.820023	-1.350594	0.27909
35	6	-0.710304	0.077968	2.851769
36	6	-4.11917	-1.042088	-1.186746
37	6	0.660796	0.157048	3.523595
38	6	-1.447244	1.42235	2.95209
39	1	-3.25619	-1.259265	-1.816892
40	1	-4.960529	-1.646052	-1.53146
41	1	-4.371955	0.01136	-1.314972
42	1	1.308837	0.869405	3.009408
43	1	0.560073	0.488839	4.559009
44	1	1.155822	-0.813683	3.51557
45	1	-2.416536	1.366865	2.458534
46	1	-1.599798	1.714098	3.995678
47	1	-0.864546	2.205055	2.458355
48	6	-4.994485	-1.004717	1.191034
49	1	-4.760872	-1.237917	2.230963
50	1	-5.233234	0.058657	1.122971
51	1	-5.87784	-1.57585	0.899476
52	6	3.34138	-3.15131	-0.467032
53	1	2.551353	-3.363309	-1.188141
54	1	4.081634	-2.509712	-0.947266
55	1	3.821727	-4.096453	-0.205153
56	6	3.404674	2.544998	-0.97788
57	1	4.040658	1.762619	-1.394397
58	1	2.525681	2.645357	-1.617694
59	1	3.956781	3.487325	-0.998175
60	6	-2.641816	2.60282	-1.036398
61	1	-2.927952	2.158568	-1.991183
62	1	-2.985376	1.948533	-0.236375
63	1	-3.133273	3.57372	-0.945683
64	1	-0.932159	-1.199095	-3.346453
65	1	3.604035	-2.306893	1.483758
66	1	3.883478	2.038227	1.054257
67	1	-0.880843	3.227126	-0.001399

68	1	-1.30177	-0.669528	3.3985
69	1	-3.557916	-2.407997	0.394782
70	13	0.564654	0.040832	0.124024

Table S9: Cartesian coordinates for **1** adopting **I-1** structure.

Center	Atomic	Coordinates (Å)		
Number	Number	X	Y	Z
1	7	-0.383032	1.259677	1.936949
2	7	-0.633371	-0.011216	1.796341
3	6	-0.948863	-0.766109	2.999336
4	6	-2.444631	-1.088701	3.043986
5	1	-2.734486	-1.673411	2.169478
6	1	-2.691791	-1.66359	3.939025
7	1	-3.033561	-0.170302	3.048704
8	6	-0.084854	-2.024305	3.058526
9	1	0.974148	-1.766682	3.031063
10	1	-0.288166	-2.58701	3.971474
11	1	-0.294625	-2.674018	2.207792
12	7	-0.229493	1.762701	0.763388
13	6	0.255175	3.137194	0.666032
14	6	-0.566542	4.107109	1.514441
15	1	-0.471364	3.866798	2.573665
16	1	-0.218861	5.13004	1.360175
17	1	-1.623544	4.058114	1.246851
18	7	-2.22411	0.255348	-0.997258
19	7	-1.516331	-1.690888	-0.689252
20	7	-2.496266	-1.010001	-1.166567
21	6	-3.27294	1.218543	-1.296819
22	6	-1.537385	-3.134843	-0.919807
23	6	-2.670846	2.420802	-2.020594
24	6	-0.102343	-3.650732	-0.864257
25	1	-2.188102	2.118337	-2.950618
26	1	-3.444758	3.153002	-2.256156
27	1	-1.924995	2.909137	-1.390698
28	1	0.338679	-3.464012	0.116714
29	1	-0.079207	-4.725594	-1.049214
30	1	0.524364	-3.154061	-1.604577
31	7	3.211657	-0.372137	-0.524776
32	7	1.070056	-0.181672	-1.111238
33	7	2.009868	-0.348617	-0.175563
34	6	4.116309	-0.607398	0.605732
35	6	1.449327	-0.000995	-2.516647
36	6	5.118801	0.54318	0.680497
37	6	0.284438	-0.390949	-3.420807

38	6	1.906187	1.437944	-2.778462
39	1	4.607566	1.491908	0.852351
40	1	5.830512	0.38425	1.493755
41	1	5.676561	0.622177	-0.255386
42	1	-0.585604	0.245938	-3.245657
43	1	0.569733	-0.28087	-4.46852
44	1	-0.018019	-1.425407	-3.255801
45	1	2.73356	1.70152	-2.12145
46	1	2.237751	1.557589	-3.812773
47	1	1.081805	2.134442	-2.602423
48	6	4.814776	-1.952511	0.406845
49	1	4.085395	-2.763902	0.375561
50	1	5.36854	-1.957307	-0.534762
51	1	5.516315	-2.149222	1.220891
52	6	-2.44451	-3.847831	0.085161
53	1	-2.062778	-3.736128	1.101181
54	1	-3.453756	-3.435828	0.048493
55	1	-2.49838	-4.914569	-0.142117
56	6	-3.987216	1.635832	-0.007788
57	1	-4.424476	0.76695	0.485962
58	1	-3.27848	2.099235	0.681857
59	1	-4.783636	2.352793	-0.218428
60	6	1.750791	3.203049	0.989024
61	1	1.919126	2.938403	2.034824
62	1	2.307789	2.502722	0.367026
63	1	2.138057	4.209575	0.817893
64	1	-0.700001	-0.121509	3.847457
65	1	-1.938144	-3.30463	-1.924553
66	1	-3.98954	0.718771	-1.955834
67	1	0.125678	3.397036	-0.387905
68	1	2.294203	-0.6676	-2.711499
69	1	3.530854	-0.639408	1.533258
70	13	-0.508558	-0.01244	-0.139049

Table S10: Cartesian coordinates for **1** adopting **TS-2** structure.

Center	Atomic	Coordinates (Å)		
Number	Number	X	Y	Z
1	7	2.511687	-1.38624	0.211664
2	7	0.970053	-0.330804	1.32007
3	6	3.584436	-2.392978	0.211955
4	6	4.769956	-1.952222	1.067782
5	1	4.475132	-1.870481	2.114418
6	1	5.579241	-2.680531	0.993105
7	1	5.147863	-0.982822	0.74034

8	6	3.055076	-3.770104	0.619148
9	1	2.214101	-4.066937	-0.009892
10	1	3.837393	-4.525463	0.525341
11	1	2.715865	-3.749328	1.656175
12	6	0.437546	-0.042846	2.667873
13	6	0.848486	1.359113	3.116822
14	1	0.421222	2.118269	2.462321
15	1	0.500459	1.547016	4.134519
16	1	1.934332	1.463958	3.098162
17	6	-1.074377	-0.248844	2.70604
18	1	-1.340915	-1.257788	2.389444
19	1	-1.44089	-0.096694	3.722677
20	1	-1.600487	0.446956	2.051591
21	7	-1.37296	-0.560749	-0.84752
22	6	-0.942672	-1.819319	-1.48189
23	7	-3.39191	-1.309517	-0.371769
24	6	-1.682774	-2.142944	-2.77943
25	6	0.557051	-1.543575	-1.702731
26	6	-4.670329	-0.984806	0.268293
27	1	-1.606935	-1.299442	-3.470355
28	1	-1.23076	-3.016074	-3.256636
29	1	-2.73492	-2.347562	-2.59217
30	1	1.088567	-2.484578	-1.873776
31	1	0.691982	-0.969068	-2.626759
32	1	1.632985	-1.343942	-0.69011
33	6	-5.789386	-1.14025	-0.760303
34	6	-4.868814	-1.916052	1.464316
35	1	-5.80846	-2.160533	-1.150114
36	1	-6.76132	-0.924821	-0.310589
37	1	-5.639125	-0.459492	-1.599993
38	1	-4.077345	-1.770164	2.20175
39	1	-5.830005	-1.726441	1.947555
40	1	-4.844343	-2.959294	1.141431
41	7	-0.270702	2.13745	-0.049047
42	7	1.447284	1.558139	-1.102058
43	6	2.76396	1.850257	-1.658939
44	1	2.692666	2.824281	-2.153899
45	6	-1.302778	3.088673	0.36243
46	1	-1.760564	2.63796	1.246975
47	6	-2.378478	3.18858	-0.72286
48	1	-2.774187	2.200119	-0.953736
49	1	-3.199675	3.824874	-0.385871
50	1	-1.954305	3.623709	-1.630758

51	6	-0.736817	4.454386	0.743051
52	1	0.040927	4.363088	1.503035
53	1	-0.304191	4.956093	-0.122334
54	1	-1.536439	5.081357	1.140307
55	6	3.809183	1.941684	-0.543683
56	1	4.793563	2.179079	-0.953308
57	1	3.536019	2.718512	0.171764
58	1	3.872912	0.990667	-0.012362
59	6	3.133286	0.791058	-2.692086
60	1	3.212248	-0.191453	-2.22457
61	1	2.384838	0.734125	-3.483183
62	1	4.09691	1.029274	-3.144796
63	1	-1.083499	-2.649749	-0.777287
64	1	3.904941	-2.436559	-0.832597
65	1	-4.642651	0.054059	0.620938
66	1	0.908029	-0.771951	3.331662
67	7	2.009511	-1.101233	1.354458
68	7	-2.575675	-0.363709	-0.310912
69	7	0.762877	2.582745	-0.670281
70	13	0.214024	0.234333	-0.384259

Table S11: Cartesian coordinates for **1** adopting **I-2A** structure.

Center Number	Atomic Number	Coordinates (Å) X	Y	Z
1	7	2.900634	-1.151501	0.198604
2	7	1.126895	-0.464385	1.300494
3	6	4.112072	-1.982474	0.173594
4	6	5.111907	-1.521757	1.228566
5	1	4.716825	-1.679276	2.231834
6	1	6.036719	-2.091091	1.129791
7	1	5.341926	-0.462185	1.111159
8	6	3.764703	-3.466636	0.298295
9	1	3.057459	-3.770168	-0.474826
10	1	4.663869	-4.077217	0.201744
11	1	3.314887	-3.664599	1.273006
12	6	0.569248	-0.316192	2.672977
13	6	0.889033	1.074967	3.214574
14	1	0.427429	1.84508	2.599998
15	1	0.511835	1.172308	4.234165
16	1	1.966829	1.243968	3.22716
17	6	-0.923325	-0.624223	2.681966
18	1	-1.127465	-1.613831	2.272143
19	1	-1.287415	-0.595847	3.710233
20	1	-1.498794	0.094587	2.09971

21	7	-1.535255	-0.615646	-0.6878
22	6	-1.175617	-1.811672	-1.488187
23	7	-3.622858	-1.24907	-0.382995
24	6	-1.802718	-1.796953	-2.880312
25	6	0.3735	-1.687591	-1.49758
26	6	-4.888643	-0.913156	0.273717
27	1	-1.481208	-0.905101	-3.425689
28	1	-1.482202	-2.675381	-3.445924
29	1	-2.890033	-1.794928	-2.819479
30	1	0.823134	-2.563114	-1.019968
31	1	0.764214	-1.645411	-2.518051
32	1	2.333807	-1.08958	-0.651923
33	6	-5.987022	-0.830011	-0.785484
34	6	-5.197548	-1.982149	1.322574
35	1	-6.072634	-1.779615	-1.31871
36	1	-6.952589	-0.603125	-0.327288
37	1	-5.75987	-0.051332	-1.515764
38	1	-4.417529	-2.00819	2.085974
39	1	-6.153862	-1.782524	1.811977
40	1	-5.247511	-2.968337	0.855298
41	7	-0.272418	1.962505	0.119932
42	7	1.335214	1.484909	-1.137658
43	6	2.492729	1.874177	-1.935962
44	1	2.245325	2.823558	-2.422634
45	6	-1.289995	2.877506	0.644051
46	1	-1.733979	2.345229	1.488556
47	6	-2.387074	3.097955	-0.401223
48	1	-2.804547	2.14283	-0.716345
49	1	-3.190613	3.71018	0.014159
50	1	-1.974084	3.61338	-1.271443
51	6	-0.702164	4.197224	1.142124
52	1	0.088605	4.029829	1.875376
53	1	-0.280046	4.771585	0.317682
54	1	-1.485862	4.794085	1.611518
55	6	3.724439	2.094567	-1.052951
56	1	4.579078	2.417071	-1.652192
57	1	3.520606	2.859478	-0.302547
58	1	3.992528	1.171457	-0.535011
59	6	2.747057	0.826572	-3.015611
60	1	3.026064	-0.134813	-2.579001
61	1	1.857957	0.668039	-3.626056
62	1	3.564596	1.144983	-3.664201
63	1	-1.512182	-2.709857	-0.957818

64	1	4.5365	-1.798129	-0.816522
65	1	-4.789794	0.060427	0.771209
66	1	1.088836	-1.057076	3.28473
67	7	2.275412	-0.995692	1.328091
68	7	-2.732059	-0.393092	-0.165464
69	7	0.65796	2.465184	-0.605512
70	13	0.16654	0.049944	-0.422357

Table S12: Cartesian coordinates for **1** adopting **I-2** structure.

Center	Atomic	Coordinates (Å)		
Number	Number	X	Y	Z
1	7	-0.890565	-0.353487	0.463309
2	6	-1.121515	-0.861517	1.85512
3	7	-3.029487	-0.187486	-0.002889
4	6	-1.657628	-2.289166	1.879732
5	6	0.316241	-0.730602	2.457146
6	6	-4.019345	0.219727	-1.004663
7	1	-0.956283	-2.964915	1.381723
8	1	-1.778161	-2.626104	2.912074
9	1	-2.619435	-2.352466	1.373911
10	1	0.359734	0.005062	3.261385
11	1	0.691199	-1.675833	2.850529
12	6	-4.867616	-0.996189	-1.376224
13	6	-4.872678	1.344529	-0.419347
14	1	-5.362625	-1.400075	-0.490096
15	1	-5.634129	-0.723829	-2.105273
16	1	-4.246228	-1.783368	-1.806714
17	1	-4.255711	2.211726	-0.177142
18	1	-5.641402	1.655571	-1.130352
19	1	-5.364378	1.011124	0.497314
20	7	1.915427	1.372058	-0.018
21	7	2.312755	-0.62377	-0.54842
22	6	3.082782	-1.681504	-1.190241
23	1	3.703514	-1.204751	-1.954311
24	6	2.053405	2.816109	-0.161167
25	1	3.054329	3.006634	-0.55968
26	6	1.918186	3.464561	1.214087
27	1	2.67847	3.090532	1.901094
28	1	2.024089	4.547391	1.136007
29	1	0.935218	3.252889	1.642129
30	6	1.008965	3.343618	-1.147683
31	1	1.140673	2.885557	-2.128706
32	1	0.000886	3.110588	-0.797804
33	1	1.096554	4.426503	-1.255589

34	6	3.987211	-2.370434	-0.166391
35	1	4.570635	-3.161258	-0.641628
36	1	4.676863	-1.655348	0.284237
37	1	3.389691	-2.819651	0.630402
38	6	2.118972	-2.663782	-1.852774
39	1	1.461593	-3.118021	-1.10753
40	1	1.494913	-2.160555	-2.591862
41	1	2.671473	-3.463366	-2.348539
42	1	-1.835161	-0.199521	2.355534
43	1	-3.498185	0.585858	-1.897899
44	7	-1.854546	-0.025478	-0.396839
45	7	2.682866	0.60563	-0.729518
46	13	0.89268	-0.120365	0.682131

Table S13: Cartesian coordinates for **1** adopting **TS-3** structure.

Center	Atomic	Coordinates (Å)		
Number	Number	X	Y	Z
1	7	1.131653	-0.629385	-0.484277
2	6	1.221284	-1.604891	-1.607867
3	7	3.388408	-0.301055	-0.146907
4	6	1.782797	-2.952547	-1.172262
5	6	-0.275844	-1.654207	-2.058334
6	6	4.128432	1.093908	1.411959
7	1	1.14939	-3.394039	-0.397792
8	1	1.814046	-3.638814	-2.021484
9	1	2.791561	-2.840997	-0.775381
10	1	-0.410568	-1.301042	-3.081102
11	1	-0.692948	-2.6596	-1.994918
12	6	2.929279	1.600217	1.902002
13	6	4.942788	0.079485	2.155775
14	1	2.612344	2.594943	1.60416
15	1	2.091433	0.747555	1.007123
16	1	2.595269	1.289899	2.888837
17	1	5.528287	-0.540327	1.475549
18	1	5.63684	0.572693	2.846295
19	1	4.301993	-0.57743	2.749155
20	7	-1.506106	1.354798	-0.418424
21	7	-1.904748	-0.260284	0.86927
22	6	-2.524759	-0.89817	2.023972
23	1	-3.212897	-0.167321	2.457702
24	6	-1.550304	2.75762	-0.813247
25	1	-2.388733	3.213207	-0.278738
26	6	-1.791862	2.849291	-2.318076
27	1	-2.731214	2.368611	-2.594482

28	1	-1.830252	3.892606	-2.6344
29	1	-0.98231	2.361383	-2.866546
30	6	-0.249635	3.449593	-0.398602
31	1	-0.101376	3.381829	0.679658
32	1	0.607173	2.979805	-0.886777
33	1	-0.269865	4.50361	-0.681646
34	6	-3.308667	-2.128804	1.569733
35	1	-3.780747	-2.616787	2.423922
36	1	-4.085512	-1.855151	0.854576
37	1	-2.643287	-2.852397	1.092617
38	6	-1.447511	-1.25336	3.049958
39	1	-0.72099	-1.94596	2.618767
40	1	-0.912418	-0.36078	3.375944
41	1	-1.894867	-1.729535	3.924182
42	1	1.862156	-1.184082	-2.390408
43	1	4.658511	1.655399	0.652264
44	7	2.236664	-0.09887	0.086826
45	7	-2.167022	0.988162	0.63572
46	13	-0.659148	-0.377646	-0.614021

Table S14: Cartesian coordinates for **1** adopting **I-3** structure.

Center	Atomic	Coordinates (Å)		
Number	Number	X	Y	Z
1	7	-1.745864	-0.376899	0.35254
2	6	-2.531213	-0.4256	-0.906206
3	7	-3.419816	-1.352151	1.688689
4	6	-3.677052	0.580378	-0.927813
5	6	-1.414088	-0.12717	-1.96268
6	1	-3.289382	1.596574	-0.812355
7	1	-4.209788	0.522627	-1.87999
8	1	-4.381427	0.380237	-0.121149
9	1	-1.279769	-0.950971	-2.664322
10	1	-1.623879	0.773894	-2.539481
11	1	-1.622894	-0.750326	2.295763
12	7	1.555823	-0.822877	-0.043447
13	7	1.196105	1.242452	0.123583
14	6	1.574677	2.62483	0.388704
15	1	2.634771	2.617052	0.657204
16	6	2.375484	-2.024995	0.043092
17	1	3.390829	-1.699887	0.286765
18	6	2.379883	-2.739069	-1.308194
19	1	2.776448	-2.090882	-2.090695
20	1	2.99284	-3.640584	-1.261367
21	1	1.366014	-3.034038	-1.589125

22	6	1.848291	-2.925377	1.161301
23	1	1.87725	-2.408772	2.121635
24	1	0.815146	-3.21909	0.961556
25	1	2.450421	-3.832294	1.238821
26	6	1.376803	3.463331	-0.874286
27	1	1.660238	4.501233	-0.69185
28	1	1.981918	3.078574	-1.696218
29	1	0.329191	3.447808	-1.184167
30	6	0.75579	3.162203	1.562744
31	1	-0.311675	3.12593	1.333435
32	1	0.931521	2.570712	2.46222
33	1	1.023702	4.199043	1.772901
34	1	-2.941066	-1.433368	-1.033949
35	7	-2.337773	-0.875991	1.537299
36	7	2.09922	0.319016	0.245142
37	13	-0.164287	-0.016885	-0.457865

Table S15: Cartesian coordinates for **1** adopting **TS-4** structure.

Center Number	Atomic Number	Coordinates (Å) X	Y	Z
1	7	1.655972	-0.557412	0.900208
2	6	2.473799	-0.790602	-0.311929
3	7	3.23123	1.66435	1.612771
4	6	3.251517	-2.103774	-0.233022
5	6	1.486656	-0.714607	-1.538248
6	1	2.559647	-2.948418	-0.177615
7	1	3.886596	-2.235241	-1.113214
8	1	3.880481	-2.12706	0.659732
9	1	1.778395	0.031346	-2.279164
10	1	1.390464	-1.676417	-2.046324
11	1	2.057112	0.094256	2.44663
12	7	-1.104962	1.180062	0.033172
13	7	-1.65556	-0.841199	0.203796
14	6	-2.591956	-1.923712	0.472402
15	1	-3.599819	-1.509307	0.374505
16	6	-1.382955	2.604543	-0.088875
17	1	-2.366052	2.781857	0.357743
18	6	-1.422939	3.012749	-1.563395
19	1	-2.194358	2.455243	-2.096305
20	1	-1.635806	4.079129	-1.661736
21	1	-0.461794	2.809678	-2.041251
22	6	-0.321284	3.382239	0.686066
23	1	-0.321373	3.099787	1.739757
24	1	0.67351	3.183062	0.280431

25	1	-0.505568	4.455222	0.615458
26	6	-2.391123	-3.028092	-0.563585
27	1	-3.083882	-3.851617	-0.3833
28	1	-2.555303	-2.650664	-1.573628
29	1	-1.373957	-3.423408	-0.508641
30	6	-2.396716	-2.440598	1.899583
31	1	-1.377733	-2.810505	2.033967
32	1	-2.569362	-1.644011	2.624737
33	1	-3.089963	-3.257155	2.111075
34	1	3.206539	0.034965	-0.401345
35	7	2.447508	0.91824	1.943483
36	7	-2.105444	0.375039	0.213274
37	13	0.184663	-0.283362	-0.145597

Table S16: Cartesian coordinates for **1** adopting **I-4** structure.

Center Number	Atomic Number	Coordinates (Å) X	Y	Z
1	7	0.971053	-1.701814	-1.132628
2	6	1.510661	-2.585424	-0.06746
3	6	3.025922	-2.749265	-0.14906
4	6	1.034336	-1.861261	1.238523
5	1	3.51614	-1.777961	-0.037773
6	1	3.391555	-3.415345	0.637234
7	1	3.324477	-3.166379	-1.115101
8	1	0.366302	-2.478026	1.841149
9	1	1.870602	-1.560481	1.871322
10	1	1.150312	-1.962294	-2.087568
11	7	-1.527488	0.285247	-0.018862
12	7	0.249829	1.40635	-0.019054
13	6	1.019641	2.640965	0.039223
14	1	0.300776	3.463543	0.096691
15	6	-2.973869	0.123527	0.027463
16	1	-3.404391	1.124143	0.12813
17	6	-3.356988	-0.71812	1.245246
18	1	-3.019739	-0.243487	2.167615
19	1	-4.439605	-0.847228	1.296654
20	1	-2.89907	-1.708201	1.184737
21	6	-3.462101	-0.509888	-1.276166
22	1	-3.206173	0.117291	-2.131175
23	1	-2.999116	-1.48861	-1.420899
24	1	-4.545409	-0.643223	-1.255824
25	6	1.894255	2.644418	1.293849
26	1	2.477002	3.565544	1.352533
27	1	1.283197	2.562582	2.193702

28	1	2.58997	1.802247	1.276679
29	6	1.854434	2.78325	-1.234051
30	1	2.540008	1.939013	-1.336423
31	1	1.213929	2.809854	-2.116607
32	1	2.444151	3.70142	-1.205541
33	1	1.054005	-3.58393	-0.124491
34	7	-1.043334	1.48661	0.026655
35	13	0.243301	-0.55034	0.006676

Table S17: Cartesian coordinates for **1** adopting **TS-5** structure.

Center	Atomic		Coordinates (Å)	
Number	Number	X	Y	Z
1	7	0.334725	1.93102	1.753169
2	6	1.516179	2.435192	-0.443958
3	6	2.93781	2.028396	-0.255305
4	6	0.628221	1.788245	-1.268456
5	1	3.113582	0.991422	-0.543209
6	1	3.56609	2.661473	-0.894069
7	1	3.247247	2.175403	0.777014
8	1	-0.281814	2.288764	-1.579162
9	1	0.956271	0.947488	-1.870697
10	1	0.420685	1.896935	2.759048
11	7	-1.495255	-0.366706	-0.062342
12	7	0.463278	-1.106742	0.13454
13	6	1.476254	-2.149341	0.023803
14	1	0.966906	-3.111113	0.139365
15	6	-2.925131	-0.498992	-0.30535
16	1	-3.079606	-1.457932	-0.809123
17	6	-3.38439	0.636738	-1.218551
18	1	-2.852102	0.611382	-2.170672
19	1	-4.454269	0.558497	-1.41835
20	1	-3.198515	1.604031	-0.745702
21	6	-3.682296	-0.499103	1.024438
22	1	-3.35772	-1.330217	1.651973
23	1	-3.497596	0.430708	1.566731
24	1	-4.756916	-0.592052	0.854577
25	6	2.142205	-2.113665	-1.354246
26	1	2.877035	-2.915893	-1.447212
27	1	1.398398	-2.233865	-2.143255
28	1	2.65778	-1.163386	-1.510043
29	6	2.489059	-1.974165	1.15196
30	1	2.978356	-1.000343	1.082383
31	1	2.002301	-2.033525	2.1258
32	1	3.257112	-2.747176	1.097069

33	1	1.224961	3.371669	0.006583
34	7	-0.751582	-1.419025	-0.190888
35	13	-0.017985	0.758313	0.577199

Table S18: Cartesian coordinates for **1** adopting **I-5** structure.

Center	Atomic	Coordinates (Å)		
Number	Number	X	Y	Z
1	7	0.479475	2.939519	0.259763
2	1	-0.008707	3.819426	0.301843
3	7	-1.095478	-0.210871	-0.136143
4	7	1.002741	-0.299945	-0.001951
5	6	2.305793	-0.95594	-0.034766
6	1	2.117105	-2.0324	-0.000153
7	6	-2.433136	-0.793623	-0.184116
8	1	-2.334574	-1.778346	-0.6499
9	6	-3.32601	0.091758	-1.048747
10	1	-2.929128	0.179658	-2.060831
11	1	-4.331838	-0.326249	-1.106253
12	1	-3.400842	1.094898	-0.622349
13	6	-2.990417	-0.963869	1.230357
14	1	-2.339517	-1.604556	1.826776
15	1	-3.070811	0.004975	1.728662
16	1	-3.983886	-1.415214	1.198171
17	6	3.026181	-0.606216	-1.337695
18	1	4.001091	-1.09546	-1.374818
19	1	2.444582	-0.929581	-2.202234
20	1	3.18058	0.472465	-1.408966
21	6	3.108947	-0.531517	1.193449
22	1	3.24817	0.551515	1.201433
23	1	2.596362	-0.817645	2.112846
24	1	4.092157	-1.004462	1.183853
25	7	-0.069141	-1.012913	-0.134693
26	13	0.026764	1.348223	0.080939

Table S19: Cartesian coordinates for **1** adopting **TS-6** structure.

Center	Atomic	Coordinates (Å)		
Number	Number	X	Y	Z
1	7	-3.085356	2.466604	-0.024854
2	7	1.335484	0.103672	-0.127661
3	7	-0.853528	-0.086163	0.264657
4	7	0.475458	0.239479	0.733629
5	6	2.694154	0.406955	0.35932
6	6	-1.131689	-1.534873	0.232255
7	6	3.24714	1.545165	-0.494918

8	6	-2.631224	-1.764384	0.404731
9	6	-0.606523	-2.219823	-1.030973
10	1	2.638399	2.444417	-0.390827
11	1	4.264563	1.782177	-0.179869
12	1	3.267334	1.26191	-1.548811
13	1	-3.197007	-1.319375	-0.422038
14	1	-2.855079	-2.832078	0.400495
15	1	-2.994212	-1.340835	1.342315
16	1	0.45829	-2.040776	-1.164448
17	1	-0.776189	-3.298178	-0.975992
18	1	-1.12461	-1.831938	-1.911568
19	6	3.524933	-0.868424	0.235082
20	1	3.109277	-1.667429	0.850774
21	1	3.554831	-1.210148	-0.801191
22	1	4.54701	-0.677021	0.565538
23	1	-0.620596	-1.964384	1.102974
24	1	2.637511	0.715796	1.407924
25	13	-1.938045	1.262136	-0.130675
26	1	-3.400093	3.117244	-0.729882

Table S20: Cartesian coordinates for **1** adopting **I-6** structure.

Center	Atomic		Coordinates (Å)	
Number	Number	X	Y	Z
1	7	-2.054499	2.663801	-0.006979
2	7	1.102126	-0.920094	1.026732
3	7	-1.034182	-0.526005	0.320294
4	7	0.194303	-0.131875	0.724359
5	6	2.340661	-0.253608	1.458244
6	6	-1.406036	-1.931155	0.199167
7	6	3.459815	-0.649205	0.497067
8	6	-2.927605	-2.017432	0.183503
9	6	-0.774658	-2.558012	-1.04669
10	1	3.233176	-0.328132	-0.52071
11	1	4.39971	-0.185975	0.802237
12	1	3.593771	-1.732618	0.493499
13	1	-3.335633	-1.47802	-0.674127
14	1	-3.243026	-3.058949	0.110284
15	1	-3.355913	-1.590435	1.091225
16	1	0.310809	-2.474403	-1.01921
17	1	-1.034862	-3.616528	-1.103537
18	1	-1.1449	-2.064126	-1.947388
19	6	2.641317	-0.678739	2.894112
20	1	1.837289	-0.378521	3.567655
21	1	2.752883	-1.76293	2.954949

22	1	3.568232	-0.215906	3.237262
23	1	-1.01422	-2.443282	1.084847
24	1	2.196537	0.833203	1.422604
25	13	-1.164809	1.281541	0.288301
26	1	-1.758492	3.623485	0.10258

Table S21: Cartesian coordinates for **1** adopting **TS-7** structure.

Center	Atomic	Coordinates (Å)		
Number	Number	X	Y	Z
1	7	3.935084	-1.10531	-0.334347
2	7	-1.556533	0.007795	0.080424
3	7	0.604035	-0.318943	-0.079339
4	7	-0.649646	-0.771119	-0.261649
5	6	-2.896073	-0.556243	-0.125646
6	6	0.950296	1.023539	0.496554
7	6	-3.646848	0.333733	-1.114959
8	6	2.471239	0.925622	0.882853
9	6	0.671338	2.153452	-0.49004
10	1	-3.134621	0.362069	-2.078069
11	1	-4.658831	-0.043736	-1.274437
12	1	-3.716064	1.354948	-0.733943
13	1	2.968803	1.86464	0.642635
14	1	2.573849	0.759395	1.956142
15	1	3.526536	0.121718	0.337242
16	1	-0.373592	2.148866	-0.793244
17	1	0.896731	3.117111	-0.028757
18	1	1.296444	2.047718	-1.380526
19	6	-3.603541	-0.625123	1.226733
20	1	-3.061126	-1.273787	1.916481
21	1	-3.670499	0.369028	1.673756
22	1	-4.614898	-1.018719	1.107198
23	1	0.347223	1.161949	1.396438
24	1	-2.800535	-1.566152	-0.541091
25	13	2.270396	-0.917428	-0.215935
26	1	4.747075	-1.601602	-0.661995

Table S22: Cartesian coordinates for **1** adopting **I-7** structure.

Center	Atomic	Coordinates (Å)		
Number	Number	X	Y	Z
1	7	-1.490998	0.03738	0.096887
2	7	0.69327	-0.111609	-0.042553
3	7	-0.510267	-0.677446	-0.189726
4	6	-2.768597	-0.662474	-0.074383
5	6	0.927924	1.293361	0.441448

6	6	-3.590894	0.078685	-1.128006
7	6	2.471129	1.275441	0.692743
8	6	0.502649	2.345081	-0.577307
9	1	-3.072083	0.087637	-2.088048
10	1	-4.561675	-0.402466	-1.26507
11	1	-3.759425	1.113567	-0.821719
12	1	2.997071	2.049413	0.134805
13	1	2.721676	1.390437	1.747531
14	1	-0.563074	2.276022	-0.78587
15	1	0.723679	3.344408	-0.195733
16	1	1.051253	2.209086	-1.513518
17	6	-3.484155	-0.703865	1.27519
18	1	-2.891371	-1.249332	2.011436
19	1	-3.646685	0.308571	1.651416
20	1	-4.454049	-1.196696	1.179733
21	1	0.370585	1.436366	1.371716
22	1	-2.575131	-1.68619	-0.416694
23	13	2.43602	-0.554932	0.005562
24	7	3.355808	-2.001353	-0.404843
25	1	4.355049	-2.098908	-0.315979
26	1	2.952905	-2.853983	-0.762786

Table S23: Cartesian coordinates for **1** adopting **TS-8** structure.

Center	Atomic	Coordinates (Å)		
Number	Number	X	Y	Z
1	7	-1.663054	0.063229	0.064123
2	7	0.633418	-0.079982	-0.0306
3	7	-0.647387	-0.518635	-0.155222
4	6	-2.959536	-1.71175	-0.382036
5	6	0.937328	1.310313	0.433012
6	6	-3.638922	-1.737965	0.952409
7	6	2.486332	1.227162	0.626358
8	6	0.504347	2.37021	-0.571432
9	1	-3.94433	-0.739169	1.266302
10	1	-4.534882	-2.368338	0.913823
11	1	-2.980557	-2.151785	1.720064
12	1	3.021878	1.95717	0.020545
13	1	2.784025	1.364964	1.665683
14	1	-0.571275	2.328396	-0.738155
15	1	0.762964	3.364058	-0.200127
16	1	1.01295	2.217464	-1.527248
17	6	-1.995618	-2.639419	-0.755705
18	1	-1.857448	-2.865518	-1.808338
19	1	-0.859045	-1.710399	-0.515336

20	1	-1.767472	-3.45695	-0.076488
21	1	0.426145	1.478168	1.386284
22	1	-3.459302	-1.13984	-1.15418
23	13	2.350732	-0.617043	-0.01047
24	7	3.174717	-2.122768	-0.40379
25	1	4.168833	-2.275407	-0.335627
26	1	2.716378	-2.959735	-0.730946

Table S24: Cartesian coordinates for **1** adopting **I-8** structure.

Center	Atomic	Coordinates (Å)		
Number	Number	X	Y	Z
1	7	-2.899781	-0.08902	0.290562
2	1	-3.258969	0.832478	0.487034
3	7	2.000086	2.040616	-0.549355
4	7	0.318257	0.459913	-0.078355
5	6	1.174075	-0.725385	-0.359645
6	1	1.650674	-0.593239	-1.336044
7	1	0.14487	2.439032	0.046055
8	6	2.248213	-0.942027	0.700134
9	1	2.834497	-1.832283	0.462052
10	1	2.919461	-0.085701	0.748775
11	1	1.788323	-1.085141	1.681888
12	6	0.108349	-1.873234	-0.404346
13	1	0.3019	-2.648204	0.336434
14	1	-3.65376	-0.7586	0.28992
15	1	0.049151	-2.345954	-1.38456
16	7	0.895287	1.755071	-0.211997
17	13	-1.207769	-0.490942	-0.003971

Table S25: Cartesian coordinates for **1** adopting **TS-9** structure.

Center	Atomic	Coordinates (Å)		
Number	Number	X	Y	Z
1	7	2.607525	1.431092	-0.324542
2	1	2.478532	2.427776	-0.242177
3	7	-2.704908	1.526466	-0.424305
4	7	-0.327491	0.197948	0.604904
5	6	-0.370585	-1.290571	0.461351
6	1	-0.632991	-1.728279	1.433476
7	1	-1.220384	1.61127	1.094633
8	6	-1.417528	-1.727916	-0.563353
9	1	-1.458015	-2.817721	-0.629111
10	1	-2.410504	-1.361535	-0.297269
11	1	-1.169511	-1.333885	-1.553136
12	6	1.082865	-1.736657	0.05711

13	1	1.109915	-2.31036	-0.870712
14	1	3.544964	1.232112	-0.638716
15	1	1.580141	-2.317766	0.834777
16	7	-1.783649	1.520748	0.221189
17	13	1.386342	0.192426	0.024000

Table S26: Cartesian coordinates for **1** adopting **I-9** structure.

Center	Atomic	Coordinates (Å)		
Number	Number	X	Y	Z
1	7	2.836777	-0.137119	0.332841
2	1	3.294535	-1.005817	0.103371
3	7	-0.322054	-1.052036	-0.395084
4	6	-1.368051	-0.010756	-0.429308
5	1	-1.841827	0.035217	-1.420418
6	6	-2.460912	-0.226171	0.615467
7	1	-3.221021	0.557487	0.559024
8	1	-2.955748	-1.189311	0.466989
9	1	-2.027697	-0.222076	1.619259
10	6	-0.540147	1.287516	-0.187787
11	1	-0.839253	1.835031	0.705404
12	1	3.486516	0.632668	0.312334
13	1	-0.539886	1.97438	-1.033358
14	13	1.065102	0.109308	-0.028068
15	1	-0.590349	-2.018026	-0.502255

Table S27: Cartesian coordinates for geometry of **TS-10**

Center	Atomic	Coordinates (Å)		
Number	Number	X	Y	Z
1	7	-1.920817	-0.601592	0.612931
2	1	-2.689807	-0.170291	1.10377
3	7	0.185197	1.756133	-0.574016
4	6	1.755316	-0.323563	-0.402429
5	1	2.155188	0.389265	-1.108489
6	6	2.380367	-0.344396	0.950749
7	1	3.370755	-0.807463	0.864356
8	1	2.518893	0.666376	1.329202
9	1	1.799506	-0.926597	1.66783
10	6	0.808168	-1.220426	-0.829366
11	1	0.52299	-2.049084	-0.188514
12	1	-2.081371	-1.592605	0.521995
13	1	0.597005	-1.31941	-1.888509
14	13	-0.630169	0.31793	-0.197445
15	1	-0.016657	2.719328	-0.340454

Table S28: Cartesian coordinates for **1** adopting **I-10A** structure.

Center Number	Atomic Number	Coordinates (Å) X	Y	Z
1	7	0.99739	-1.825441	0.005208
2	1	1.709992	-2.400949	-0.416998
3	7	1.546357	1.513391	-0.424803
4	6	-1.328735	0.669206	0.035154
5	1	-1.135069	1.635991	-0.417383
6	6	-2.183492	-0.286483	-0.732413
7	1	-3.200395	0.116254	-0.784186
8	1	-1.835121	-0.394484	-1.760605
9	1	-2.224093	-1.271098	-0.267086
10	6	-0.82622	0.440584	1.272478
11	1	-1.043322	-0.481952	1.801473
12	1	0.376447	-2.396839	0.555811
13	1	-0.336137	1.23565	1.82022
14	13	1.037604	-0.04524	-0.015267
15	1	2.423292	1.790055	-0.846922

Table S29: Cartesian coordinates for **1** adopting **I-10** structure.

Center Number	Atomic Number	Coordinates (Å) X	Y	Z
1	7	1.041008	-1.86688	0.038513
2	1	1.498616	-2.650539	-0.400147
3	7	1.584629	1.378142	-0.775973
4	1	0.314505	-2.186045	0.660022
5	13	1.487178	-0.182504	-0.201562
6	1	2.165147	2.142803	-0.463824

Cartesian coordinates for selected intermediate and transition state structures of 6

Table S30: Cartesian coordinates for geometry of **6**.

Center Number	Atomic Number	Coordinates (Å) X	Y	Z
1	7	-2.124506	-1.265761	-0.206894
2	7	-1.660051	-0.440083	-1.087205
3	6	-2.582487	-0.124624	-2.199505
4	6	-2.994375	-1.401926	-2.947195
5	1	-3.492043	-2.096618	-2.270948
6	1	-3.683093	-1.155534	-3.757813
7	1	-2.129895	-1.90497	-3.379879
8	6	-3.841946	0.567054	-1.654274
9	1	-3.57764	1.485412	-1.131901
10	1	-4.521917	0.820706	-2.47018
11	1	-4.366319	-0.087585	-0.957554

12	7	-1.302233	-1.337945	0.784058
13	6	-1.634744	-2.316719	1.839611
14	6	-1.117353	-3.701979	1.424897
15	1	-0.039262	-3.677452	1.270492
16	1	-1.34013	-4.44182	2.196927
17	1	-1.591733	-4.020474	0.495755
18	7	-0.743696	1.722481	0.743971
19	7	0.749525	1.72022	-0.743893
20	7	0.00419	2.466069	0.000144
21	6	-1.715118	2.45099	1.591793
22	6	1.723175	2.445747	-1.591725
23	6	-1.002718	3.04822	2.814891
24	6	2.772087	1.433923	-2.049012
25	1	-0.194737	3.709369	2.498036
26	1	-1.705268	3.627412	3.418073
27	1	-0.581738	2.26366	3.442116
28	1	2.307488	0.631909	-2.617433
29	1	3.510342	1.923104	-2.686567
30	1	3.28716	0.988114	-1.199723
31	7	1.297917	-1.341764	-0.784081
32	7	1.658607	-0.445177	1.087235
33	7	2.120474	-1.27217	0.206835
34	6	1.6272	-2.321585	-1.839675
35	6	2.582037	-0.1326	2.199533
36	6	0.90952	-1.875401	-3.113334
37	6	2.990111	-1.411181	2.947131
38	6	3.843539	0.555347	1.654327
39	1	1.283132	-0.914835	-3.467382
40	1	1.056671	-2.610148	-3.90639
41	1	-0.156173	-1.77695	-2.925606
42	1	3.485661	-2.10733	2.270826
43	1	3.679602	-1.166915	3.757737
44	1	2.124138	-1.91163	3.379835
45	1	3.581936	1.474521	1.132027
46	1	4.524297	0.806921	2.470223
47	1	4.365939	-0.10079	0.957537
48	6	1.104707	-3.705011	-1.425238
49	1	1.577805	-4.025361	-0.496081
50	1	0.026688	-3.676575	-1.270965
51	1	1.324888	-4.445561	-2.197333
52	6	1.012567	3.044921	-2.81492
53	1	0.589239	2.261521	-3.44201
54	1	0.206561	3.708518	-2.49815

55	1	1.716829	3.621928	-3.418204
56	6	-2.76699	1.442311	2.049225
57	1	-3.283392	0.997917	1.200001
58	1	-2.304753	0.639005	2.617759
59	1	-3.503793	1.933744	2.686728
60	6	-0.915123	-1.873199	3.113095
61	1	0.15087	-1.778575	2.92507
62	1	-1.285166	-0.911294	3.46726
63	1	-1.064717	-2.607429	3.906173
64	13	-0.000186	-0.013556	-0.000024
65	6	-3.14783	-2.381273	2.085998
66	1	-3.677535	-2.7435	1.206591
67	1	-3.352268	-3.059088	2.916888
68	1	-3.542729	-1.396952	2.338306
69	6	-1.840698	0.829618	-3.131298
70	1	-1.559568	1.737983	-2.60032
71	1	-0.932961	0.37005	-3.521353
72	1	-2.475891	1.104682	-3.974768
73	6	-2.389284	3.579628	0.797021
74	1	-3.163193	4.050448	1.406118
75	1	-1.666794	4.340485	0.50596
76	1	-2.854812	3.191361	-0.108964
77	6	1.843122	0.8238	3.131392
78	1	1.56469	1.732984	2.600404
79	1	0.934021	0.366912	3.52143
80	1	2.479136	1.096953	3.974862
81	6	2.400623	3.572488	-0.797092
82	1	3.175921	4.04094	-1.406247
83	1	1.680378	4.335511	-0.506143
84	1	2.864998	3.182981	0.108952
85	6	3.140103	-2.391562	-2.085708
86	1	3.668309	-2.755883	-1.206264
87	1	3.342291	-3.069926	-2.9167
88	1	3.538613	-1.408616	-2.337702

Table S31: Cartesian coordinates for **6** adopting **I-1** structure.

Center	Atomic	Coordinates (Å)		
Number	Number	X	Y	Z
1	7	-0.532487	2.096858	1.219927
2	7	-0.716515	0.895275	1.659071
3	6	-1.319722	0.732824	2.994613
4	6	-2.743189	0.182332	2.808616
5	1	-2.730109	-0.737434	2.225536
6	1	-3.200651	-0.028196	3.777607

7	1	-3.365263	0.909666	2.287183
8	6	-0.475042	-0.27957	3.776443
9	1	0.510647	0.128246	3.997148
10	1	-0.96272	-0.532509	4.719558
11	1	-0.34642	-1.196197	3.203319
12	7	-0.178513	2.021837	-0.020815
13	6	0.296512	3.244635	-0.683986
14	6	-0.322634	4.501292	-0.064973
15	1	-0.018643	4.627254	0.973035
16	1	-0.002529	5.378811	-0.629522
17	1	-1.412429	4.450242	-0.092871
18	7	-2.106922	-0.327198	-0.778359
19	7	-0.996611	-1.809683	0.213993
20	7	-2.116582	-1.558764	-0.379921
21	6	-3.303316	0.203846	-1.453088
22	6	-0.772696	-3.20426	0.637277
23	6	-2.961939	0.422485	-2.93468
24	6	0.671199	-3.280429	1.137476
25	1	-2.706572	-0.523772	-3.41255
26	1	-3.811782	0.860357	-3.462181
27	1	-2.112158	1.096426	-3.037778
28	1	0.832547	-2.602681	1.976057
29	1	0.900824	-4.293245	1.471591
30	1	1.37208	-3.009342	0.348337
31	7	3.047539	-0.412332	-0.20846
32	7	1.080636	-0.386035	-1.340999
33	7	1.80699	-0.243875	-0.24126
34	6	3.696981	-0.188463	1.100952
35	6	1.606244	-0.830477	-2.644446
36	6	4.762245	0.891681	0.862703
37	6	0.387446	-0.912494	-3.566104
38	6	2.615297	0.182372	-3.210504
39	1	4.292987	1.84074	0.598788
40	1	5.364164	1.041942	1.761627
41	1	5.424267	0.600233	0.046017
42	1	-0.080585	0.064715	-3.682865
43	1	0.68469	-1.268742	-4.553685
44	1	-0.358314	-1.597916	-3.160443
45	1	3.49158	0.257986	-2.571038
46	1	2.935269	-0.133362	-4.206312
47	1	2.161396	1.170243	-3.299289
48	6	4.377654	-1.51472	1.471284
49	1	3.631632	-2.293895	1.637811

50	1	5.038124	-1.841728	0.666883
51	1	4.969191	-1.402441	2.382566
52	6	-1.75109	-3.583173	1.758878
53	1	-1.616576	-2.941564	2.629715
54	1	-2.780484	-3.480828	1.413257
55	1	-1.592542	-4.617935	2.069384
56	6	-3.641018	1.551078	-0.799542
57	1	-3.95414	1.408922	0.234325
58	1	-2.773552	2.209758	-0.801465
59	1	-4.452178	2.041933	-1.340448
60	6	1.829239	3.294717	-0.563551
61	1	2.121221	3.343764	0.486814
62	1	2.280076	2.404462	-1.000945
63	1	2.22883	4.173619	-1.074213
64	13	-0.282739	0.037641	-0.108427
65	6	-0.103455	3.136849	-2.159025
66	1	-1.187367	3.176565	-2.272098
67	1	0.329568	3.958167	-2.732113
68	1	0.252375	2.198373	-2.583169
69	6	-4.496845	-0.745955	-1.328839
70	1	-4.734142	-0.943155	-0.282801
71	1	-5.367251	-0.287522	-1.801341
72	1	-4.302191	-1.701416	-1.814441
73	6	2.253389	-2.221382	-2.53787
74	1	2.569763	-2.555699	-3.528461
75	1	3.121206	-2.202786	-1.883536
76	1	1.537054	-2.945869	-2.149744
77	6	2.749253	0.242111	2.21769
78	1	2.229759	1.167981	1.97187
79	1	1.995754	-0.519463	2.403825
80	1	3.313903	0.396393	3.139131
81	6	-1.372014	2.056123	3.762379
82	1	-1.994809	2.791213	3.253886
83	1	-1.789063	1.874269	4.754512
84	1	-0.375673	2.483814	3.878141
85	6	-0.961646	-4.155255	-0.554268
86	1	-0.737175	-5.182185	-0.259568
87	1	-1.988089	-4.115751	-0.918422
88	1	-0.297756	-3.879385	-1.373919

Table S32: Cartesian coordinates for **6** adopting **TS-2** structure.

Center	Atomic	Coordinates (Å)		
Number	Number	X	Y	Z
1	7	2.591506	-1.288508	0.102057

2	7	1.023723	-0.39864	1.342854
3	6	3.697686	-2.275816	0.021855
4	6	4.682978	-2.079274	1.178324
5	1	4.208305	-2.27239	2.138746
6	1	5.525095	-2.763979	1.063511
7	1	5.068067	-1.058026	1.185304
8	6	3.10524	-3.692049	0.057258
9	1	2.404532	-3.841529	-0.765245
10	1	3.89532	-4.441187	-0.023054
11	1	2.569517	-3.852676	0.993916
12	6	0.492165	-0.261076	2.736456
13	6	0.68603	1.199742	3.168552
14	1	0.201633	1.884887	2.475716
15	1	0.263295	1.359596	4.162203
16	1	1.74934	1.44413	3.202046
17	6	-0.993315	-0.636327	2.706985
18	1	-1.11881	-1.680249	2.416811
19	1	-1.434967	-0.494361	3.694678
20	1	-1.555725	-0.032072	2.001254
21	7	-1.267854	-0.637006	-0.830402
22	6	-0.687628	-1.688698	-1.711631
23	7	-3.296554	-1.514193	-0.724915
24	6	-1.41835	-1.818073	-3.053511
25	6	0.721002	-1.094066	-1.900766
26	6	-4.663842	-1.348812	-0.157631
27	1	-1.551068	-0.833209	-3.506336
28	1	-0.818804	-2.427902	-3.734589
29	1	-2.396871	-2.276732	-2.932986
30	1	1.390831	-1.83692	-2.341809
31	1	0.685952	-0.283466	-2.63964
32	1	1.73648	-1.092457	-0.829938
33	6	-5.465545	-2.542281	-0.681948
34	6	-4.616153	-1.385151	1.375877
35	1	-5.008934	-3.480158	-0.360296
36	1	-6.493129	-2.512136	-0.313331
37	1	-5.488233	-2.537278	-1.773212
38	1	-4.05299	-0.538139	1.76642
39	1	-5.62542	-1.351014	1.792449
40	1	-4.133214	-2.302246	1.719727
41	7	-0.508926	2.171079	0.075101
42	7	1.3968	1.844308	-0.752351
43	6	2.738813	2.289792	-1.16982
44	6	-1.702177	3.03033	0.254016

45	6	-2.477003	3.032821	-1.074272
46	1	-2.747467	2.015004	-1.355697
47	1	-3.391163	3.623479	-0.984575
48	1	-1.864027	3.464075	-1.868053
49	6	-1.299818	4.457373	0.641351
50	1	-0.694327	4.454736	1.54977
51	1	-0.727567	4.944712	-0.146133
52	1	-2.199737	5.045069	0.829525
53	6	3.707573	2.05723	0.000706
54	1	4.72291	2.346374	-0.279094
55	1	3.405111	2.651561	0.864658
56	1	3.713077	1.006981	0.289419
57	6	3.149579	1.436218	-2.374119
58	1	3.122961	0.377673	-2.128374
59	1	2.476922	1.609298	-3.215726
60	1	4.164397	1.689056	-2.685874
61	7	2.079931	-1.136254	1.263045
62	7	-2.515915	-0.59774	-0.384364
63	7	0.545068	2.750326	-0.397759
64	13	0.206103	0.336159	-0.283007
65	6	1.218015	-1.178452	3.72404
66	1	1.131469	-2.225914	3.435314
67	1	2.276793	-0.937148	3.803913
68	1	0.759543	-1.052638	4.706356
69	6	4.408171	-2.032031	-1.311716
70	1	3.71937	-2.12406	-2.152712
71	1	4.851748	-1.036327	-1.340186
72	1	5.204158	-2.765154	-1.448727
73	6	-0.608399	-3.046101	-0.998542
74	1	-1.605659	-3.424599	-0.785853
75	1	-0.08623	-3.771305	-1.628046
76	1	-0.061642	-2.956827	-0.057781
77	6	-2.57127	2.420064	1.354106
78	1	-2.842609	1.398311	1.096396
79	1	-2.053603	2.421897	2.313979
80	1	-3.487844	3.001901	1.463396
81	6	2.741654	3.770288	-1.562362
82	1	2.494971	4.412143	-0.718008
83	1	3.735768	4.039313	-1.923136
84	1	2.020127	3.966414	-2.356585
85	6	-5.296364	-0.038965	-0.645499
86	1	-4.738813	0.821047	-0.276559
87	1	-5.298272	-0.001185	-1.736844

88	1	-6.328619	0.041826	-0.297144

Table S33: Cartesian coordinates for **6** adopting **I-2** structure.

Center	Atomic	Coordinates (Å)		
Number	Number	X	Y	Z
1	7	-1.049125	-0.001057	0.679505
2	6	-1.39631	-0.018098	2.158286
3	7	-3.111424	0.006839	-0.108137
4	6	-2.162235	-1.286918	2.540798
5	6	0.055015	-0.022821	2.76374
6	6	-3.981198	0.009885	-1.307062
7	1	-1.603126	-2.175308	2.237392
8	1	-2.296095	-1.323612	3.624721
9	1	-3.138762	-1.313564	2.061649
10	1	0.239809	0.852363	3.387761
11	1	0.240376	-0.908483	3.372584
12	6	-3.239607	0.127461	-2.640554
13	6	-4.775193	-1.303727	-1.256419
14	1	-2.560116	-0.710656	-2.794476
15	1	-3.963976	0.143161	-3.457709
16	1	-2.648162	1.042773	-2.683231
17	1	-5.289617	-1.402308	-0.299206
18	1	-5.51897	-1.335703	-2.055827
19	1	-4.106063	-2.158637	-1.373057
20	7	2.039193	1.052644	-0.052774
21	7	2.036547	-1.048166	-0.070833
22	6	2.522835	-2.37561	-0.480316
23	6	2.516961	2.385082	-0.455749
24	6	1.622305	3.385613	0.279305
25	1	1.712635	3.266956	1.36096
26	1	1.908696	4.406043	0.022969
27	1	0.57526	3.244	0.004551
28	6	2.36262	2.548049	-1.974109
29	1	2.978218	1.819269	-2.502366
30	1	1.323077	2.400638	-2.271282
31	1	2.672128	3.548758	-2.280646
32	6	3.973595	-2.566495	-0.01983
33	1	4.329235	-3.561295	-0.293567
34	1	4.624994	-1.826854	-0.486258
35	1	4.050193	-2.459183	1.063695
36	6	1.603806	-3.385098	0.211249
37	1	1.660732	-3.283097	1.296929
38	1	0.566061	-3.2371	-0.093544
39	1	1.896535	-4.402156	-0.051301

40	7	-1.889853	0.010018	-0.356112
41	7	2.641406	0.005408	-0.521854
42	13	0.741511	-0.004772	0.929229
43	6	-2.166236	1.239335	2.569294
44	1	-3.144518	1.272115	2.094112
45	1	-2.296583	1.252953	3.654198
46	1	-1.611524	2.135975	2.282498
47	6	-4.935226	1.199039	-1.126781
48	1	-5.449702	1.132339	-0.166912
49	1	-4.382234	2.140269	-1.151135
50	1	-5.683139	1.217734	-1.922578
51	6	3.981866	2.566141	-0.039154
52	1	4.329916	3.564868	-0.308137
53	1	4.092831	2.441254	1.039492
54	1	4.616889	1.833687	-0.538056
55	6	2.41656	-2.515347	-2.004752
56	1	1.386913	-2.362209	-2.332029
57	1	3.049217	-1.779854	-2.502468
58	1	2.734671	-3.511856	-2.315941

Table S34: Cartesian coordinates for **6** adopting **TS-3** structure.

Center	Atomic	Coordinates (Å)		
Number	Number	X	Y	Z
1	7	-1.017022	-0.02399	1.055491
2	6	-1.250088	-0.057202	2.541782
3	7	-3.194324	-0.012077	0.256321
4	6	-1.992657	-1.329927	2.953012
5	6	0.244553	-0.063356	3.029045
6	6	-3.606942	0.036727	-1.948242
7	1	-1.444735	-2.215315	2.622447
8	1	-2.086988	-1.372372	4.040276
9	1	-2.989112	-1.355499	2.512565
10	1	0.4754	0.805266	3.646371
11	1	0.480611	-0.956262	3.608615
12	6	-2.299061	0.0517	-2.431342
13	6	-4.402867	-1.238103	-1.951897
14	1	-1.899334	0.977166	-2.836723
15	1	-1.67501	0.022528	-1.075463
16	1	-1.893745	-0.851873	-2.878405
17	1	-5.131111	-1.252946	-1.139291
18	1	-4.952064	-1.3391	-2.896423
19	1	-3.757178	-2.111978	-1.848519
20	7	1.91373	1.055183	-0.00928
21	7	1.920348	-1.046354	-0.049832

22	6	2.250946	-2.363534	-0.619677
23	6	2.234015	2.395298	-0.529381
24	6	1.590253	3.383945	0.445111
25	1	2.000066	3.263236	1.449838
26	1	1.777166	4.40785	0.12036
27	1	0.509634	3.234341	0.493026
28	6	1.622341	2.55292	-1.928885
29	1	2.063646	1.835655	-2.621737
30	1	0.544954	2.381367	-1.898382
31	1	1.802888	3.559139	-2.310925
32	6	3.771447	-2.551963	-0.667668
33	1	4.01489	-3.53851	-1.06531
34	1	4.233629	-1.799478	-1.306769
35	1	4.201518	-2.467223	0.331931
36	6	1.610737	-3.3927	0.314376
37	1	2.016837	-3.307793	1.324258
38	1	0.529052	-3.2518	0.364618
39	1	1.80515	-4.40228	-0.048575
40	7	-2.007902	-0.006037	0.134775
41	7	2.395991	0.01726	-0.617934
42	13	0.79098	-0.021755	1.144751
43	6	-4.410685	1.305498	-1.894159
44	1	-3.770381	2.177775	-1.751322
45	1	-4.960743	1.445766	-2.833153
46	1	-5.138796	1.279056	-1.081726
47	6	-2.000216	1.19183	3.008206
48	1	-2.997189	1.230311	2.569892
49	1	-2.09387	1.186434	4.096342
50	1	-1.458041	2.094106	2.716059
51	6	1.644764	-2.47176	-2.026212
52	1	0.566048	-2.30939	-1.992786
53	1	2.082942	-1.725309	-2.689585
54	1	1.833882	-3.461264	-2.445914
55	6	3.753077	2.595433	-0.574849
56	1	4.18696	2.475892	0.419534
57	1	4.218071	1.870706	-1.2433
58	1	3.988766	3.597881	-0.935685

Table S35: Cartesian coordinates for **6** adopting **I-4** structure.

Center	Atomic	Coordinates (Å)		
Number	Number	X	Y	Z
1	7	0.834469	-1.74497	-1.130296
2	6	1.484012	-2.576039	-0.075942
3	6	2.998752	-2.658649	-0.297735

4	6	1.131483	-1.764299	1.226637
5	1	3.43536	-1.657862	-0.300854
6	1	3.483912	-3.247141	0.485296
7	1	3.221968	-3.12514	-1.262015
8	1	0.538694	-2.349237	1.930964
9	1	2.022275	-1.417136	1.751608
10	1	0.919986	-2.063973	-2.080912
11	7	-1.552719	0.305901	0.091237
12	7	0.215897	1.417103	-0.122508
13	6	1.001375	2.659123	-0.197275
14	6	-3.002107	0.143777	0.286613
15	6	-3.250404	-1.365535	0.331728
16	1	-2.710089	-1.822829	1.162967
17	1	-4.313965	-1.568837	0.462042
18	1	-2.917913	-1.841128	-0.592606
19	6	-3.757229	0.774022	-0.891277
20	1	-3.556166	1.844314	-0.946832
21	1	-3.44893	0.316062	-1.832754
22	1	-4.832695	0.629995	-0.772906
23	6	0.827039	3.469546	1.094345
24	1	1.437453	4.373817	1.06003
25	1	-0.215214	3.761104	1.226947
26	1	1.132578	2.87971	1.960388
27	6	2.458892	2.221542	-0.35813
28	1	2.782658	1.628334	0.499364
29	1	2.585147	1.616227	-1.257454
30	1	3.108232	3.094436	-0.43486
31	7	-1.067353	1.505815	0.032964
32	13	0.210427	-0.533279	0.005179
33	6	0.884698	-3.986763	-0.042217
34	1	1.041629	-4.494888	-0.998489
35	1	1.340332	-4.593812	0.744378
36	1	-0.191108	-3.936122	0.137473
37	6	-3.430016	0.794662	1.608998
38	1	-2.883599	0.356022	2.445845
39	1	-3.230407	1.866454	1.591647
40	1	-4.49796	0.644434	1.777562
41	6	0.554006	3.482526	-1.412476
42	1	0.671744	2.904685	-2.33084
43	1	-0.49407	3.768003	-1.317197
44	1	1.153436	4.390819	-1.496565

Table S36: Cartesian coordinates for **6** adopting **TS-5** structure.

Center	Atomic	Coordinates (Å)

Number	Number	X	Y	Z
1	7	0.394068	1.699398	1.81791
2	6	1.47843	2.578586	-0.316828
3	6	2.902831	2.35045	0.079076
4	6	0.821483	1.709307	-1.165026
5	1	3.209519	1.314926	-0.057987
6	1	3.536676	2.980872	-0.55819
7	1	3.061801	2.634648	1.115957
8	1	-0.074962	2.044817	-1.676043
9	1	1.376132	0.906315	-1.640338
10	1	0.386555	1.686801	2.825685
11	7	-1.444067	-0.415204	-0.182712
12	7	0.464207	-1.255059	0.076064
13	6	1.416474	-2.373073	0.15874
14	6	-2.870249	-0.458294	-0.540207
15	6	-3.379821	0.97855	-0.405541
16	1	-2.857522	1.641518	-1.098441
17	1	-4.446299	1.022835	-0.630174
18	1	-3.221645	1.353823	0.606816
19	6	-3.618641	-1.380307	0.432974
20	1	-3.234462	-2.398738	0.364595
21	1	-3.493598	-1.032764	1.459801
22	1	-4.685112	-1.396315	0.200035
23	6	1.347808	-3.240379	-1.103438
24	1	2.09089	-4.037855	-1.04854
25	1	0.362909	-3.692915	-1.213452
26	1	1.550477	-2.640568	-1.992738
27	6	2.802543	-1.737393	0.292542
28	1	3.049296	-1.157944	-0.59947
29	1	2.840222	-1.071985	1.156992
30	1	3.562691	-2.509661	0.416956
31	7	-0.751926	-1.50712	-0.282615
32	13	0.050232	0.61878	0.561903
33	6	0.912919	3.927777	-0.016136
34	1	1.331106	4.634018	-0.747194
35	1	-0.170924	3.937893	-0.105073
36	1	1.181979	4.251027	0.985152
37	6	-3.039413	-0.951053	-1.983323
38	1	-2.497137	-0.303641	-2.675171
39	1	-2.656443	-1.965998	-2.091532
40	1	-4.094439	-0.947549	-2.263389
41	6	1.097095	-3.211318	1.405871
42	1	1.135998	-2.590555	2.3025

43	1	0.096755	-3.639573	1.326884
44	1	1.815348	-4.026294	1.516156

Table S37: Cartesian coordinates for **6** adopting **I-7** structure.

Center	Atomic	Coordinates (Å)		
Number	Number	X	Y	Z
1	7	-1.512864	-0.03071	0.091639
2	7	0.690785	-0.114967	0.108237
3	7	-0.490131	-0.711954	-0.099679
4	6	-2.786167	-0.748108	-0.15531
5	6	0.940278	1.320276	0.558237
6	6	-3.51934	0.062966	-1.23309
7	6	2.512471	1.323969	0.59004
8	6	0.407283	2.3269	-0.46393
9	1	-2.967848	0.034184	-2.174901
10	1	-4.518028	-0.343777	-1.405655
11	1	-3.617157	1.105689	-0.927055
12	1	2.937133	2.053699	-0.099293
13	1	2.904555	1.534525	1.584937
14	1	-0.677374	2.277346	-0.52969
15	1	0.699907	3.337864	-0.170983
16	1	0.823759	2.124948	-1.453473
17	6	-3.565143	-0.681634	1.165977
18	1	-3.046488	-1.244396	1.944613
19	1	-3.663522	0.352185	1.500698
20	1	-4.564778	-1.10381	1.043114
21	13	2.433196	-0.536686	0.012512
22	7	3.331028	-1.990234	-0.422556
23	1	4.335397	-2.0743	-0.425924
24	1	2.907619	-2.862177	-0.70159
25	6	0.360423	1.57807	1.950893
26	1	-0.725261	1.511862	1.938737
27	1	0.743853	0.847368	2.666793
28	1	0.652093	2.574223	2.291628
29	6	-2.626289	-2.202216	-0.603574
30	1	-2.065031	-2.270266	-1.535713
31	1	-2.097366	-2.792072	0.145308
32	1	-3.612899	-2.643239	-0.75945

Table S38: Cartesian coordinates for **6** adopting **TS-8** structure.

Center	Atomic	Coordinates (Å)		
Number	Number	X	Y	Z
1	7	1.250047	-0.920717	-0.000246
2	7	-0.853897	0.04768	0.000022

3	7	0.507346	0.010298	-0.00003
4	6	3.125667	0.358649	-0.000033
5	6	-1.717658	-1.194298	-0.000198
6	6	3.666186	-0.229359	1.272279
7	6	-3.14146	-0.529751	-0.00001
8	6	-1.4807	-2.024045	-1.263009
9	1	3.604204	-1.31868	1.263734
10	1	4.721492	0.04472	1.394286
11	1	3.124851	0.139006	2.145196
12	1	-3.721888	-0.799156	-0.882055
13	1	-3.721822	-0.799515	0.881969
14	1	-0.457591	-2.396687	-1.292979
15	1	-2.165687	-2.874183	-1.282301
16	1	-1.656641	-1.421287	-2.156755
17	6	2.532885	1.618631	0.00025
18	1	2.544667	2.203316	-0.915316
19	1	1.139638	1.101033	0.00018
20	1	2.544719	2.20293	0.916062
21	13	-2.233869	1.197806	0.000309
22	7	-2.372273	2.953698	0.000673
23	1	-3.238722	3.46886	0.000809
24	1	-1.589578	3.590137	0.000774
25	6	3.666113	-0.228823	-1.272624
26	1	3.124728	0.139912	-2.145355
27	1	4.721411	0.045306	-1.394575
28	1	3.604128	-1.318147	-1.264535
29	6	-1.480606	-2.024558	1.262258
30	1	-2.165591	-2.874703	1.281255
31	1	-0.457495	-2.39721	1.292001
32	1	-1.656482	-1.422162	2.156261
